# Synthesis of a Janus pentiptycene containing two thiophene rings using an intramolecular Fagan–Nugent reaction

**DOI:** 10.1039/d6cc04649e

**Published:** 2026-08-27

**Authors:** Stephanie Montanaro, Rebecca L. Melvin, Mark R. J. Elsegood, Iain A. Wright

**Affiliations:** a Chemistry Department, Loughborough University Loughborough Leicestershire LE11 3TU UK; b EaStCHEM and School of Chemistry, University of Edinburgh, Joseph Black Building, David Brewster Road Edinburgh EH9 3FJ UK iain.wright@ed.ac.uk

## Abstract

Zirconocene-mediated synthesis of a triptycene and a pentiptycene where one or two, respectively, of the aryl rings of the iptycene are replaced by [*c*]-fused thiophenes is demonstrated. A 9,10-disubstituted anthracene bearing silyl-protected *cis*-1,2-dichloroethylene and acetylene groups has also been isolated and a mechanism for its formation proposed.

## Abstract 2

Organozirconium chemistry facilitates the synthesis of heterocyclic iptycenes through the Fagan–Nugent reaction, and a silyl protected dihaloethylene substituted anthracene.

## Graphical abstract

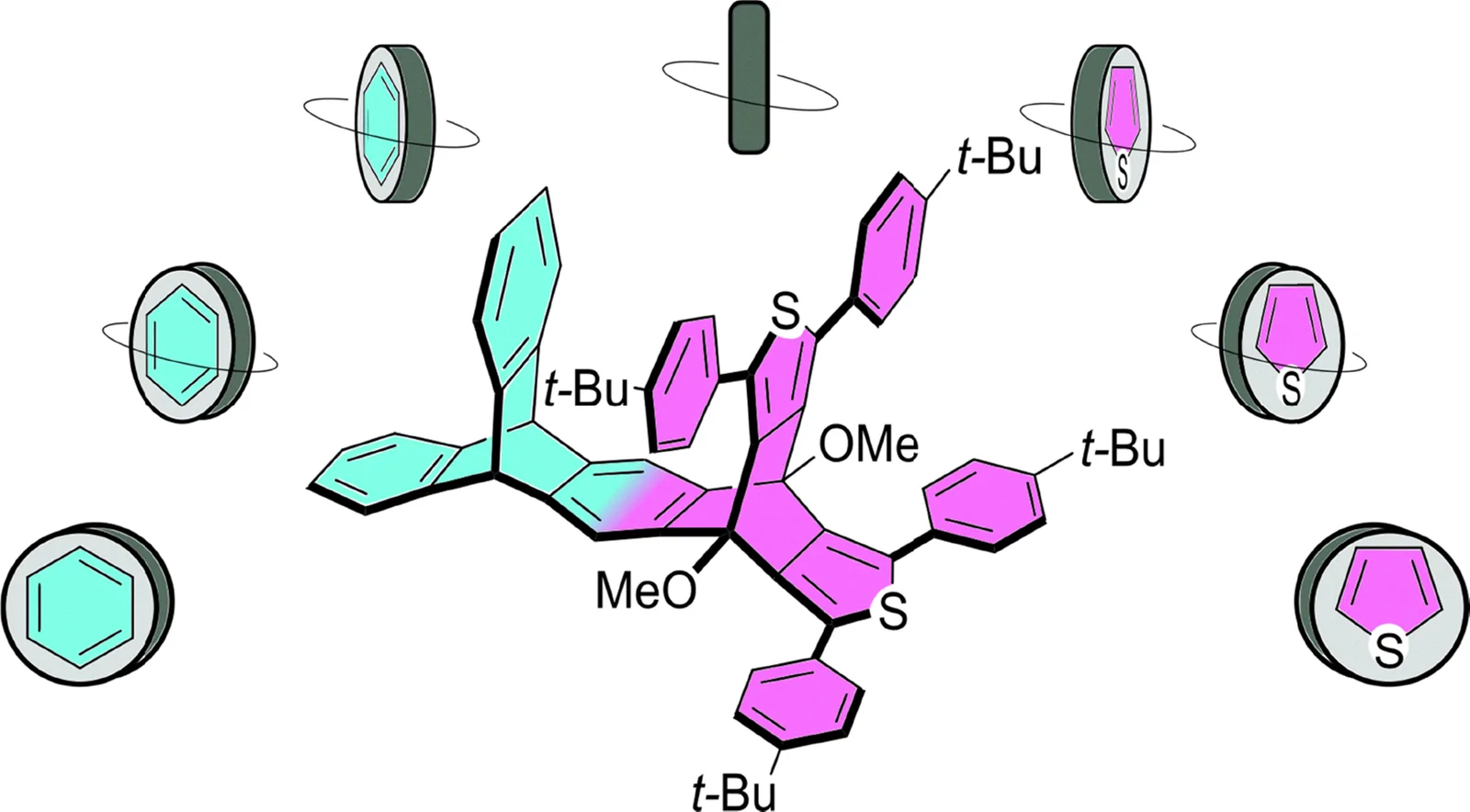

Triptycene is a widely utilised three-dimensional scaffold in organic materials and supramolecular chemistry.^1,2^ Fusing of new rings directly onto the periphery of existing iptycene scaffolds has been the standard approach towards the synthesis of π-extended iptycenes for several decades.^3^ The 3D core of triptycene and all higher iptycenes is the bicyclo[2.2.2]octa-2,5,7-triene carbocycle, hereafter referred to as “barrelene”.^4^ For the purposes of this article, we will draw a distinction between “on-iptycene” and “on-barrelene” ring-fusion. In an on-iptycene system, new rings are fused onto one or more of the aryl rings of the iptycene, while for an on-barrelene iptycene one or more of the fused aryl rings of the iptycene is itself replaced by a heterocycle. Far fewer of the latter class of molecules have been prepared, and their synthesis to date revolves around a diverse range of reactivities.^5^

The Fagan–Nugent reaction^6^ utilizes five-membered metallocycles formed inter/intramolecularly between two acetylenes by organozirconium reagents such as the zirconocene dichloride (Cp_2_ZrCl_2_) derived Negishi reagent Cp_2_Zr(η^2^-but-1-ene)^7^ to produce a variety of cyclic (and acyclic) products and introduce a wide range of heteroatoms.^8^

Here, we demonstrate that the Fagan–Nugent reaction can be applied to a dihydroacene bearing suitably configured acetylene groups to form on-barrelene fused thiophene heterocycles, thereby producing S-heterocyclic iptycenes. Specifically, we have synthesised a triptycene 1 which features a single thiophene ring in place of an aryl ring, and a pentiptycene 2. The central benzene ring of 2 is flanked by a dibenzobarrelene on one side and a di-[*c*]-thieno-fused barrelene on the other, resulting in a two-faced, Janus-type structure. While a limited number of on-barrelene thieno-triptycenes,^5*a*,*c*,*e*,*f*,*g*,*i*,9^ have previously been synthesised, we are not aware of any such congeners for pentiptycene. Indeed, heterocyclic pentiptycenes of any sort are an underrepresented class of compounds, with existing examples consisting of cyclic dimers or oligomers rather than discrete molecules.^10^ Compound 1 is also a member of a broader class of materials that has recently been used as a component of high-performance polymers of intrinsic microporosity for CO_2_ photoreduction^11^ and also luminescent polymers,^12^ therefore new routes to synthesise and diversify this class of materials are required.

Compound 1 was synthesised first as shown in Scheme 1. Using the procedure reported by Taylor and Swager, anthraquinone was reacted with excess lithium phenylacetylide to generate a mixture of the *cis*- and *trans*- isomers of diol 3. The reaction was performed using toluene as the solvent to favour the formation of *cis*-3 which was isolated *via* column chromatography where it eluted after *trans*-3.^13^ Due to the requirement of using *n*-BuLi in forming the Negishi reagent and S_2_Cl_2_ in the ensuing Fagan–Nugent [2 + 2 + 1] cycloaddition, the alcohols were protected with methyl groups through deprotonation with sodium hydride at reduced temperature, followed by addition of methyl iodide to give *cis*-4 in 85% yield. The Negishi reagent was then generated prior to addition of *cis*-4 to form a deep red solution of intermediate complex 5 which is not isolated. After 3 hours, 5 was treated with S_2_Cl_2_ prior to working up. Using column chromatography 1 was isolated as a colourless solid in 38% yield.

**Scheme 1:**
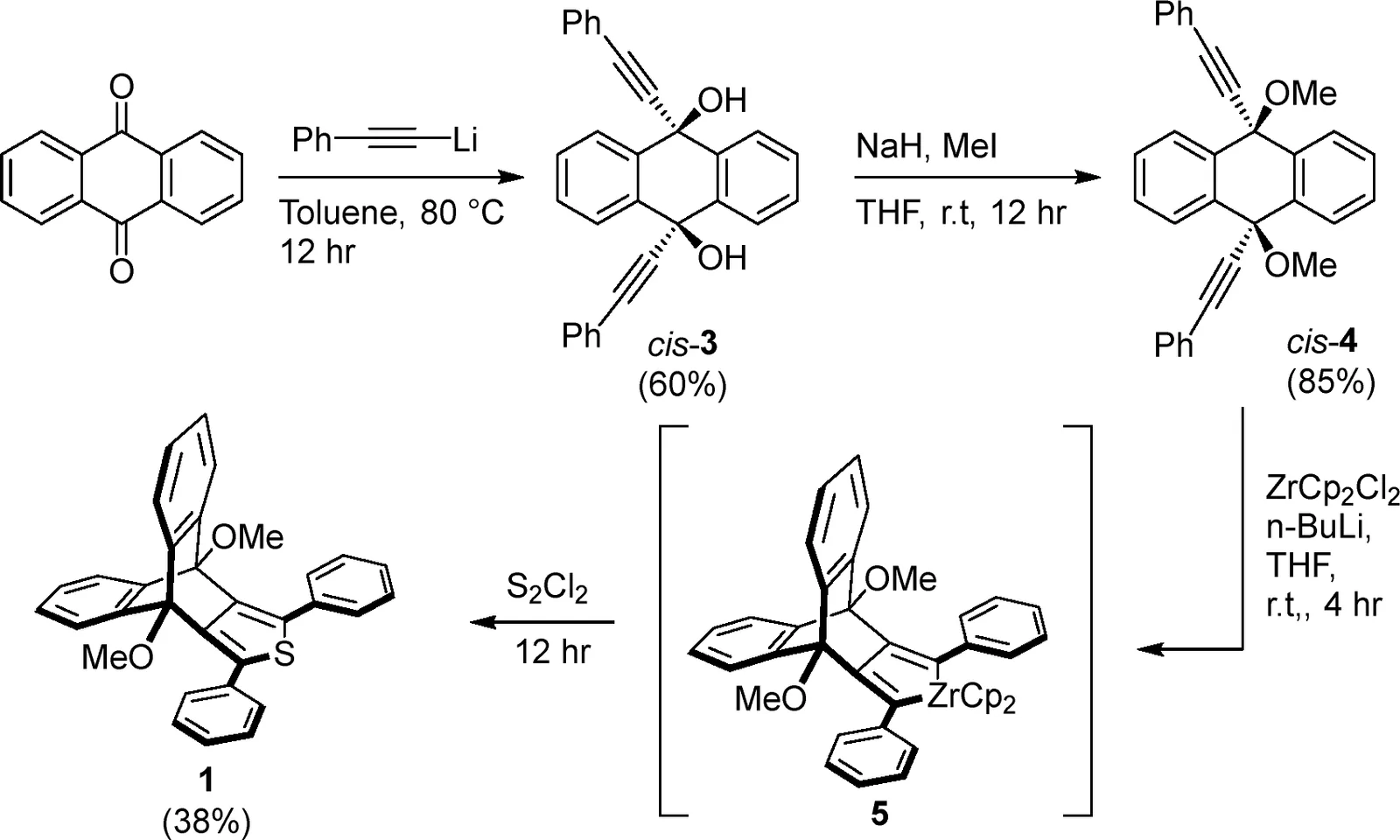
Synthetic pathway to triptycene 1.

The initial concept being proven, we embarked upon the synthesis of Janus pentiptycene 2 (Scheme 2). Dibrominated [*c*]-thieno-fused quinone 6 was prepared *via* a Friedel–Crafts acylation between 2,5-dibromo-thiophene-3,4-dicarbonyl chloride and triptycene. This reaction forms a mixture of two isomers which can be separated by exploiting differences in their solubilities. In contrast to our previous report which used thiophene-3,4-dicarbonyl chloride, two-fold addition of triptycene was not observed.^14^ Suzuki–Miyaura cross-coupling of 6 was then performed with 4-*tert*-butylphenylboronic acid to form quinone 7 as small, yellow needles in 71% yield after purification. The 4-*tert*-butylphenyl groups were selected to ensure good solubility for the final product. Compound 7 was then treated with an excess of lithium 4-*tert*-butylphenylacetylide to produce *cis*-diol 8 which was again protected *via* methylation forming 9 as confirmed by ^1^H NMR spectroscopy. Finally, 9 was subject to the Fagan–Nugent cycloaddition reaction with S_2_Cl_2_ to successfully produce the desired on-barrelene, thiophene-fused pentiptycene 2. The isolated yield of 17% for two-fold ring formation is consistent with the yield obtained for the single-ring formation in 1. A single crystal of the mixed solvate 2·1.5(CHCl_3_) 3(CH_3_OH) permitted unambiguous structural determination using X-ray crystallography (Fig. 1).

**Scheme 2:**
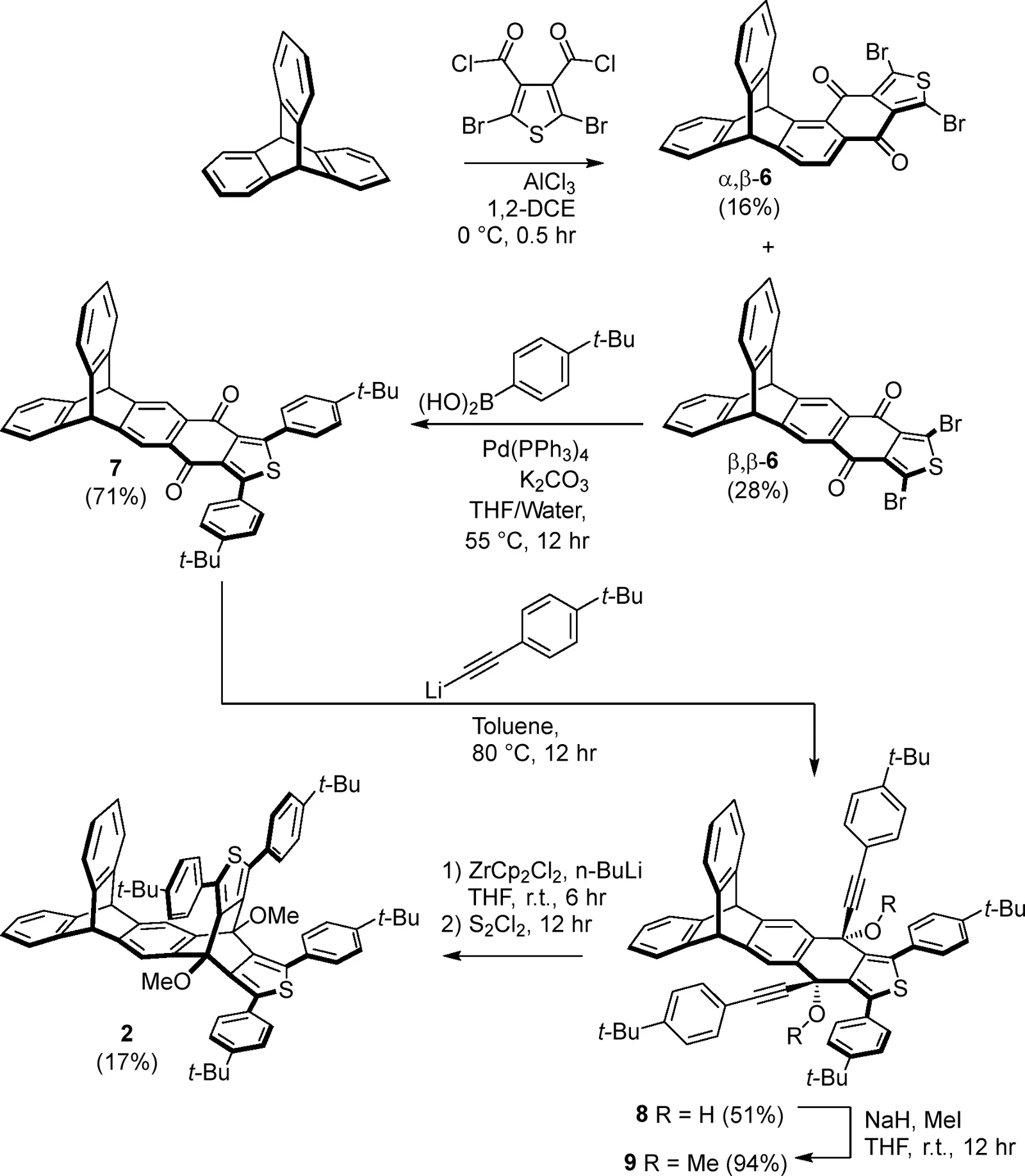
Synthetic pathway to Janus pentiptycene 2.

**Fig. 1:**
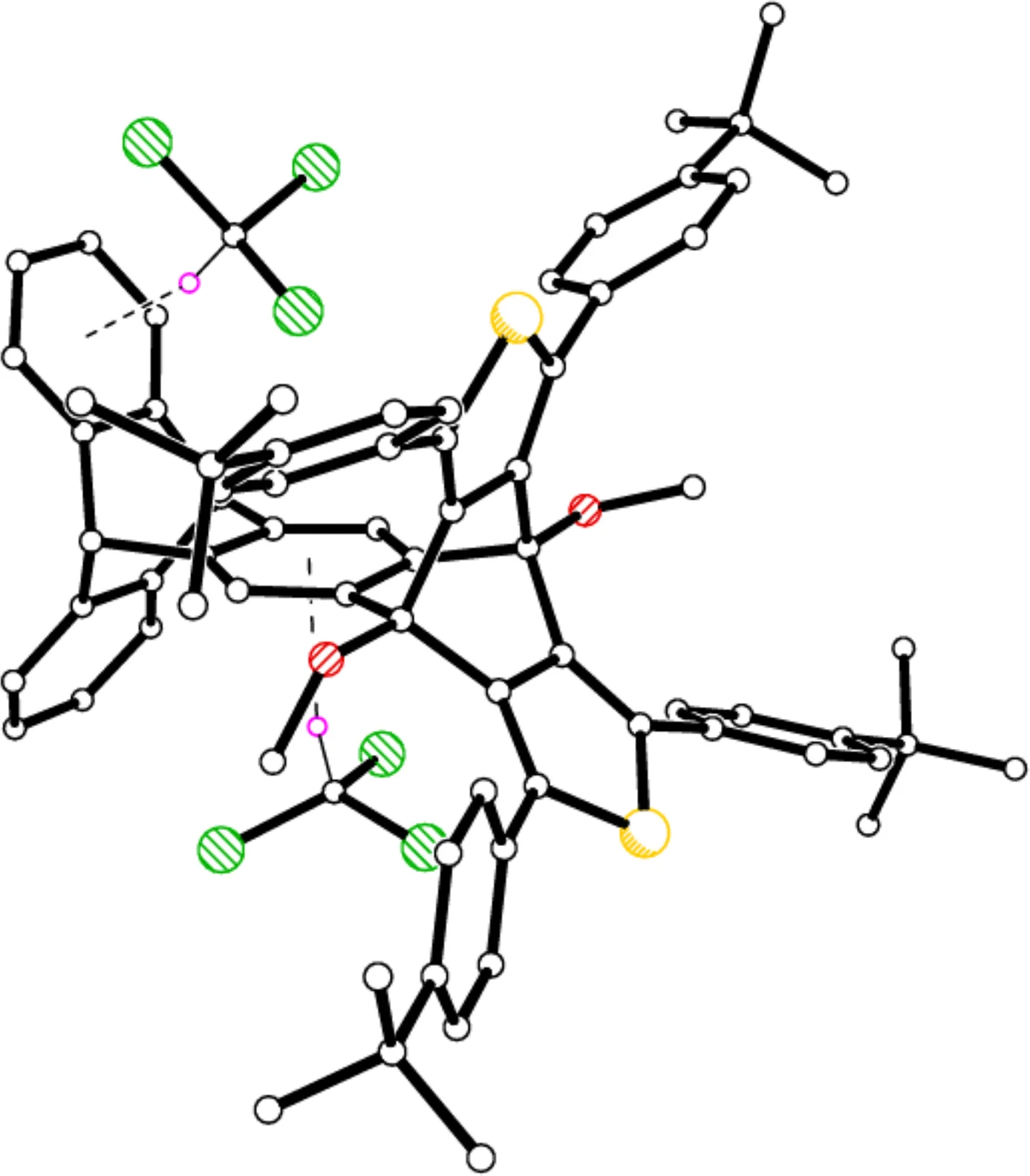
X-Ray crystallography determined molecular structure of the solvate 2·1.5(CHCl_3_) 3(CH_3_OH). C = open black circles, H = pink, S = yellow, O = red, Cl = green.

The absorption spectra for 1 and 2 in toluene solution are shown in Fig. S2. Comparing with literature spectra available for 2,5-diphenylthiophene (PTP) in both CH_2_Cl_2_ and 2-MeTHF,^15^ the spectra of 1 and 2 are blue-shifted significantly, despite the presence of the terminal electron-donating *tert*-butyl substituents. This suggests that the effective conjugation length of both molecules 1 and 2 is reduced compared with PTP.

Density-functional theory (ORCA v5, B3LYP/def2-SVP) was used to generate optimised structures of 1, 2, and PTP which confirm that a more planar geometry is assumed by PTP (Fig. 2). In 1 and 2, interactions with the bridgehead −OMe substituents has increased the dihedral angle between one of the terminal phenyl rings on the thiophene(s), thereby reducing the effective conjugation length of the PTP moieties.

**Fig. 2:**
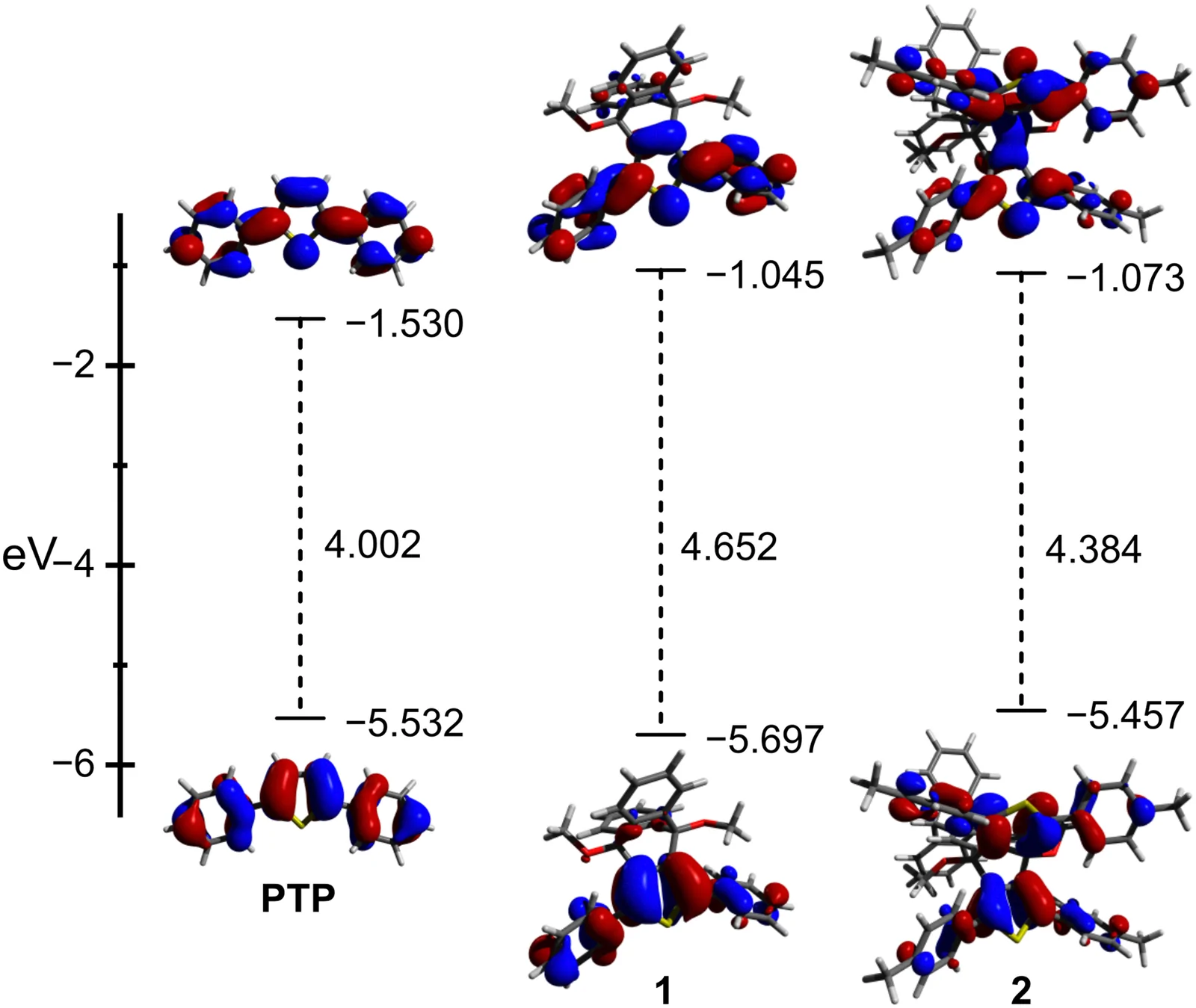
Density functional theory calculated frontier orbital energies and profiles, showing the highest occupied (lower) and lowest unoccupied (upper) molecular orbitals and the energy gap between these (central).

Although gratified that the pathway had proven successful, the relatively low yields observed in the synthesis of 1 and 2 led us to examine whether changing the steric bulk of the acetylene might affect the efficiency of the reaction, so the reaction pathway was attempted again using the bulkier triisopropylsilyl (TIPS) protected acetylene in place of phenylacetylene (Scheme 3). Again, the stereoisomers of the intermediate diol 10 were separated cleanly *via* column chromatography, displaying the same trends of *cis*-10 eluting more slowly than *trans*-10 and the chemical shift of the −OH proton further downfield in *cis*-10 compared with *trans*-10. The structures of both stereoisomers were unambiguously assigned using X-ray crystallography (Fig. 3), with both having packing motifs dictated by inter- and intramolecular hydrogen bonds occurring from the −OH moieties. We note the lower yield of *cis*-10 compared with both 3 and 8, which may suggest that increasing the steric hindrance is detrimental to this procedure.

**Scheme 3:**
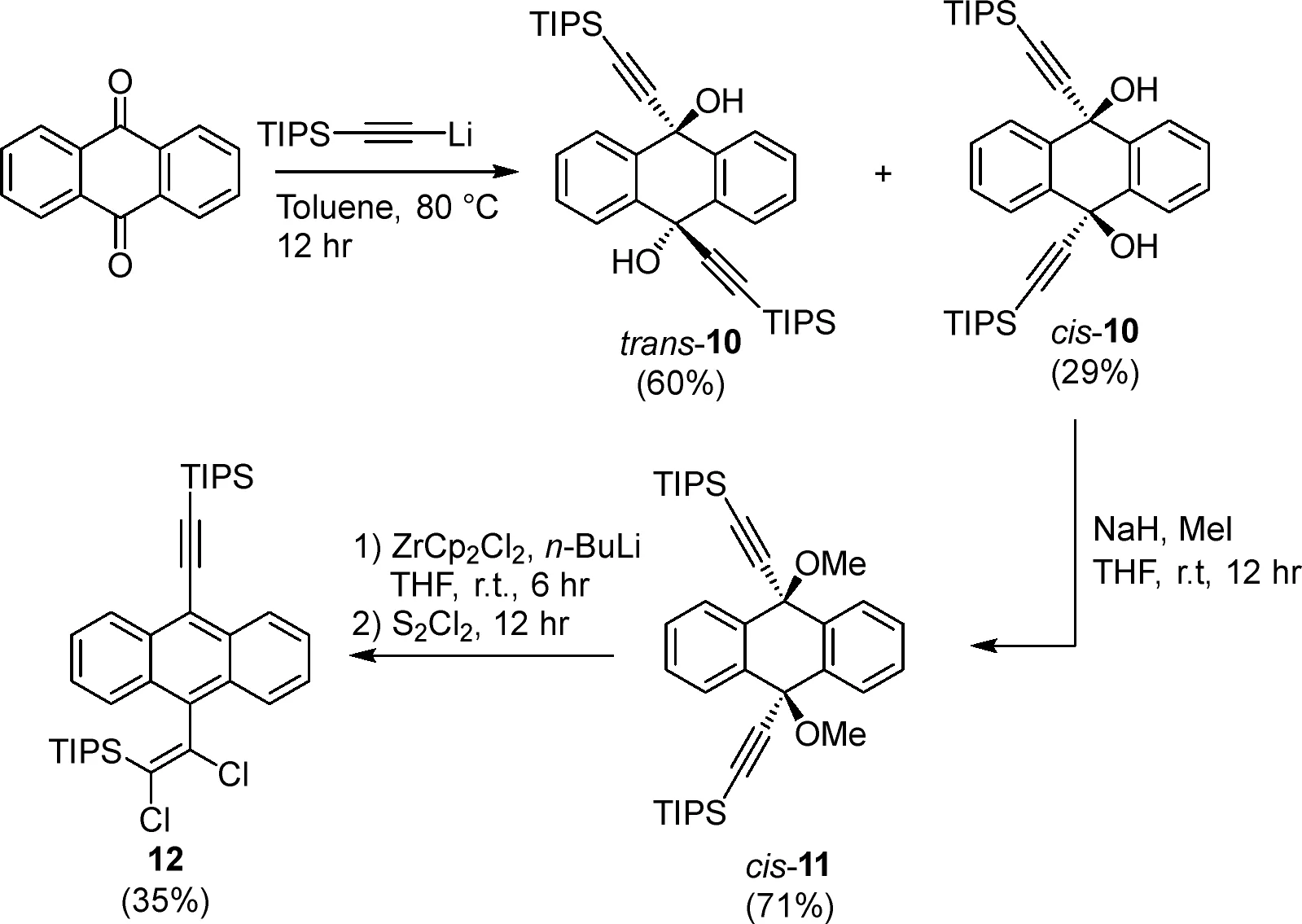
Serendipitous synthesis of *cis*-dichloroethylene-functionalised anthracene 12.

**Fig. 3:**
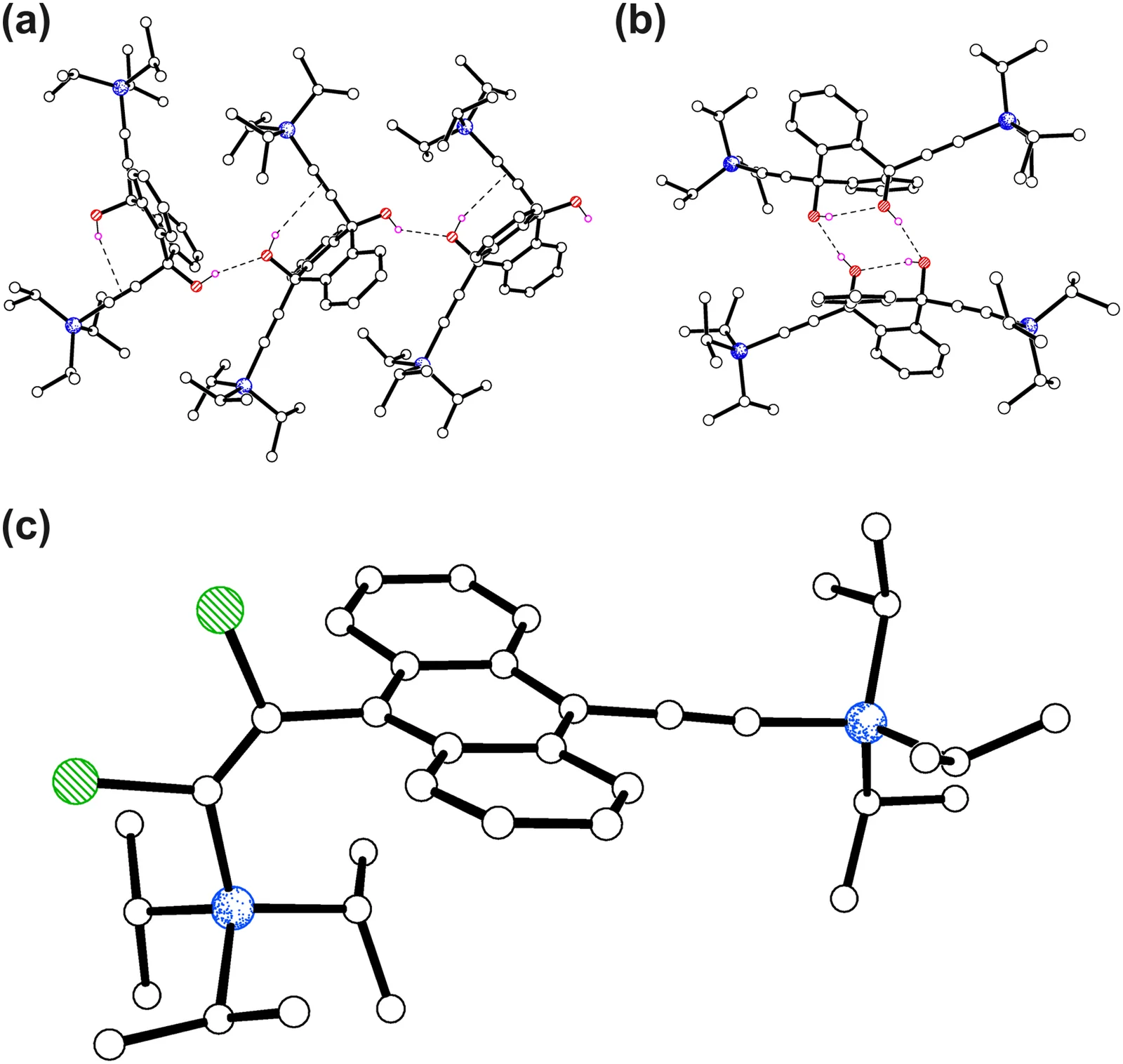
Crystal structures of (a) *trans*-10, (b) *cis*-10, and (c) 12. C = open black circles, H = pink, O = red, Cl = green Si = blue.

The Negishi reagent was generated and applied to the methylated compound *cis*-11 then treated with S_2_Cl_2_. A yellow crystalline solid was isolated from the reaction. The ^1^H-NMR spectrum of this compound clearly indicated that iptycene formation had not occurred and the product obtained was ultimately identified using X-ray crystallography as anthracene 12 which had been isolated in 35% yield. In 12 one TIPS-acetylene group remained unchanged while the other had been converted selectively to a TIPS-protected *cis*-dichloroethylene. The UV-vis absorption and emission spectra of 12 are shown in Fig. S2 and reveal an identical 0–0 transition wavelength to 9,10-bis[((triisopropyl)silyl)ethynyl]anthracene but blue-shifted peaks for the 0–1 and 0–2 transitions.^16^ Considering the wide interest in silylethynyl-functionalised acenes, for applications in charge transport technologies,^17^ solar energy conversion^18^ and photocatalysis^19^ this transformation may present new opportunities to increase structural diversity and optimise properties.

To explain the formation of 12, we first recognise that zirconocycle formation is a reversible process which leads to an equilibrium between a 5-membered (η^4^) and a more reactive 3-membered (η^2^) zirconocycle.^8*a*,20^ To form 12 either the equilibrium must have shifted to favour the η^2^-zirconocycle, or the S_2_Cl_2_ is only able to react with the less sterically-congested η^2^-zirconocycle. It has also been shown that dipolar reactants such as carbonyls and nitriles undergo insertion reactions into one of the C–Zr bonds of η^2^-zirconocycles to form 5-membered intermediates which can then be converted into a range of products.^21^ Based on these findings, we propose that the mechanism shown in Scheme 4 is plausible. An initial association of S_2_Cl_2_ with the η^2^-zirconocycle 13 facilitates nucleophilic addition of the first chlorine onto the outermost carbon of the double bond. This leads to the loss of the first −OMe group to form intermediate 14. A second equivalence of S_2_Cl_2_ then reacts with the other carbon of the metallocycle, eliminating the second −OMe group and in doing so forming the aromatic anthracene ring system in 15. The metallocycle then collapses to eliminate Zr(Cp)_2_ and form the distinctive *cis*-dichloroethylene group in 12.

**Scheme 4:**
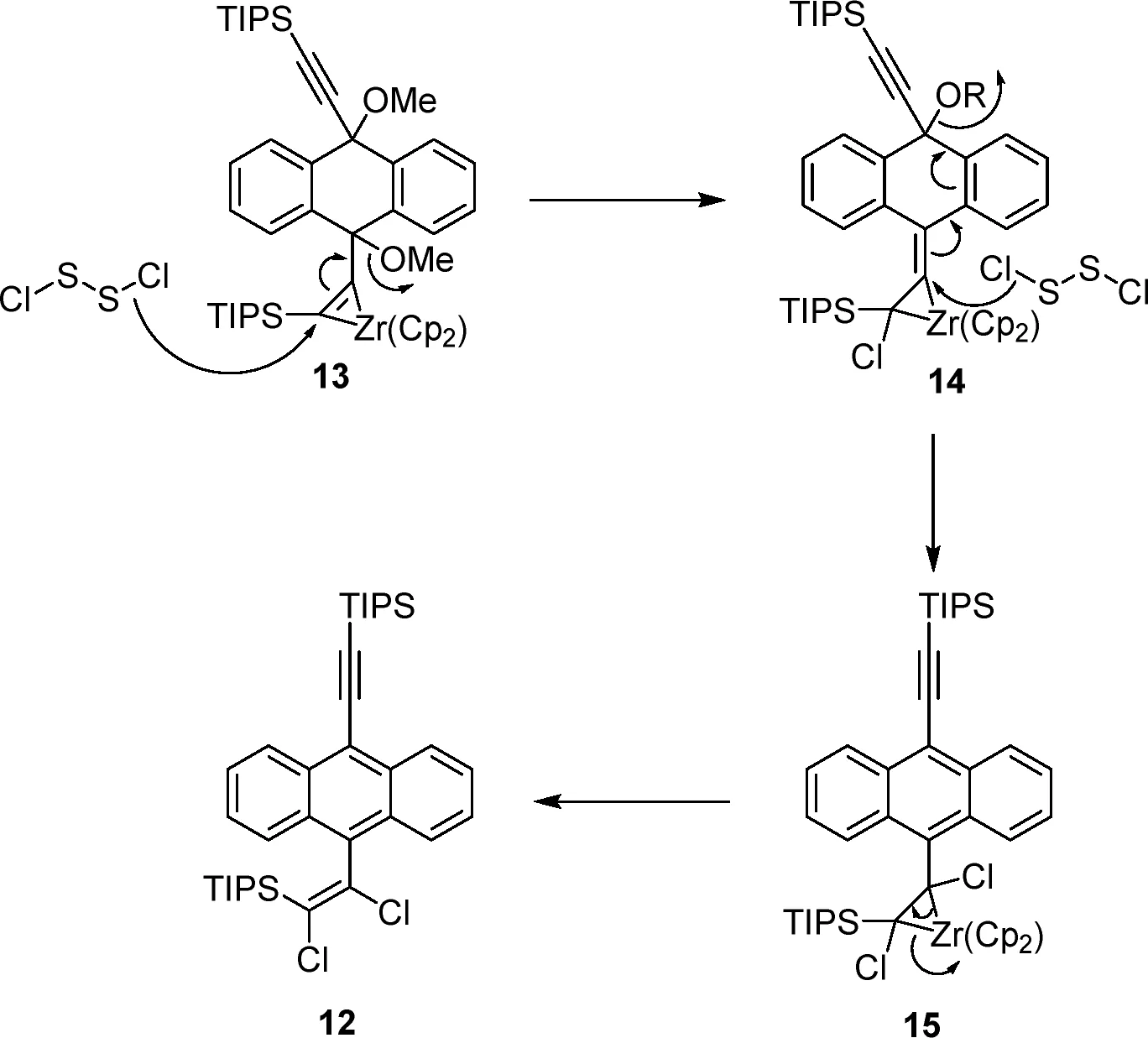
Proposed mechanism for the formation of 12.

While this mechanism seems plausible, and examples can be found where larger silyl groups do appear to have a negative influence on 5-membered heterocycle formation reactions^22^ or steer reactions towards products derived from the η^2^-zirconocycle,^8*a*,20*b,d,e*,23^ we note that there are other examples where large groups make little difference.^20*d,e*^ An additional factor in the reactions presented here is the increased ring strain involved in forming the barrelene ring-system; the cited examples all produce planar heterocycles. So, some caution should be exerted in drawing firm conclusions about the implications of using larger silyl groups at this time. Finally, the formation of 12 presents an interesting and useful orthogonality with the products that have previously been isolated from the direct reaction of S_2_Cl_2_ with di-*tert*-butyl- and diadamantyl-bearing acetylenes. These include episulfides, dithietes, and thioxoethanones, all of which are the result of addition of sulfur rather than chlorine atoms to the alkynes as observed here.^24^

In conclusion we have presented a synthetic route to produce on-barrelene fused heterocyclic iptycenes as demonstrated by the synthesis of 1 and 2. This has been achieved through the Fagan–Nugent reaction of intramolecular zirconocycles. The amenability of the Fagan–Nugent reaction to introduce a wide range of heteroatoms or other functionalities means that this approach offers a general route to the synthesis of diverse and useful new heterocyclic iptycenes. In completing this work, the serendipitous synthesis of a *cis*-dichloroethylene bearing anthracene 12 has also been established which may provide a convenient entry-point to the synthesis of new acenes with otherwise challenging to implement structural tropes.

## Conflicts of interest

There are no conflicts to declare.

## Supplementary Material

CC-062-D6CC04649E-s001

CC-062-D6CC04649E-s002

## Data Availability

Other data supporting this article have been included as part of the supplementary information (SI). Supplementary information: includes synthetic methods, NMR and mass spectra, UV-vis and emission spectroscopy, and additional X-ray crystallography details and packing diagrams. See DOI: https://doi.org/10.1039/d6cc04649e. CCDC 2545297 (2), 2545298 (*cis*-10) 2545299 (*trans*-10) and 2545300 (12) contain the supplementary crystallographic data for this paper.^25*a*–*d*^
