# Supplementary material for: Synthesis of a Janus pentiptycene containing two thiophene rings using an intramolecular Fagan–Nugent reaction
Source: Chem Commun (Camb). 2026 Aug 27;62(79):19867–71. doi: 10.1039/d6cc04649e (PMC13575914; doi:10.1039/d6cc04649e)
Supplement: Supplementary file 1 [file CC-062-D6CC04649E-s001.pdf]

Supplementary Information (SI) for ChemComm.
This journal is © The Royal Society of Chemistry 2026

Supporting Information for

Synthesis of a Janus Pentiptycene Containing Two Thiophene Rings using an Intramolecular Fagan-Nugent Reaction

Stephanie Montanaro,[a] Rebecca L. Melvin,[b] Mark R. J. Elsegood,[a] and Iain A. Wright*[b]

a. *Chemistry Department, Loughborough University, Loughborough, Leicestershire, LE11 3TU, UK.*

b. *EaStCHEM and School of Chemistry, University of Edinburgh, Joseph Black Building, David Brewster Road, Edinburgh, EH9 3FJ, UK.* Email: iain.wright@ed.ac.uk

Table of Contents

**General Methods**	**S3**
**Synthetic Methods**	**S4**
**^{1}H and ^{13}C NMR Spectra**	**S15**
**HRMS Spectra**	**S28**
**UV-vis and Emission Spectroscopy**	**S35**
**X-Ray Crystallography**	**S36**
**References**	**S43**

General Methods

All reactants and reagents were purchased from commercial suppliers and used without further purification unless otherwise stated. Column chromatography was carried out using silica gel 60, 40−60 μm mesh (Fluorochem) and Aluminium oxide 90 active neutral 0.063−0.200 mm (70−230 mesh ASTM, Merck). Analytical thin-layer chromatography was performed on precoated aluminum silica gel 60 F254 plates (Merck) and Macherey-Nagel ALUGRAM Alox N/UV_{254} Aluminum Sheets (Fisher), which were approximately 2 cm × 6 cm in size, and visualized using ultraviolet light (254/365 nm).

NMR spectra were recorded on Jeol ECS 400 MHz and Jeol ECZ 500 MHz spectrometers. Additional spectra were recorded on a Bruker Avance III 500 MHz spectrometer. Chemical shifts are reported in ppm downfield of tetramethylsilane (TMS) using TMS or the residual solvent as an internal reference. NMR spectra were processed using MestReNova. Multiplicities are reported as singlet (s), doublet (d), triplet (t), and multiplet (m). Melting points were determined in open-ended capillaries using a Stuart Scientific SMP10 melting point apparatus at a ramping rate of 1 °C/min. They are recorded to the nearest 1 °C and are uncorrected.

Atmospheric solids analysis probe (ASAP) mass spectra were recorded on a Waters LCT Premier XE spectrometer. The sample was introduced as a solid, applied directly to a glass probe tip. Matrix-assisted laser desorption ionisation (MALDI) and laser desorption ionisation (LDI) mass spectra were recorded using Bruker Daltonik Solarix 12T spectrometer running in positive ion reflectron mode. Samples were prepared in CH_2Cl_2 with *trans*-2-[3-(4-*tert*-butylphenyl)-2-methyl2-propenylidene]malononitrile or dithranol as the matrix. Electrospray ionisation (ESI) mass spectra were recorded using a Bruker Microtof II instrument.

UV−vis absorbance spectra were measured using a UV-1800 UV−vis spectrophotometer (Shimadzu) and UVProbe version 2.33 software. Emission spectra were recorded on a SPEX Fluoromax luminescence spectrometer using dM300 version 3.12 software.

Synthetic Methods

cis-**3**

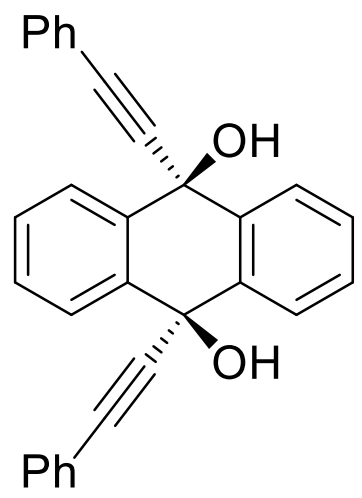

n-BuLi (6.00 mL, 2.5 M in hexanes, 15 mmol) was added dropwise to a solution to phenylacetylene (1.75 mL, 16 mmol) in toluene (50 mL) at 0 °C. The mixture was warmed to room temperature and stirred for 45 minutes. 9,10-Anthraquinone (1.04 g, 5.00 mmol) was added as a solid in one portion, and the resulting mixture was heated to 80 °C and stirred overnight. The reaction mixture was quenched with saturated NH_4Cl (aq.) at 0 °C, then diluted with EtOAc (50 mL) and water (100 mL). The organic phase was separated, and the aqueous phase extracted once with EtOAc (50 mL). The combined organic extracts were washed with water (150 mL) then brine (100 mL), then dried over $MgSO_4$, before the solvent was removed under reduced pressure. The residue was purified by column chromatography on SiO_2 (10-20% EtOAc/hexane), yielding pure *cis*-**3** as a pale-yellow solid (1.24 g, 60%). Characterisation in agreement with literature.[1]

^{1}H NMR (500 MHz, acetone-d_6, ppm) δ = 8.16 (dd, J = 5.9, 3.3 Hz, 4H), 7.49 (dd, J = 5.9, 3.3 Hz, 4H), 7.33–7.28 (m, 6H), 7.26–7.21 (m, 4H), 6.05 (s, 2H).

^{13}C NMR (126 MHz, acetone-d_6, ppm) δ = 139.9, 132.4, 129.4, 129.2, 127.3, 123.5, 92.3, 86.5, 68.4.

HRMS (ESI) *m*/*z* for $C_{30}H_{20}O_2$ $[M+Na]^+$ calcd. 435.1356, found 435.1355.

cis-**4**

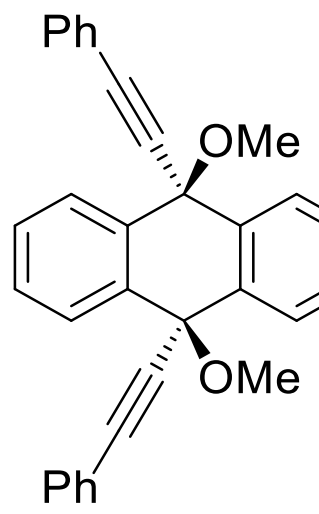

cis-**3** (825 mg, 2.00 mmol) in anhydrous THF (10 mL) was added to a stirred suspension of NaH (60 wt% suspension in paraffin, 200 mg, 5.00 mmol) in anhydrous THF (10 mL) at −78 °C. The suspension was stirred for 30 min at the same temperature. Methyl iodide (0.62 mL,10 mmol) was added dropwise slowly with a syringe and the mixture was allowed to warm to room temperature and stirred overnight. The reaction mixZture was poured into ice-cold saturated NH_4Cl (aq.) solution (50 mL). The organic layer was separated, and the aqueous phase was extracted with Et_2O (3 × 25 mL). The combined organic extracts were washed thoroughly with water (2 × 50 mL) and finally with saturated brine solution (50 mL), then dried over $MgSO_4$, and the solvent removed under reduced pressure. The residue was purified by column chromatography on SiO_2 (50% EtOAc/hexane), yielding *cis*-**4** as a pale-yellow solid (750 mg, 85%). Method adapted from literature procedure.[2]

^{1}H NMR (400 MHz, chloroform-*d*, ppm) δ = 8.10 (dd, J = 5.9, 3.4 Hz, 4H), 7.51 (dd, J = 5.9, 3.4 Hz, 4H), 7.45–7.42 (m, 4H), 7.31–7.23 (m, 6H), 3.09 (s, 6H).

^{13}C NMR (101 MHz, chloroform-*d*, ppm) δ = 135.8, 131.9, 129.1, 128.8, 128.6, 128.3, 122.6, 90.3, 88.0, 73.6, 51.9.

HRMS (ESI) *m*/*z* for $C_{32}H_{24}O_2$ [M+Na]$^+$ calcd. 463.1669, found 463.1673.

1

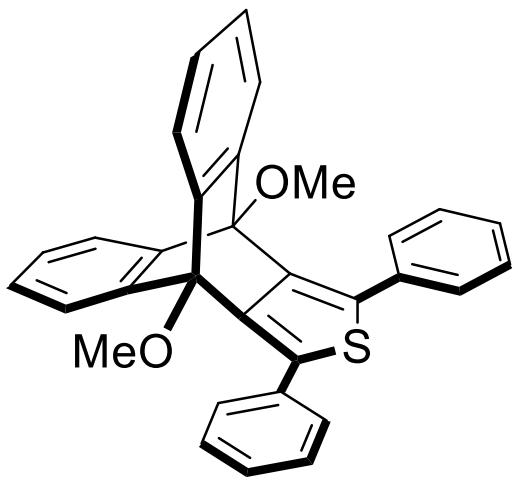

To a solution of Cp_2ZrCl_2 (175 mg, 0.60 mmol) in anhydrous THF (5 mL) maintained at −78 °C was added *n*-BuLi (0.48 mL, 1.20 mmol, 2.5 M in hexanes). After 1 hr *cis*-**4** (220 mg, 0.5 mmol) in anhydrous THF (2 mL) was added dropwise. The reaction mixture was allowed to warm to room temperature and stirred for 3 hours at this temperature. The reaction mixture was cooled back down to −78 °C and sulphur monochloride (0.08 mL, 1 mmol) was added slowly before the reaction was allowed to warm to room temperature and stirred overnight. Water (25 mL) was added and the mixture was extracted with EtOAc (25 mL). The combined organic portions were washed with saturated $NaHCO_3$ (aq.) solution, dried over $MgSO_4$, and the solvent removed under reduced pressure. The residue was purified by column chromatography on neutral alumina (20% EtOAc/hexane). After evaporation of the column solvent under reduced pressure, the resulting solid was sonicated in hexane and isolated via filtration to obtain **1** as a yellow solid (90 mg, 38%, m.p. 217 – 223 °C).

^{1}H NMR (400 MHz, chloroform-*d*, ppm) δ = 7.63 (dd, J = 5.6, 3.2 Hz, 4H), 7.55–7.49 (m, 4H), 7.43–7.34 (m, 6H), 7.14 (dd, J = 5.6, 3.2 Hz, 4H), 3.42 (s, 6H).

^{13}C NMR (101 MHz, chloroform-*d*, ppm) δ = 145.1, 140.9, 134.3, 133.3, 130.4, 128.2, 128.0, 125.5, 121.2, 86.5, 56.7.

HRMS (ASAP) *m*/*z* for $C_{32}H_{24}O_2S$ $[M+H]^+$ calcd. 473.1570, found 473.1577.

β,β-**6** and α,β-**6**

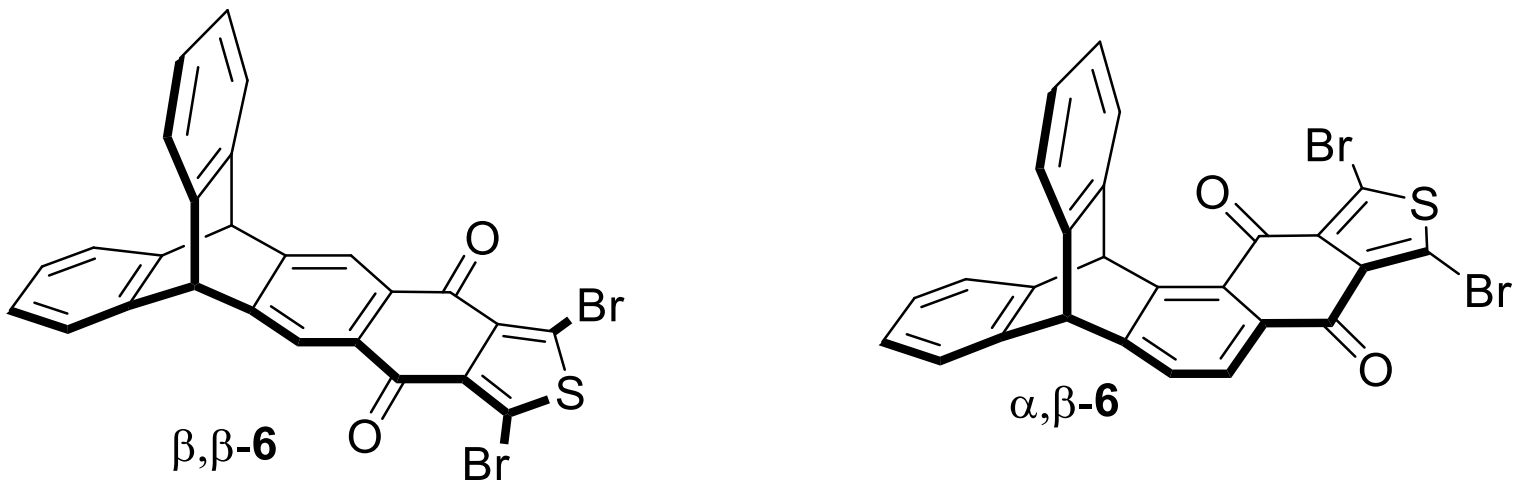

2,5-Dibromo-3,4-thiophenedicarbonyl dichloride prepared from 2,5-dibromothiophene-3,4-carboxylic acid[3] according to Chao et al[4] (11.9 mmol) then dissolved in in dry 1,2-dichloroethane (20 mL). This solution was added dropwise to a stirred suspension of $AlCl_3$ (6.97 g, 52.4 mmol) in dry 1,2-dichloroethane (40 mL), which was cooled using an ice bath. The reaction was left to stir for 10 minutes. A solution of triptycene (3.63 g, 14.3 mmol) in dry 1,2-dichloroethane (40 mL) was slowly added and the reaction left to stir at 0 °C for another 30 mins. The ice-cold reaction mixture was poured directly into 200 mL of 6 M HCl (aq.) and stirred for 10 minutes. The 1,2-dichloroethane was removed under reduced pressure and the remaining solvent decanted. The solid residue was washed with 6 M HCl (2 × 50 mL) and water (2 × 100 mL). Finally, the residue was triturated with methanol and collected via filtration. The crude solid was stirred in $CHCl_3$ and the linear quinone β,β-**6** collected via filtration as a white solid (1.85 g, 28%, m.p. >300 °C). The bent isomer α,β-**6** was obtained from the filtrate following removal of $CHCl_3$ under reduced pressure and then purification of the residue by column chromatography on SiO_2 (50% CH_2Cl_2 /hexane) followed by recrystallisation from $CHCl_3$/hexane to afford pure α,β-**6** as a yellow solid (1.05g, 16% yield, m.p. 264 – 266 °C).

β,β-**6**

^{1}H NMR (400 MHz, chloroform-*d*, ppm) δ = 8.24 (s, 2H), 7.45 (dd, J = 5.4, 3.2 Hz, 4H), 7.06 (dd, J = 5.4, 3.2 Hz, 4H), 5.65 (s, 2H).

^{13}C NMR (101 MHz, chloroform-*d*, ppm) δ = 177.6, 152.1, 143.6, 132.8, 126.1, 124.3, 122.3, 120.8, 54.3.

HRMS (ESI) *m*/*z* for $C_{26}H_{12}Br_2O_2S$ $[M+Na]^+$ calcd. 568.8817, found 568.8796.

α,β-**6**

^{1}H NMR (400 MHz, chloroform-*d*, ppm) δ 8.07 (d, J = 7.7 Hz, 1H), 7.73 (d, J = 7.7 Hz, 1H), 7.61–7.55 (m, 3H), 7.46–7.38 (m, 2H), 7.05 (dd, J = 5.6, 3.2, 4H), 5.55 (s, 1H).

^{13}C NMR (126 MHz, Chloroform-*d*, ppm) δ = 180.4, 177.4, 154.5, 149.0, 144.2, 144.1, 133.7, 132.7, 132.4, 129.1, 129.1, 126.6, 126.0, 126.0, 125.1, 124.0, 120.7, 120.3, 54.7, 49.3.

HRMS (ESI) *m*/*z* for $C_{26}H_{12}Br_2O_2S$ $[M+Na]^+$ calcd. 568.8817, found 568.8793

7

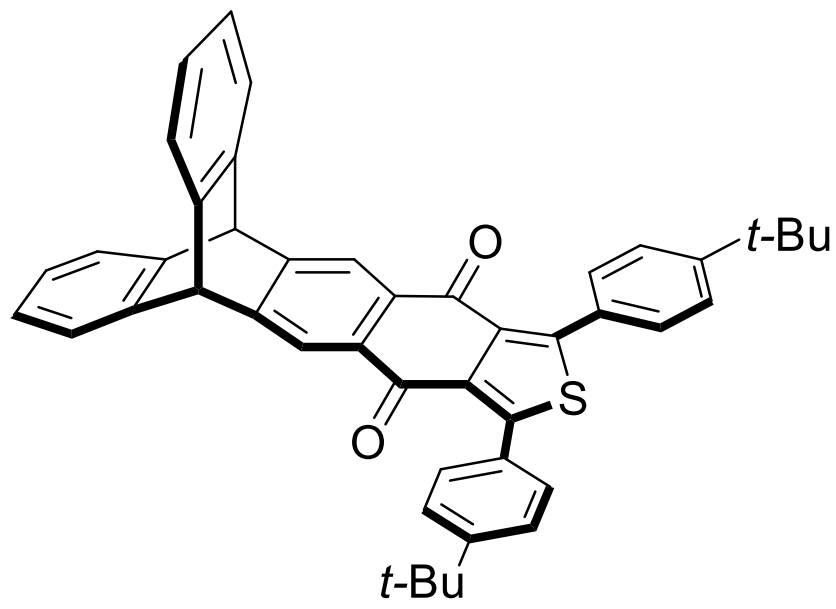

A two-neck flask fitted with a condenser was placed under an Ar atmosphere. β,β-**6** (55 mg, 0.1 mmol) and K_2CO_3 (41 mg, 0.3 mmol) were added to the flask followed by THF (12 mL) and water (4 mL). The mixture was thoroughly degassed by bubbling with Ar for 45 min. 4-tert-butylphenylboronic acid (40 mg, 0.22 mmol) was added and the mixture degassed for a further 5 min. $Pd(PPh_3)_4$ (3 mol%, 3 mg, 3 µmol) was added and the mixture heated to 55 °C for 24 hr. Water (25 mL) was added and the mixture was extracted with EtOAc. The combined organic portions were dried over $MgSO_4$, and the solvent removed under reduced pressure.

The crude product was purified by column chromatography on SiO_2 (50% CH_2Cl_2/hexane) and triturated with *i*PrOH with to yield **7** (46 mg, 71%, m.p. >300 °C) as a bright yellow solid.

^{1}H NMR (400 MHz, chloroform-*d*, ppm) *δ* 8.18 (s, 2H), 7.60 (d, *J* = 8.5 Hz, 4H), 7.49 (d, *J* = 8.5 Hz, 4H), 7.42 (dd, *J* = 5.4, 3.2 Hz, 4H), 7.04 (dd, *J* = 5.4, 3.2 Hz, 4H), 5.57 (s, 2H), 1.38 (s, 18H).

^{13}C NMR (101 MHz, chloroform-*d*, ppm) *δ* =180.0, 152.9, 152.1, 151.2, 143.8, 133.5, 130.8, 129.6, 129.4, 125.9, 125.4, 124.2, 122.1, 54.3, 35.0, 31.4.

HRMS (MALDI) *m*/*z* for $C_{46}H_{38}O_2S$ $[M+H]^+$ calcd. 655.2665, found 655.2663.

8

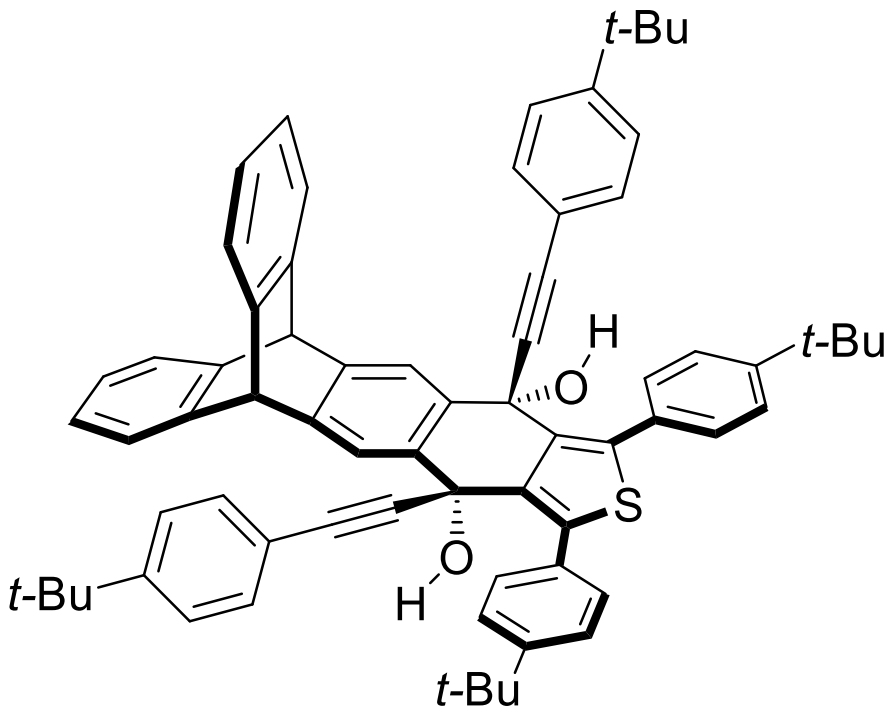

n-BuLi (0.37 mL, 2.5M in hexanes, 0.916 mmol) was added dropwise to a solution to 4-*tert*-butylphenylacetylene (0.18 mL, 0.977 mmol) in toluene (5 mL) at 0 °C. The mixture was warmed to room temperatures and stirred for 45 min. **7** (200 mg, 0.305 mmol) was added as a solid in one portion, and the resulting mixture was heated to 80 °C and stirred at this temperature overnight. The reaction mixture was quenched with saturated NH_4Cl (aq.) (25 mL) at 0 °C, then diluted with CH_2Cl_2 (20 mL) and water (75 mL). The organic phase was separated, and the organic phase extracted once more with CH_2Cl_2 (20 mL). The combined organics were washed with water (100 mL), dried over $MgSO_4$, and the solvent removed under reduced pressure. The residue was purified by chromatography on SiO_2 (10% EtOAc/hexane), yielding pure *cis*-diol **8** as a waxy yellow solid (150 mg, 51%) which was used immediately in the next step.

^{1}H NMR (400 MHz, chloroform-*d*, ppm) δ = 8.07 (s, 2H), 7.67 (d, J = 8.3 Hz, 4H), 7.40–7.35 (m, 6H), 7.35–7.31 (m, 2H), 7.18 (d, J = 8.3 Hz, 4H), 7.07 (d, J = 8.3 Hz, 4H), 7.04–6.93 (m, 4H), 5.45 (s, 2H), 3.47 (s, 2H), 1.29 (s, 18H), 1.24 (s, 18H).

^{13}C NMR (101 MHz, chloroform-*d*, ppm) δ = 151.5, 151.1, 146.0, 145.0, 144.9, 140.2, 135.4, 134.3, 131.6, 131.5, 130.3, 125.3, 125.3, 125.0, 123.9, 123.8, 122.2, 119.6, 90.1, 88.1, 67.8, 54.0, 34.8, 34.7, 31.3, 31.2.

HRMS (MALDI) *m*/*z* for $C_{70}H_{67}O_2S$ $[M]^+$ calcd. 971.4856, found 971.4855.

9

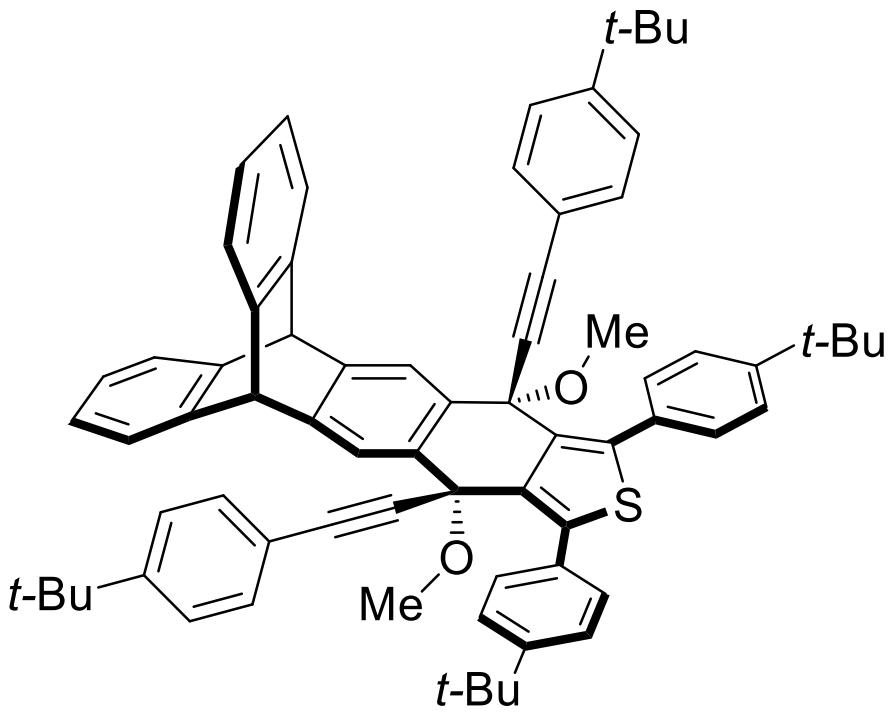

8 (150 mg, 0.154 mmol) in anhydrous THF (1 mL) was added to a stirred suspension of NaH (60 wt% in paraffin, 15 mg, 0.39 mmol) in anhydrous THF (0.5 mL) at −78 °C. The suspension was stirred for 30 min at the same temperature. Methyl iodide (0.05 mL, 0.77 mmol) was added dropwise slowly with a syringe and the mixture was allowed to warm to room temperature overnight (18 h). The reaction mixture was poured into ice-cold saturated NH_4Cl (aq.) (20 mL). The organic layer was separated, and the aqueous phase was repeatedly extracted with Et_2O (3 × 25 mL). The combined organic extracts were washed with water (2 × 50 mL) and finally with saturated brine solution (50 mL), dried over $MgSO_4$, and the solvent removed under reduced pressure. The crude **9** obtained (145 mg, 94%, m.p. 205 – 208 °C) was used without further purification in the next step.

^{1}H NMR (500 MHz, chloroform-*d*, ppm) δ = 7.98 (s, 2H), 7.74–7.70 (m, 4H), 7.43–7.40 (m, 2H), 7.39–7.34 (m, 6H), 7.20–7.15 (m, 4H), 7.06 – 7.02 (m, 2H), 7.01 – 6.95 (m, 6H), 5.45 (s, 2H), 3.08 (s, 6H), 1.28 (s, 18H), 1.27 (s, 18H).

^{13}C NMR (126 MHz, chloroform-*d*, ppm) δ = 151.3, 150.5, 145.3, 145.2, 145.1, 141.9, 133.0, 132.2, 132.2, 131.5, 130.4, 125.4, 125.3, 124.9, 124.7, 124.0, 123.9, 119.8, 89.4, 88.7, 72.6, 54.0, 51.5, 34.8, 34.7, 31.4, 31

HRMS (LDI) *m/z* for $C_{72}H_{70}O_2S$ $[M+H]^+$ calcd. 999.5169, found 999.5226.

2

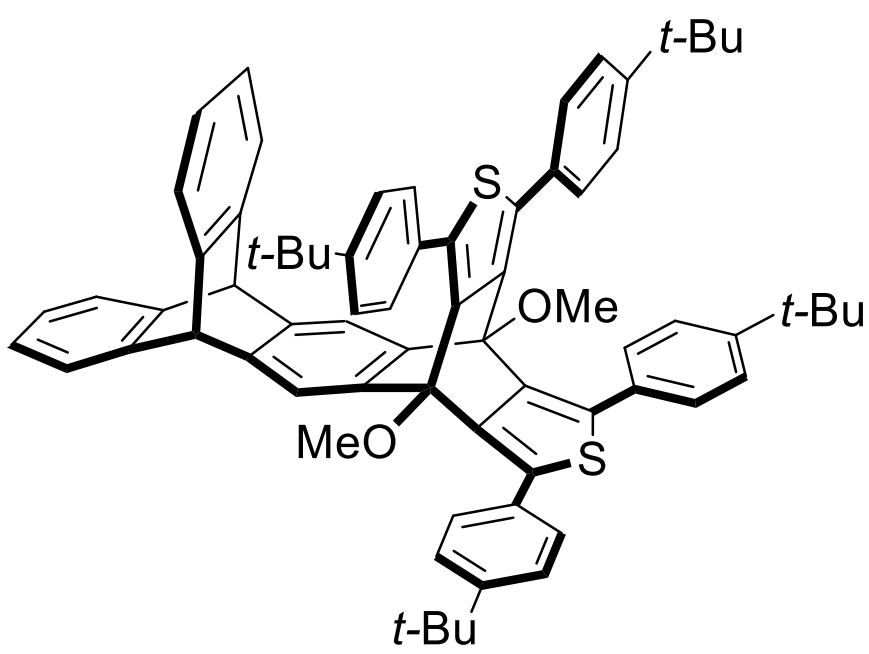

To a solution of Cp_2ZrCl_2 (51 mg, 0.174 mmol) in anhydrous THF (3 mL) maintained at −78 °C was added *n*-BuLi (0.14 mL, 0.348 mmol, 2.5 M in hexanes). After 1 hr **123** (145 mg, 0.145 mmol) in anhydrous THF (2 mL) was added dropwise. The reaction mixture was allowed to warm to r.t. and stirred for 3 hr at this temperature. The reaction mixture was cooled to −78 °C and sulphur monochloride (0.02 mL, 0.29 mmol) was added slowly then warmed to room temperature and stirred overnight. Water (25 mL) was added and the mixture was extracted with EtOAc (3 × 20 mL). The combined organic portions were washed with saturated $NaHCO_3$ (aq.), dried over $MgSO_4$, and the solvent removed under reduced pressure. The residue was purified by column chromatography on neutral alumina (5% EtOAc/hexane). After evaporation of the column solvent under reduced pressure, the resulting solid was sonicated in methanol and isolated via filtration to obtain **2** as a beige solid (25 mg, 17%, m.p. 70 – 73 °C). Crystals were obtained from CH_2Cl_2/methanol.

^{1}H NMR (400 MHz, chloroform-*d*, ppm) δ = 7.72 (s, 2H), 7.41 (dd, *J* = 5.4, 3.2 Hz, 4H), 7.33 (d, *J* = 8.4 Hz, 8H), 7.26 (d, *J* = 8.4 Hz, 8H), 7.00 (dd, *J* = 5.4, 3.2 Hz, 4H), 5.47 (s, 2H), 2.61 (s, 6H), 1.32 (m, 36H).

^{13}C NMR (101 MHz, chloroform-*d*, ppm) δ = 150.7, 145.6, 143.0, 142.6, 140.9, 133.1, 131.4, 130.1, 125.4, 124.6, 123.8, 117.3, 87.2, 55.6, 54.4, 34.7, 31.5.

HRMS (LDI) *m*/*z* for $C_{72}H_{70}O_2S_2$ $[M+H]^+$ calcd. 1031.4890, found 1031.4833.

trans-**10** and *cis*-**10**

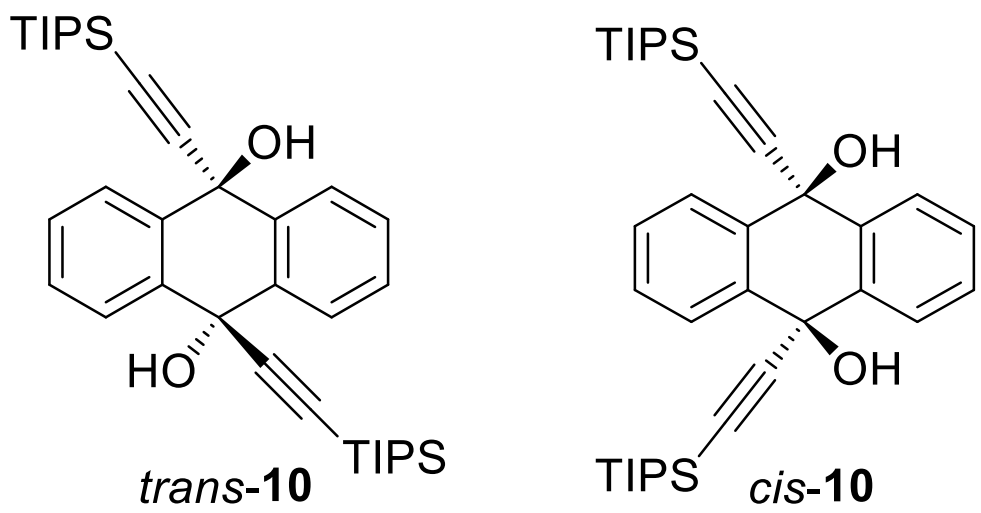

trans-**10** *cis*-**10**

n-BuLi (4.69 mL, 1.6M in hexanes, 7.50 mmol) was added dropwise to a solution to (triisopropylsilyl)acetylene (1.96 mL, 8.75 mmol) in toluene (22 mL) at 0 °C. The mixture was warmed to 23 °C and stirred for 45 minutes. 9,10-Anthraquinone (520 mg, 2.50 mmol) was added as a solid in one portion, and the resulting mixture was heated to 55 °C and stirred overnight. The reaction mixture was quenched with saturated NH_4Cl (aq.) (100 mL) at 0 °C, then diluted with Et_2O (50 mL) and water (50 mL). The organic phase was separated, and the aqueous phase extracted once more with Et_2O (50 mL). The combined organic extracts were washed with water (150 mL) and brine (100 mL), then dried over $MgSO_4$, and the solvent removed under reduced pressure. The residue was purified by column chromatography on SiO_2 (10% EtOAc/hexane), first eluting *trans*-**10** (869 mg, 60%, m.p. 145 – 149 °C) then *cis*-**10** as pale-yellow solids (420 mg, 29%, m.p. 183 – 189 °C).

trans-**10**

^{1}H NMR (400 MHz, chloroform-*d*, ppm) δ 8.25 (dd, *J* = 5.7, 3.3 Hz, 4H), 7.45 (dd, *J* = 5.9, 3.4 Hz, 4H), 3.88 (s, 2H), 1.24 – 1.18 (m, 42H).

^{13}C NMR (101 MHz, , chloroform-*d*, ppm) δ 138.7, 129.1, 127.6, 105.7, 92.1, 71.4, 18.9, 11.5.

HRMS (MALDI) *m*/*z* for $C_{36}H_{52}O_2Si_2$ [M+Na$^+$] calcd. 595.3398, found 595.3397.

cis-**10**

^{1}H NMR (400 MHz, chloroform-*d*, ppm) *δ* = 8.23 (dd, *J* = 5.7, 3.4 Hz, 4H), 7.44 (dd, *J* = 5.7, 3.4 Hz, 4H), 3.89 (s, 2H), 1.21 – 1.17 (m, 42H).

^{13}C NMR (101 MHz, chloroform-*d*, ppm) *δ* = 138.7, 129.1, 127.6, 105.8, 92.0, 71.4, 18.9, 11.5.

HRMS (MALDI) *m*/*z* for $C_{36}H_{52}O_2Si_2$ [M+Na$^+$] calcd. 595.3398, found 595.3403.

cis-**11**

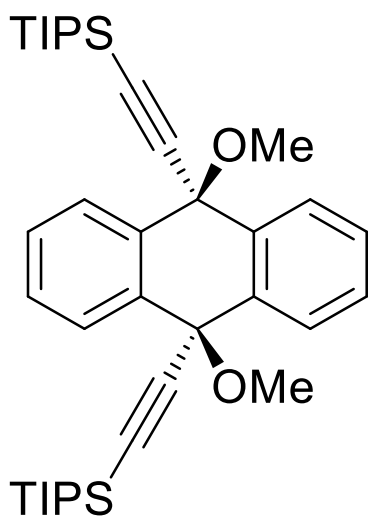

cis-**10** (350 mg, 0.61 mmol) in anhydrous THF (5 mL) was added to a stirred suspension of NaH (60 wt% in paraffin, 61 mg, 1.53 mmol) in anhydrous THF (5 mL) at −78 °C. The suspension was stirred for 30 min at the same temperature. Methyl iodide (0.19 mL, 3.05 mmol) was added dropwise slowly with a syringe and the mixture was allowed to warm to r.t. overnight. The reaction mixture was poured into ice-cold saturated NH_4Cl (aq.) (20 mL). The organic layer was separated, and the aqueous layer was extracted with Et_2O (3 × 25 mL). The combined organic extract was washed with water (2 × 50 mL) and finally with saturated brine solution (50 mL), dried over $MgSO_4$, and the solvent removed under reduced pressure. The residue was purified by column chromatography on SiO_2 (5% EtOAc/hexanes), yielding *cis*-**11** as a yellow solid (262 mg, 71%, m.p. 162 – 165 °C.).

^{1}H NMR (400 MHz, chloroform-*d*, ppm) δ = 8.15 (dd, J = 5.8, 3.4 Hz, 4H), 7.43 (dd, J = 5.8, 3.4 Hz, 4H), 3.16 (s, 6H), 1.17 – 1.11 (m, 42H).

^{13}C NMR (126 MHz, chloroform-*d*, ppm) δ = 136.3, 129.5, 128.5, 106.1, 91.4, 75.5, 52.3, 18.9, 11.5.

HRMS (MALDI) *m*/*z* for $C_{38}H_{56}O_2Si_2$ [M+Na$^+$] calcd. 623.3711, found 623.3704.

12

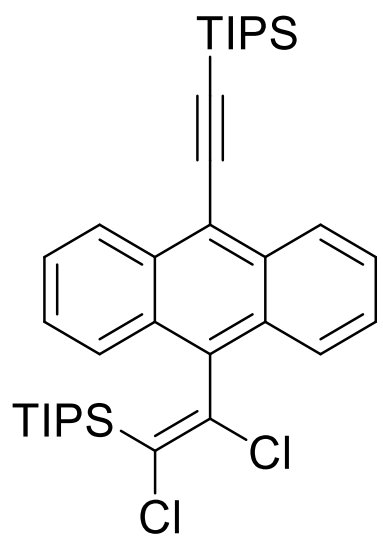

To a solution of Cp_2ZrCl_2 (643 mg, 2.20 mmol) in anhydrous THF (20 mL) maintained at −78 °C was added *n*-BuLi (1.76 mL, 4.39 mmol, 2.5 M in hexanes). After 1 hr *cis*-**11** (1.10 g, 1.83 mmol) in anhydrous THF (20 mL) was added dropwise. The reaction mixture was allowed to warm to r.t. and stirred for 3 hr at this temperature. The reaction mixture was cooled to −78 °C and S_2Cl_2 (0.29 mL, 3.66 mmol) was added slowly. The reaction was allowed to warm to r.t. and stirred overnight. Water (50 mL) was added and the mixture was extracted with EtOAc (3 × 50 mL). The combined organic extracts were washed with saturated $NaHCO_3$ (aq.) (100 mL), dried over $MgSO_4$, and the solvent removed under reduced pressure. The residue was purified by column chromatography on silica gel (100% hexane). After evaporation of the column solvent under reduced pressure, the resulting solid was recrystallised from *i*PrOH and isolated via filtration to obtain **12** as a bright yellow crystalline solid (464 mg, 35%, m.p. 144 – 148 °C).

^{1}H NMR (400 MHz, chloroform-*d*, ppm) δ = 8.68–8.60 (m, 2H), 8.21–8.14 (m, 2H), 7.63–7.54 (m, 4H), 1.31–1.24 (m, 21H), 0.76–0.64 (m, 21H).

^{13}C NMR (101 MHz, chloroform-*d*, ppm) δ = 138.9, 136.7, 132.7, 132.1, 129.7, 127.3, 126.9, 126.9, 126.6 120.4, 104.9, 103.2, 19.0, 18.7, 12.0, 11.7.

HRMS (ASAP) *m*/*z* for $C_{36}H_{50}Cl_2Si_2$ $[M+H]^+$ calcd. 609.2901, found 609.2912.

^{1}H and ^{13}C NMR Spectra

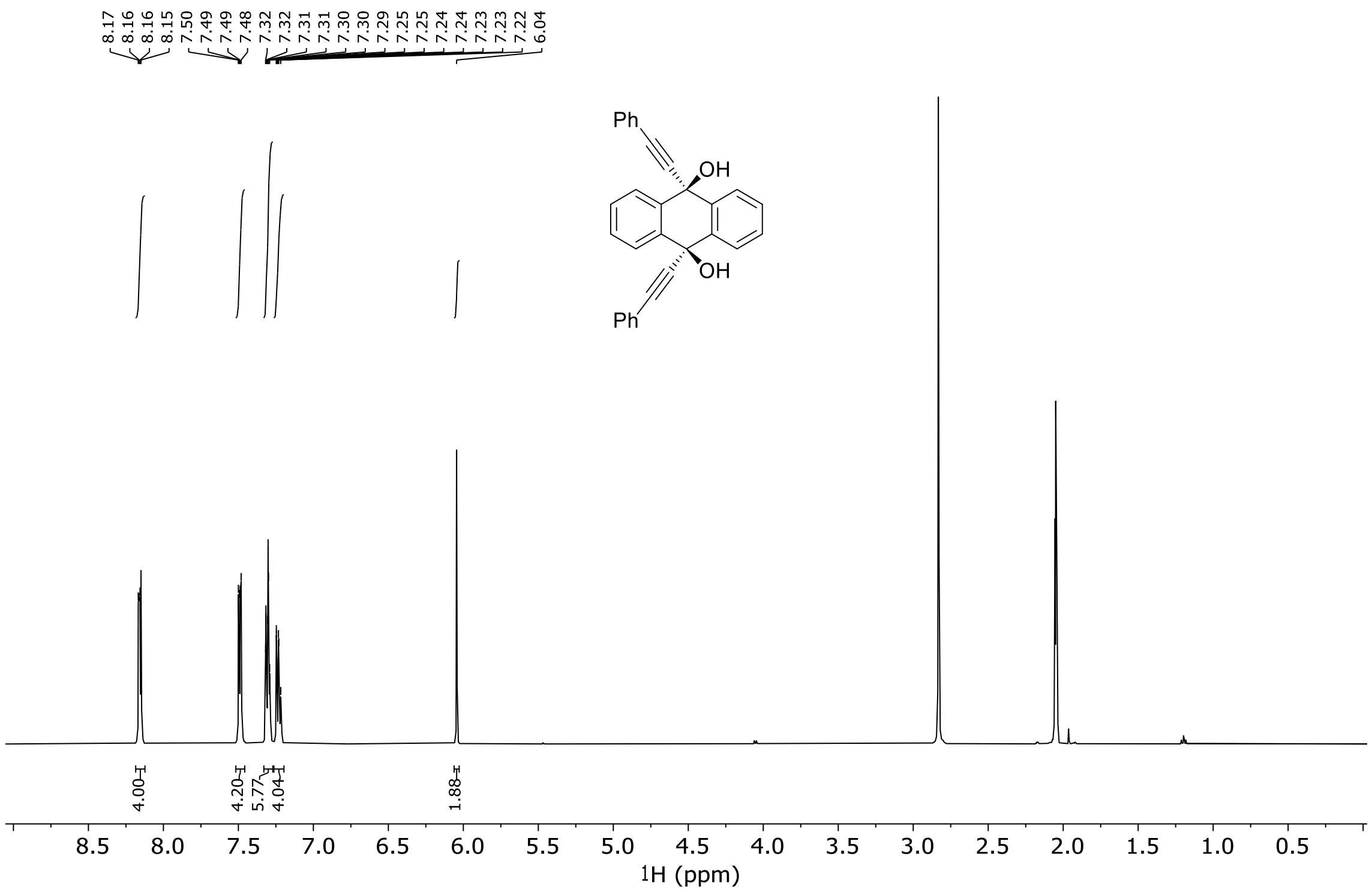

Spectrum S1: ^{1}H NMR spectrum for compound *cis*-**3**

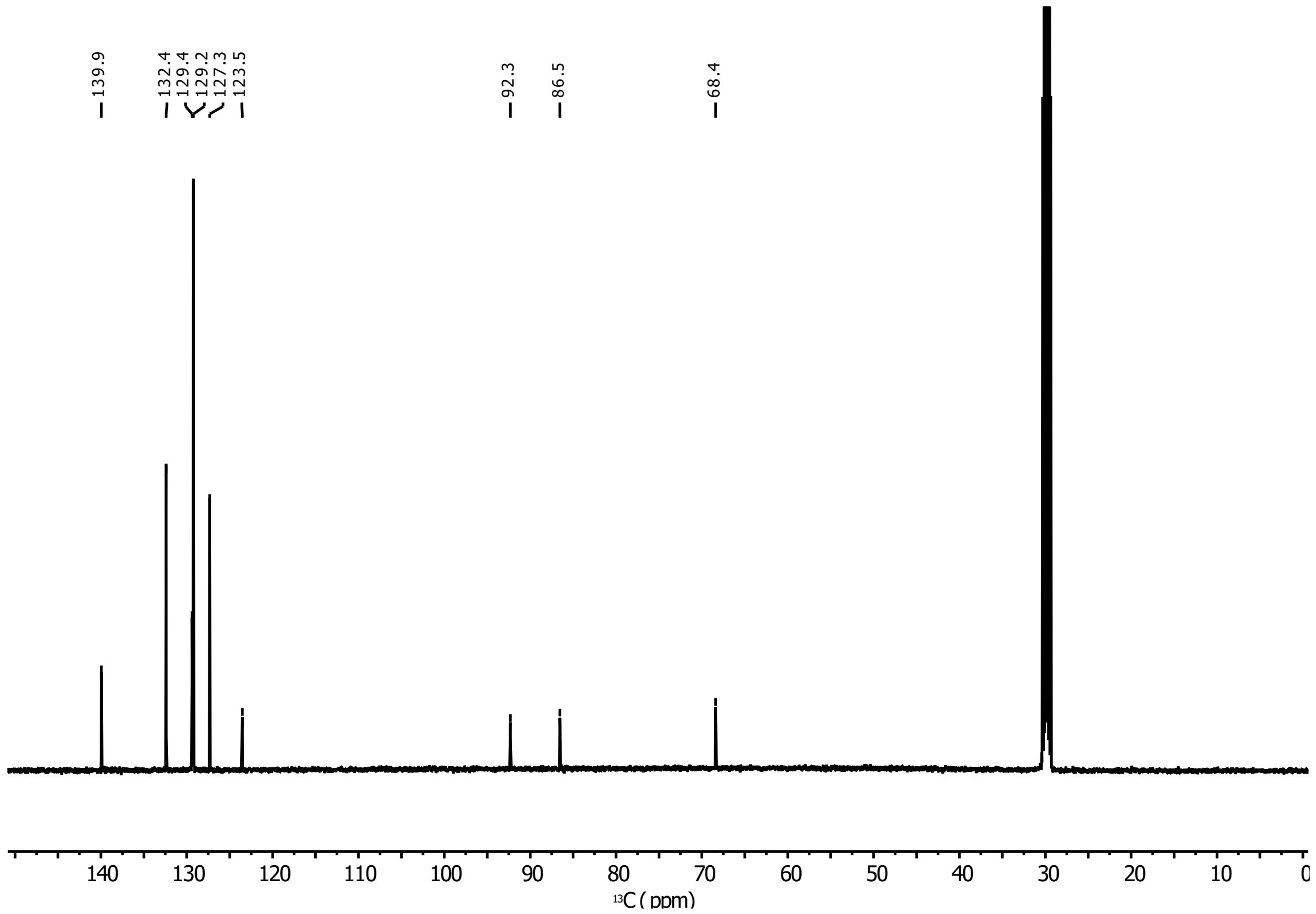

Spectrum S2: ^{13}C NMR spectrum for compound *cis*-**3**

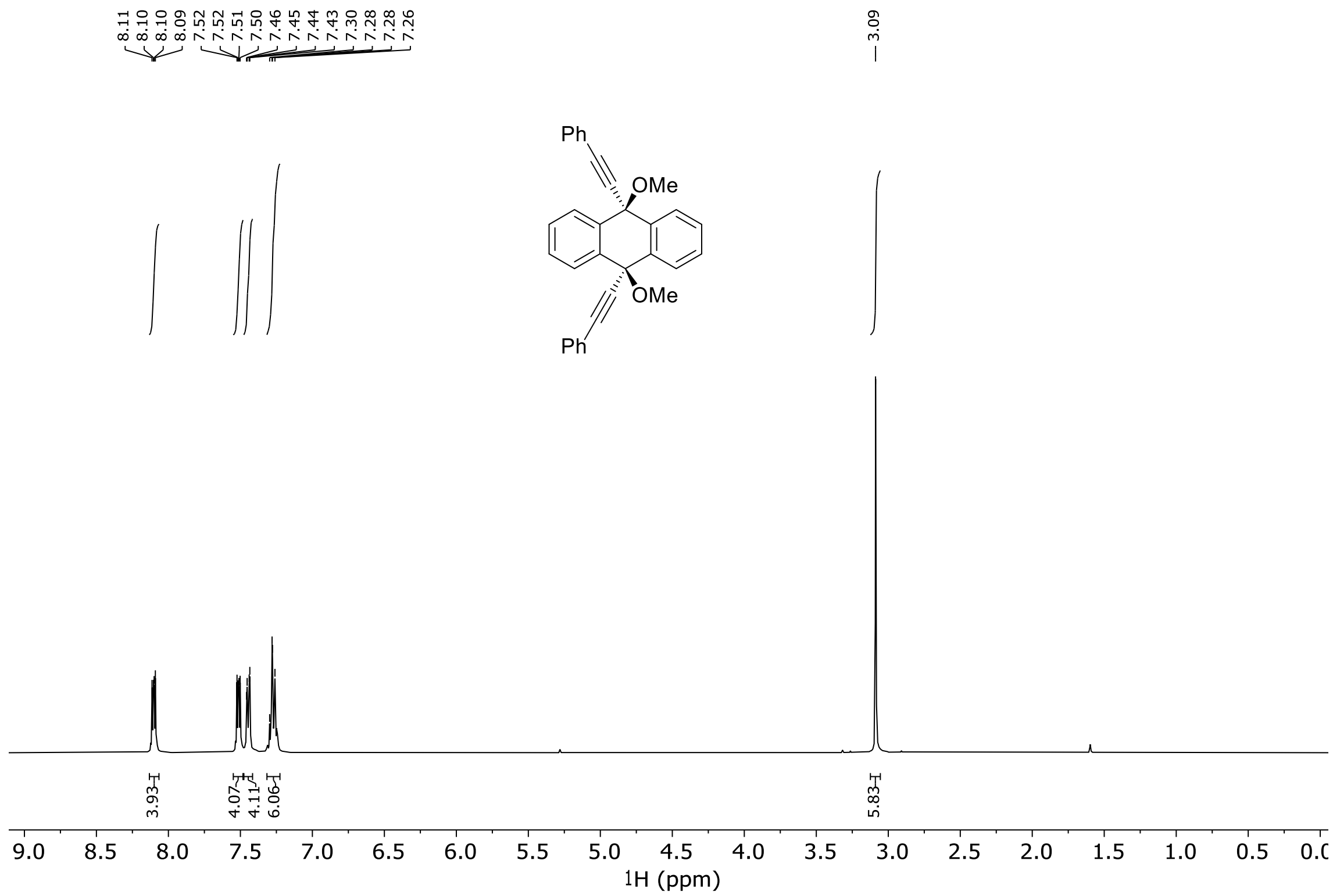

Spectrum S3: ^{1}H NMR spectrum for compound *cis*-**4**

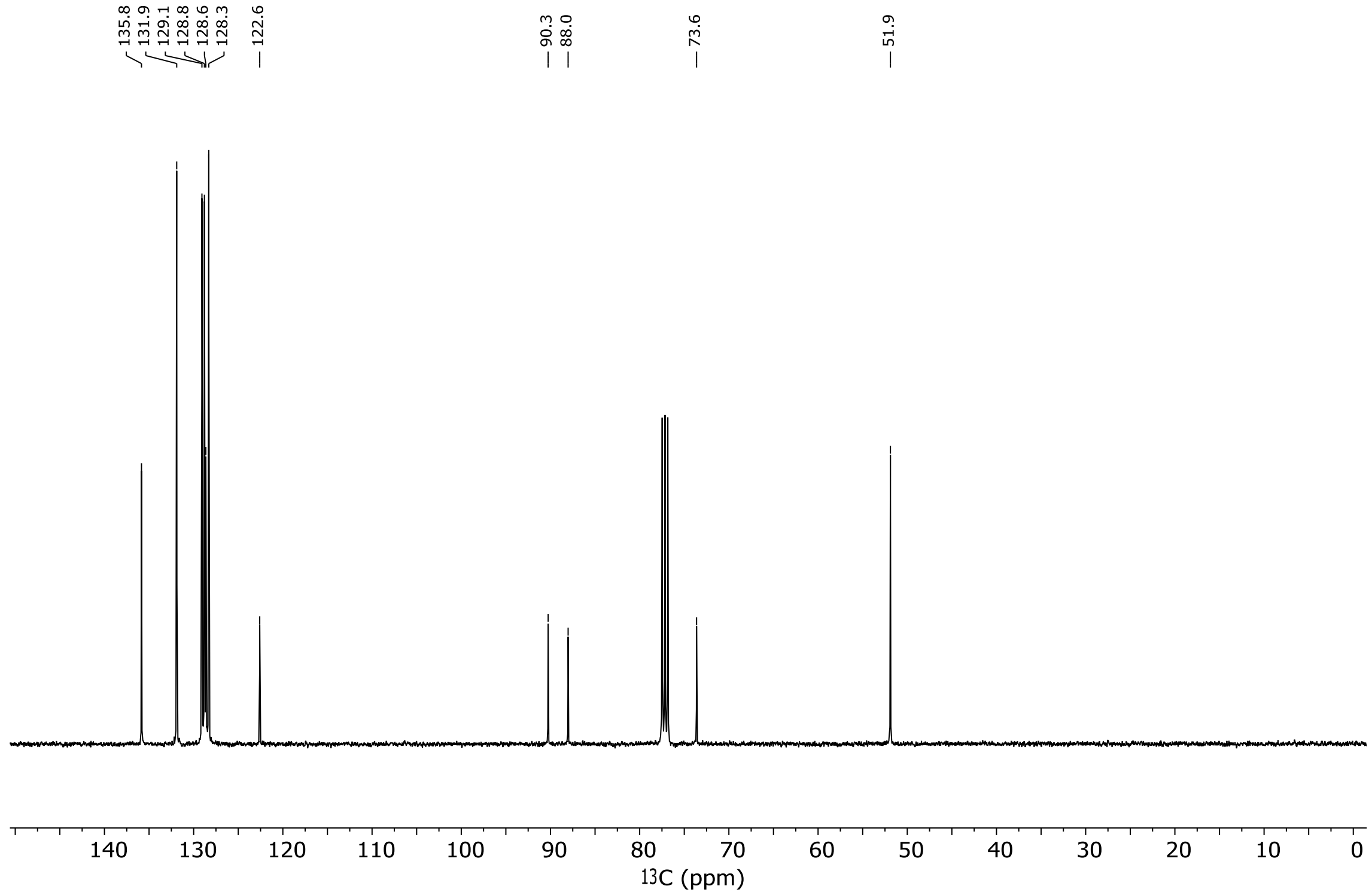

Spectrum S4: ^{13}C NMR spectrum for compound *cis*-**4**

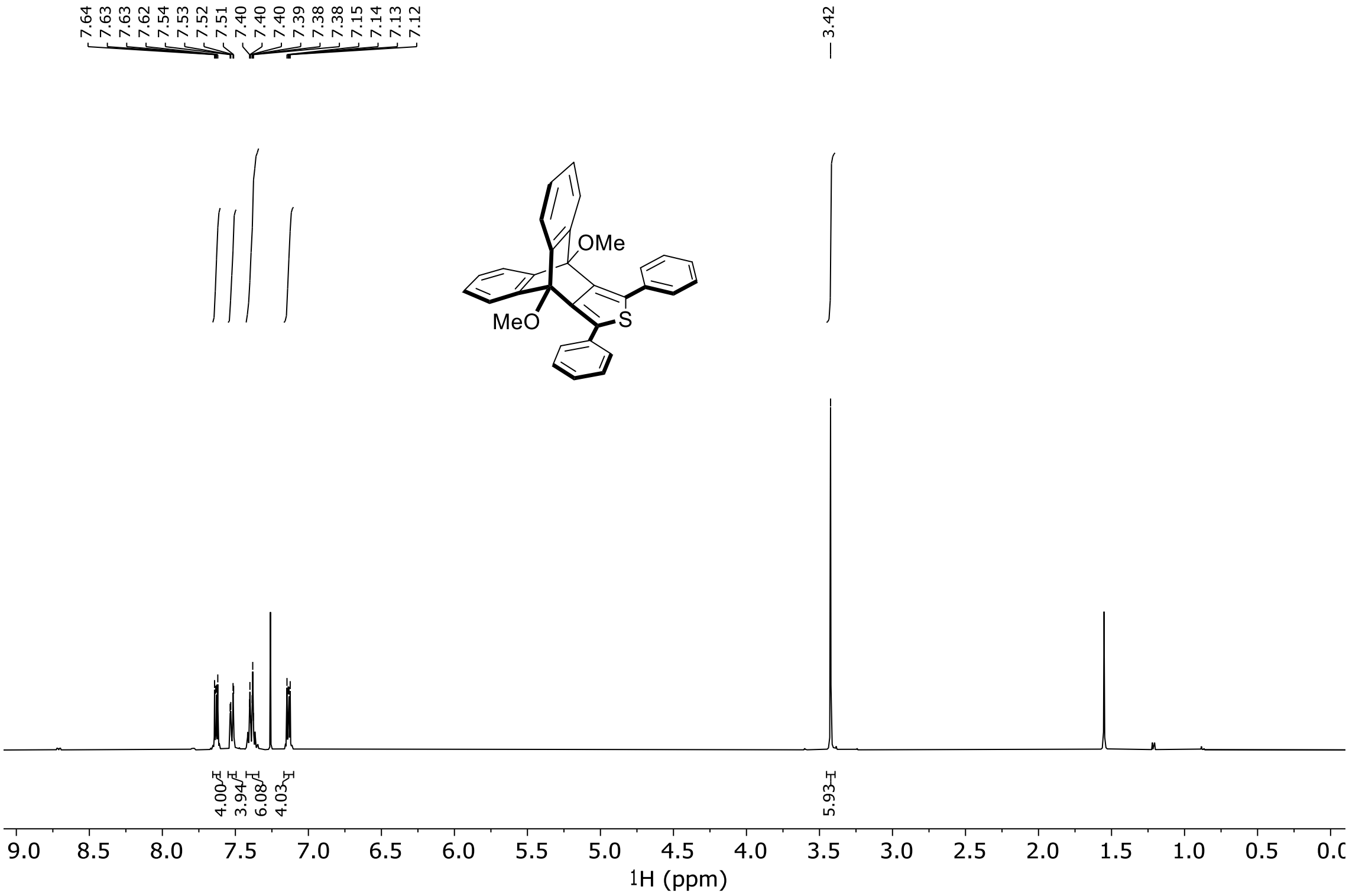

Spectrum S5: ^{1}H NMR spectrum for compound **1**.

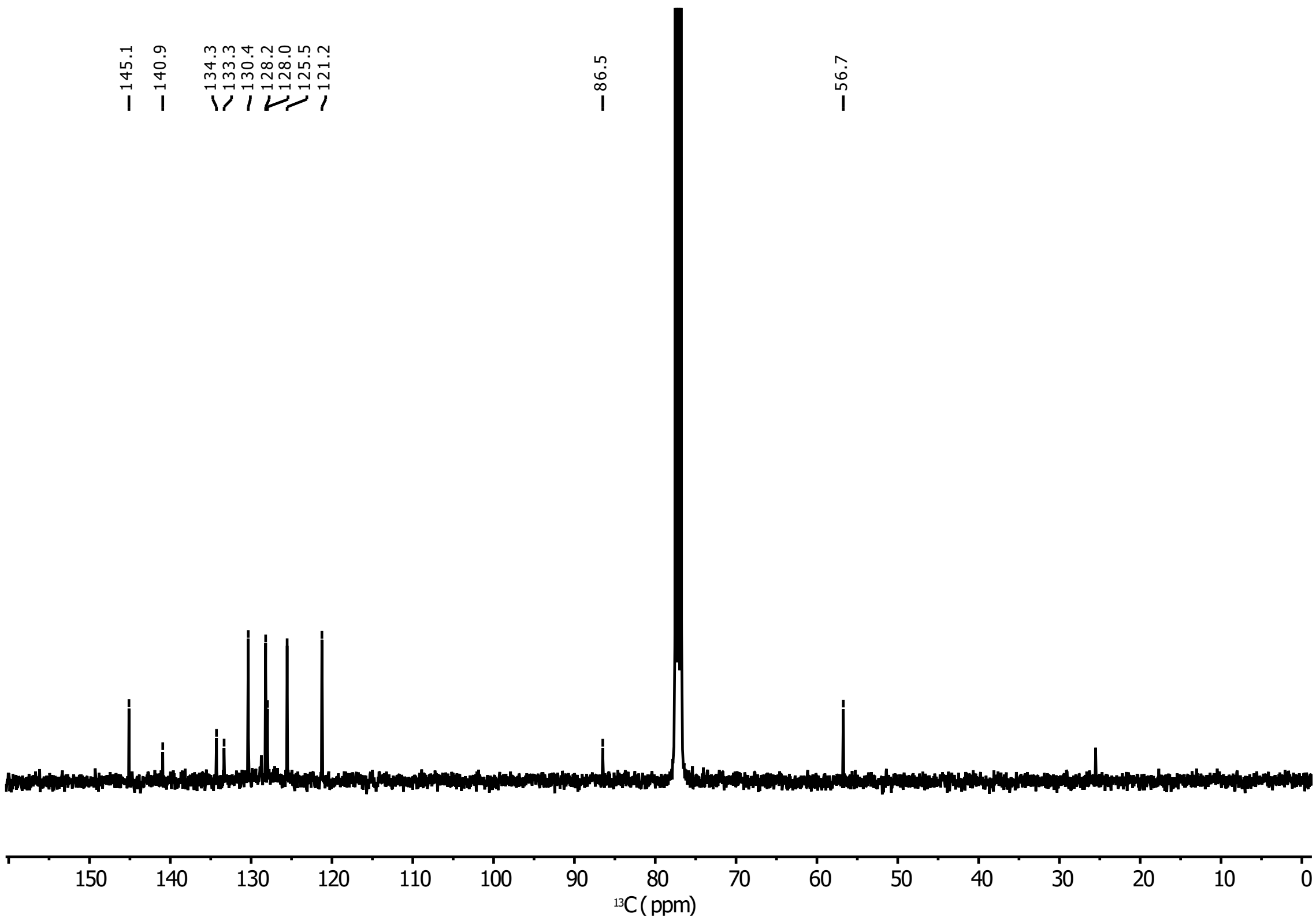

Spectrum S6: ^{13}C NMR spectrum for compound **1**.

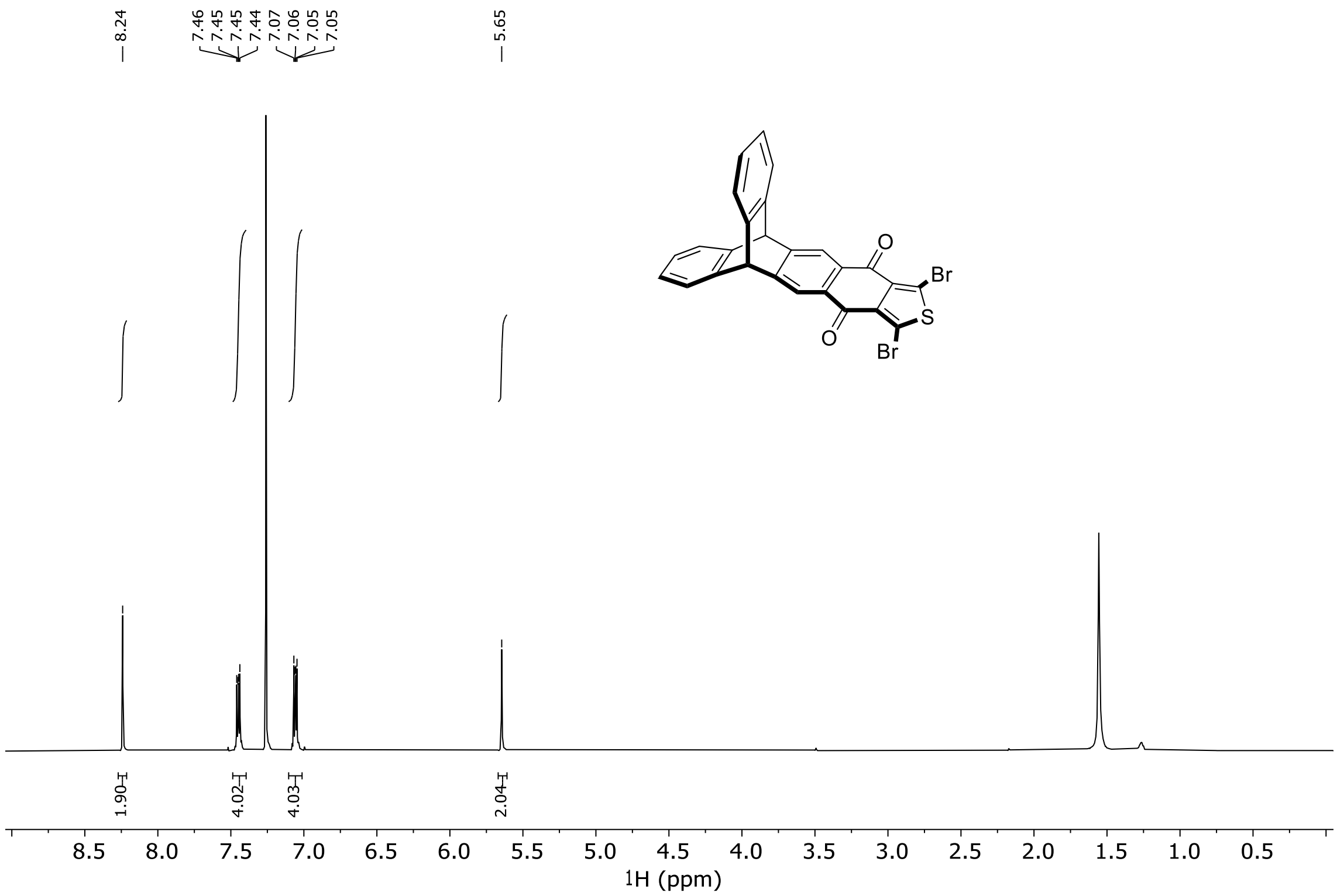

Spectrum S7: ^{1}H NMR spectrum for compound β,β-**6**.

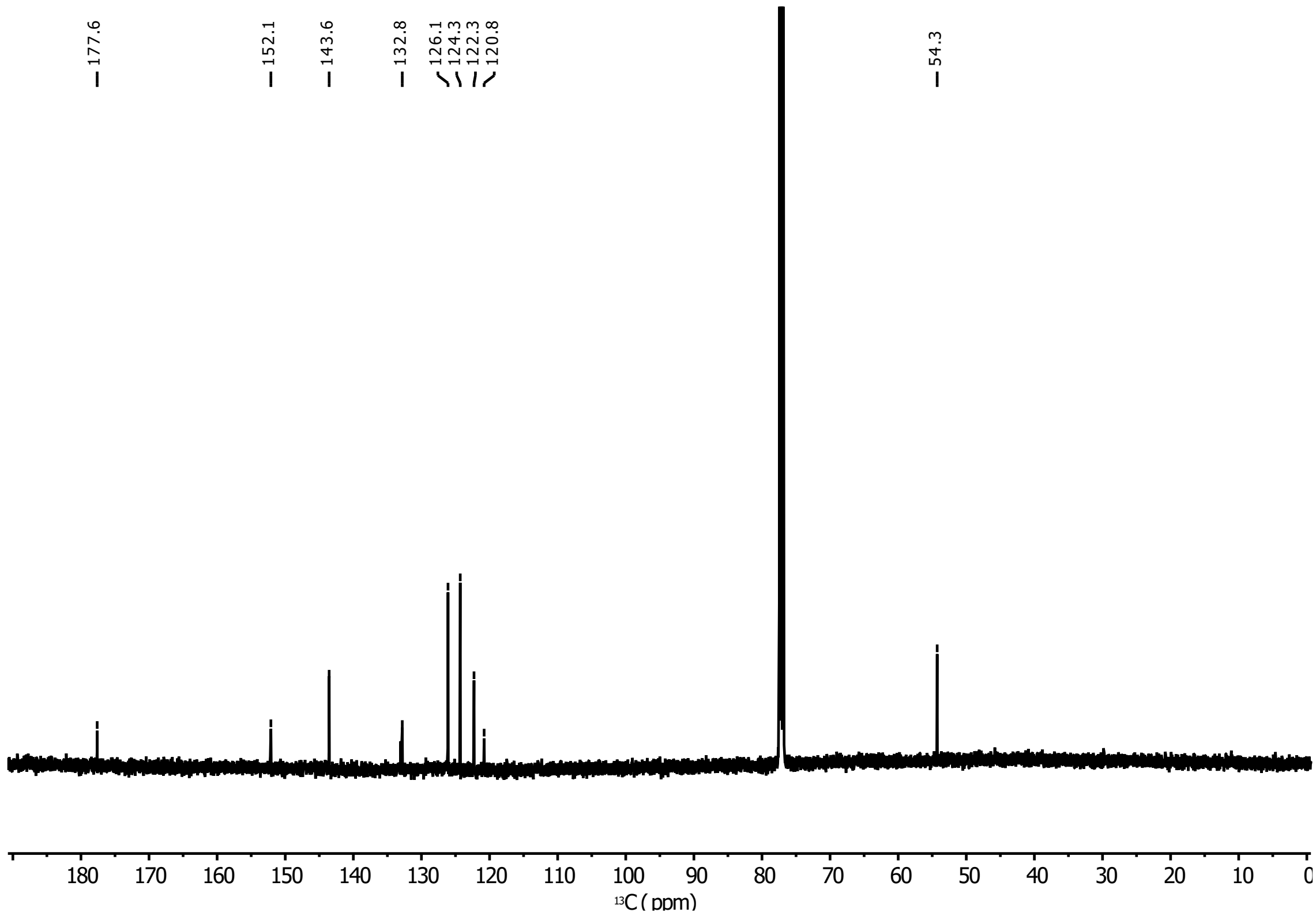

Spectrum S8: ^{13}C NMR spectrum for compound β,β-**6**.

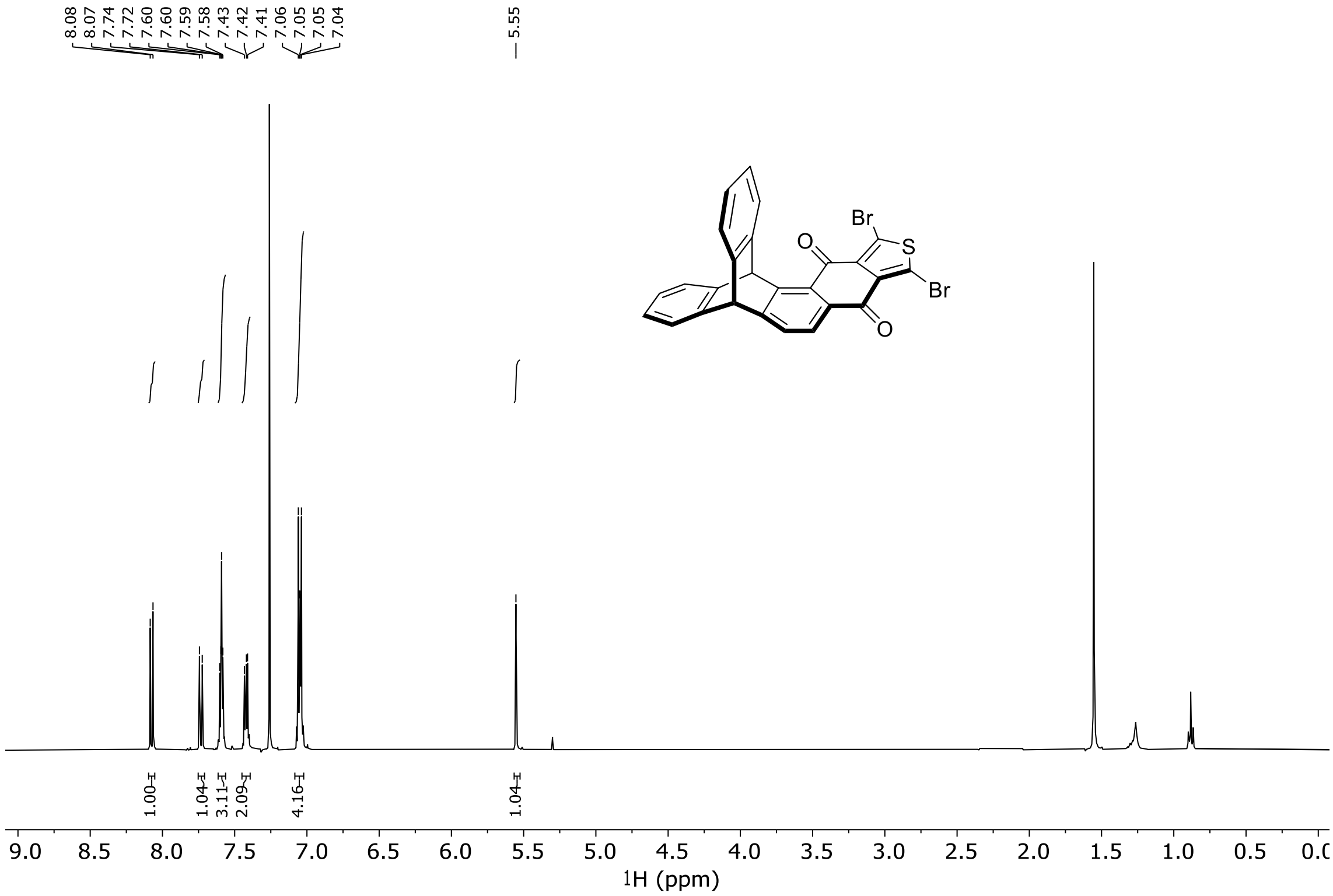

Spectrum S9: ^{1}H NMR spectrum for compound α,β-**6**.

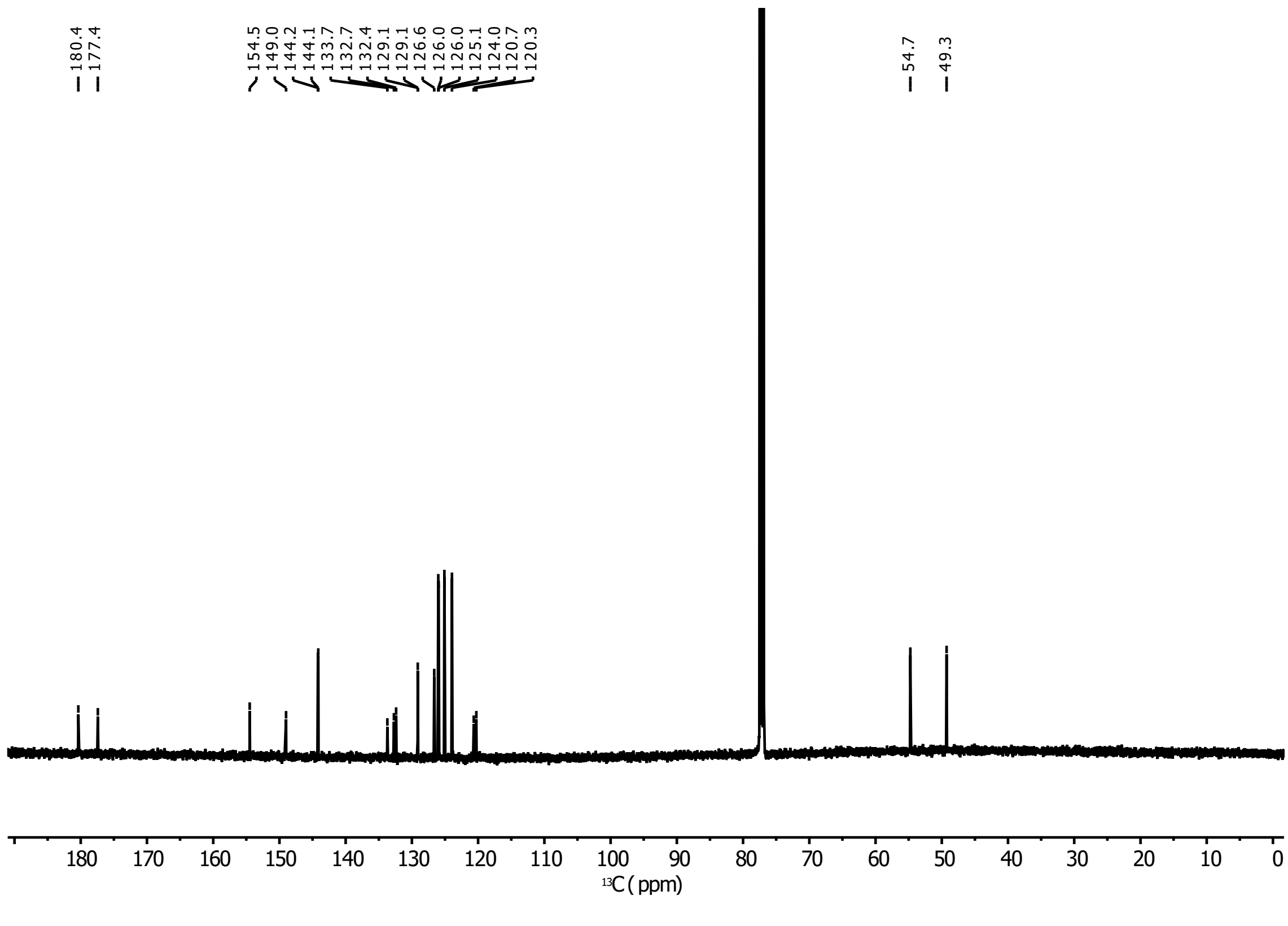

Spectrum S10: ^{13}C NMR spectrum for compound α,β-**6**.

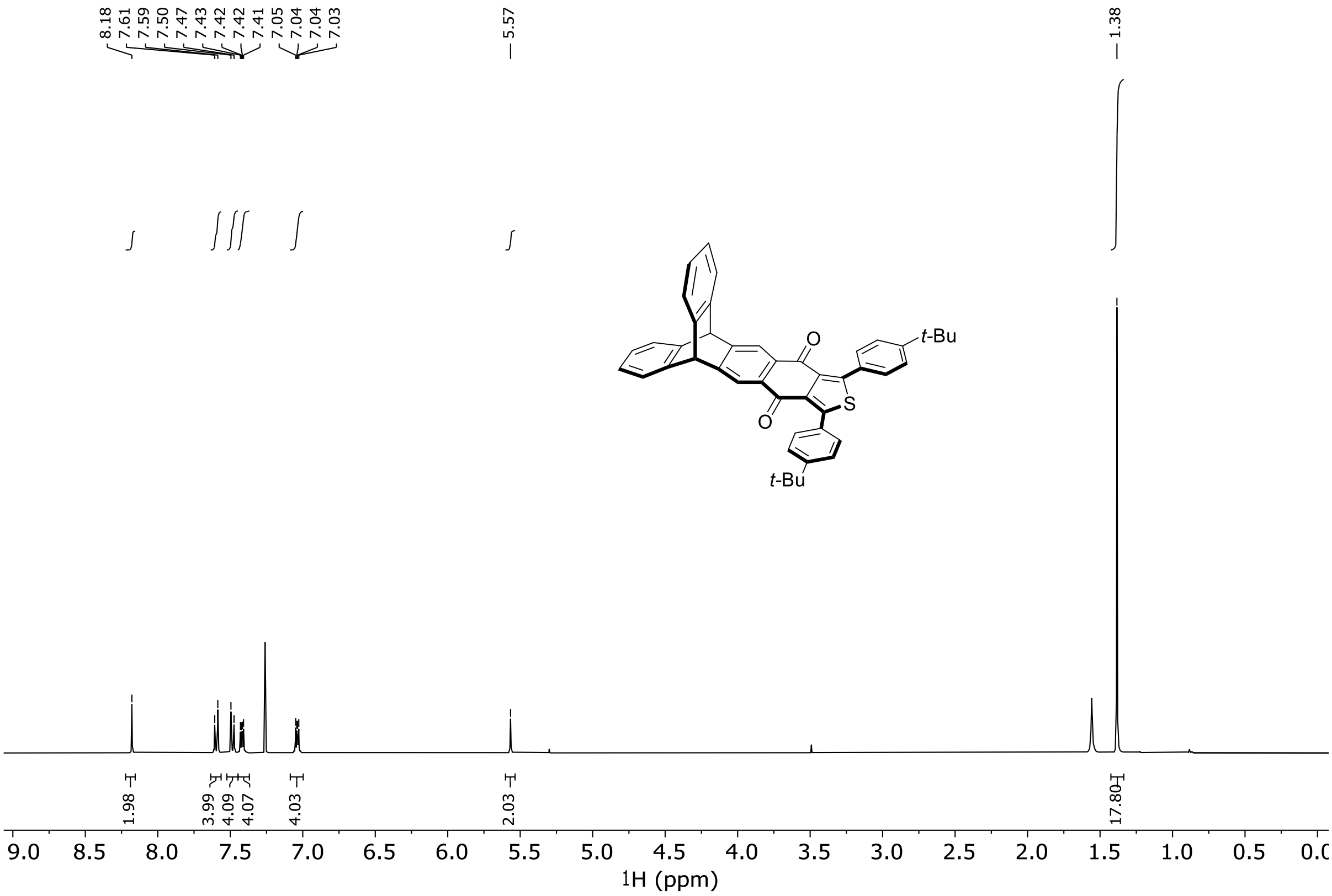

Spectrum S11: ^{1}H NMR spectrum for compound **7**.

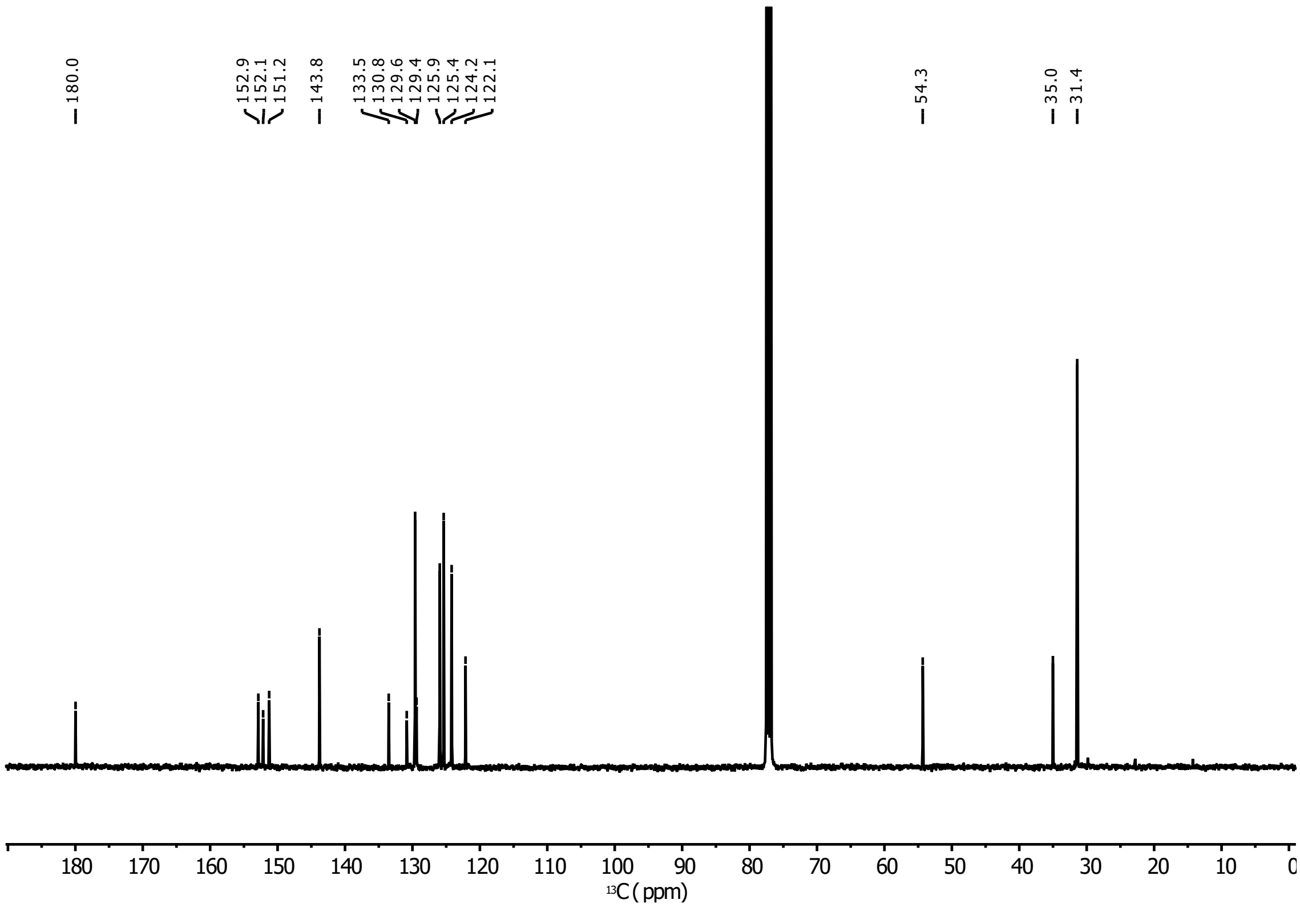

Spectrum S12: ^{13}C NMR spectrum for compound **7**.

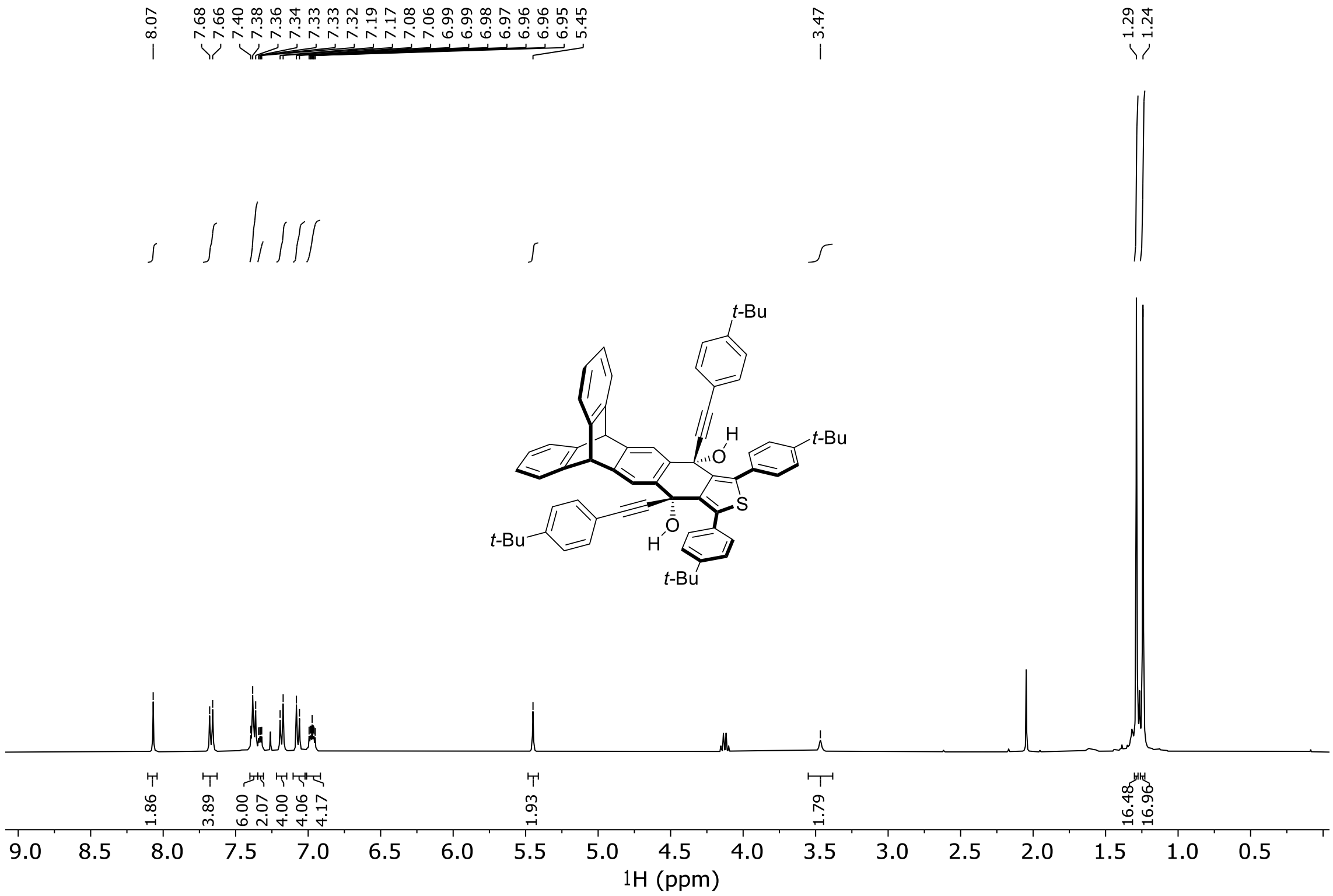

Spectrum S13: ^{1}H NMR spectrum for compound **8**.

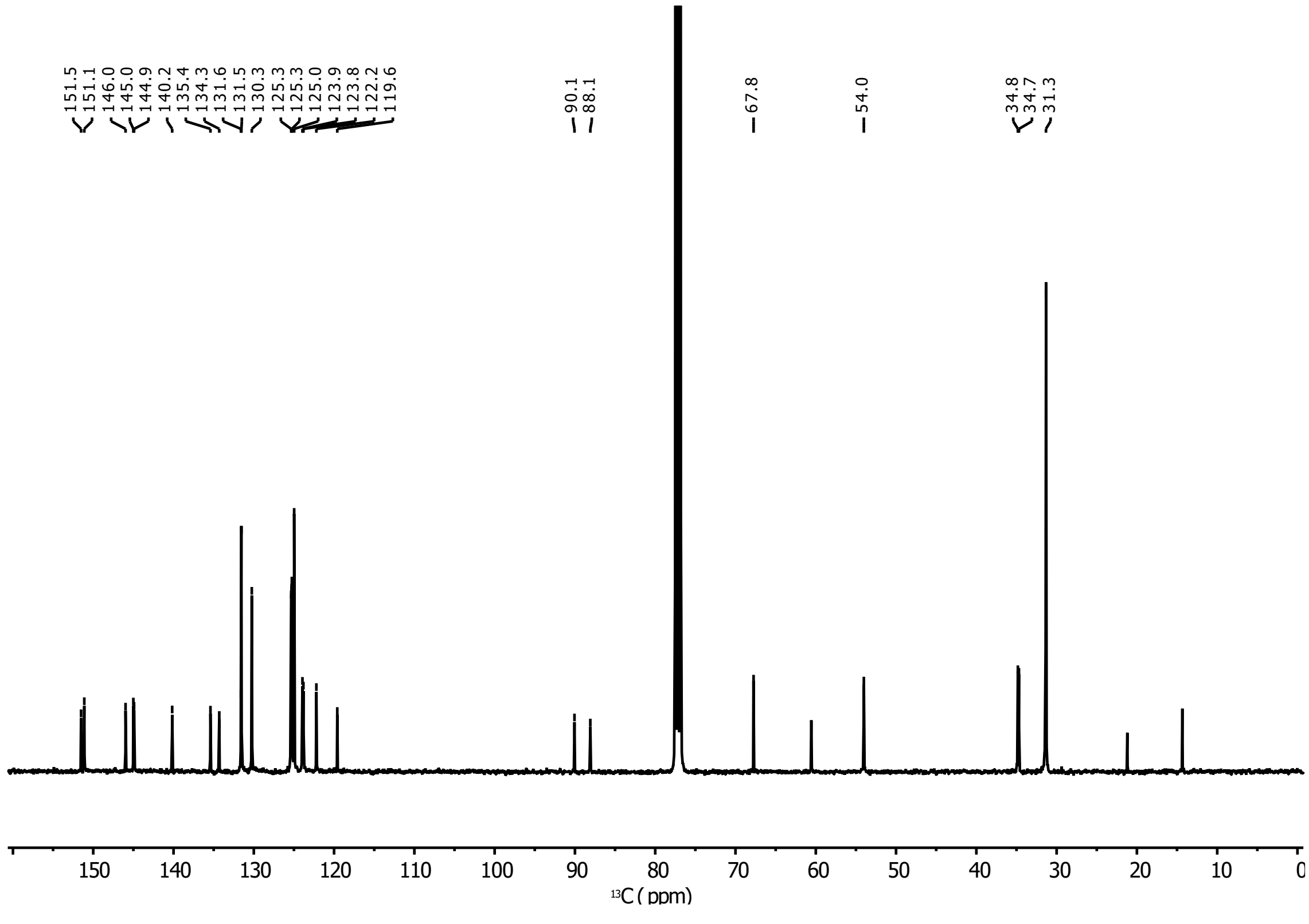

Spectrum S14: ^{13}C NMR spectrum for compound **8**.

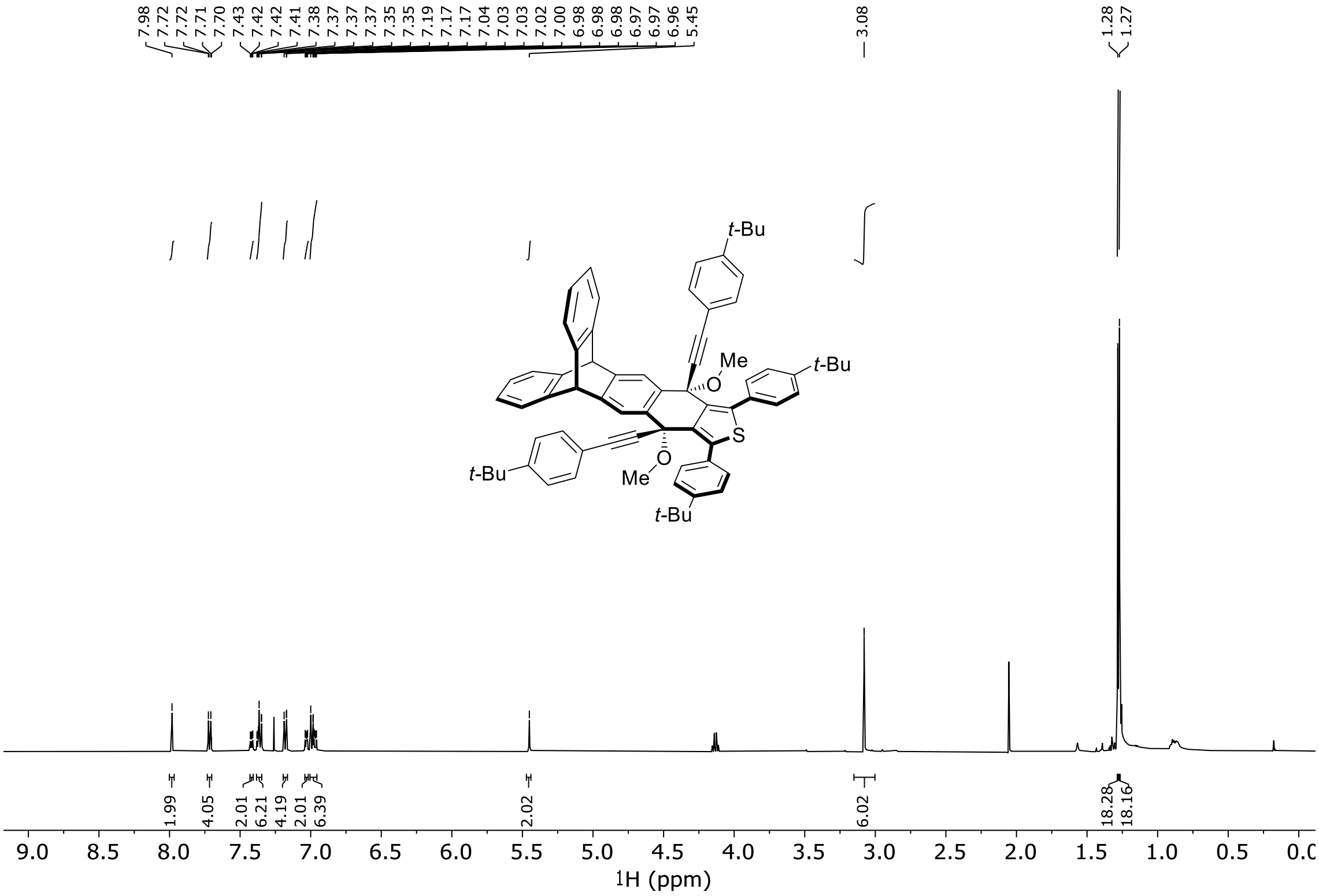

Spectrum S15: ^{1}H NMR spectrum for compound **9**.

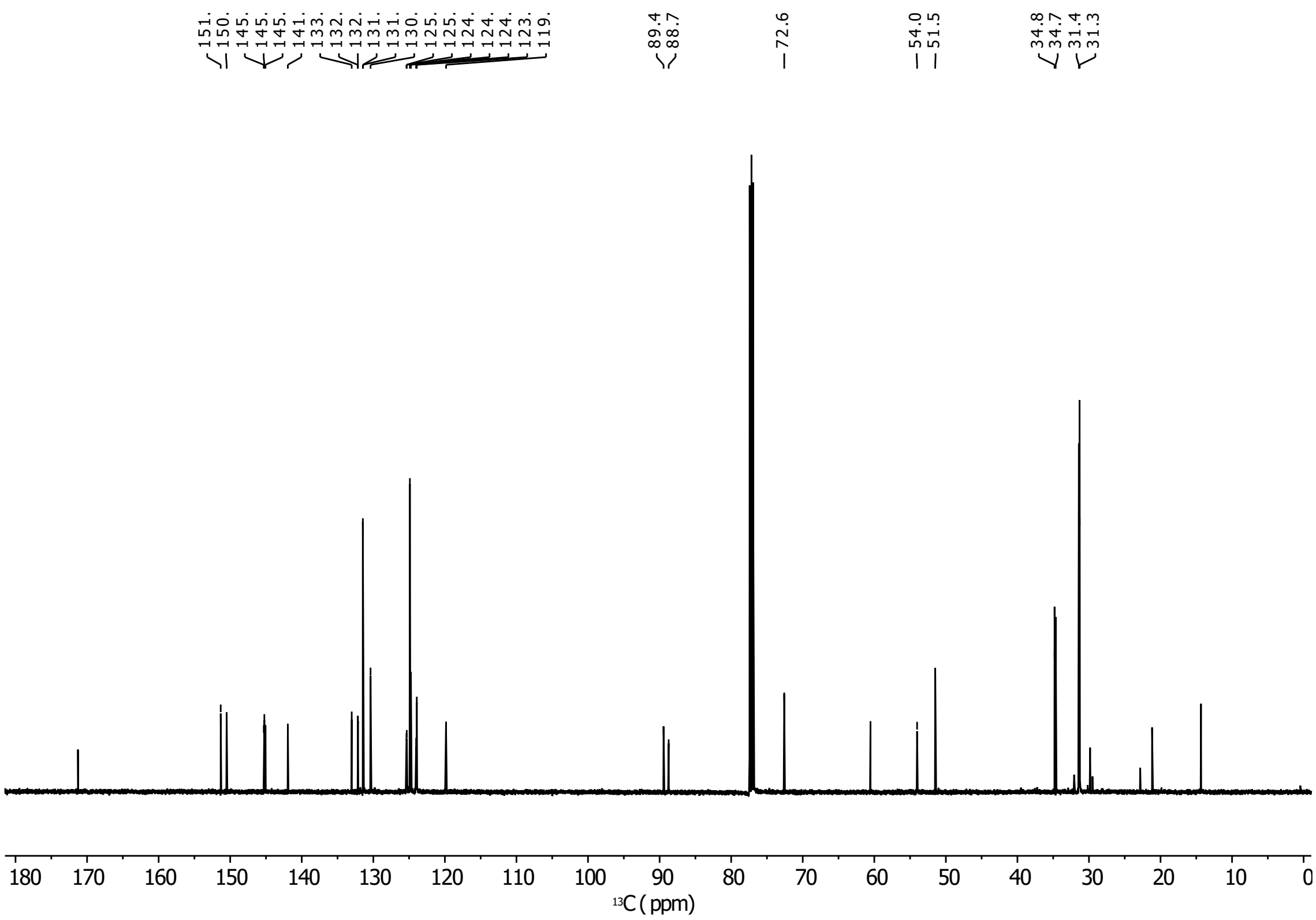

Spectrum S16: ^{13}C NMR spectrum for compound **9**.

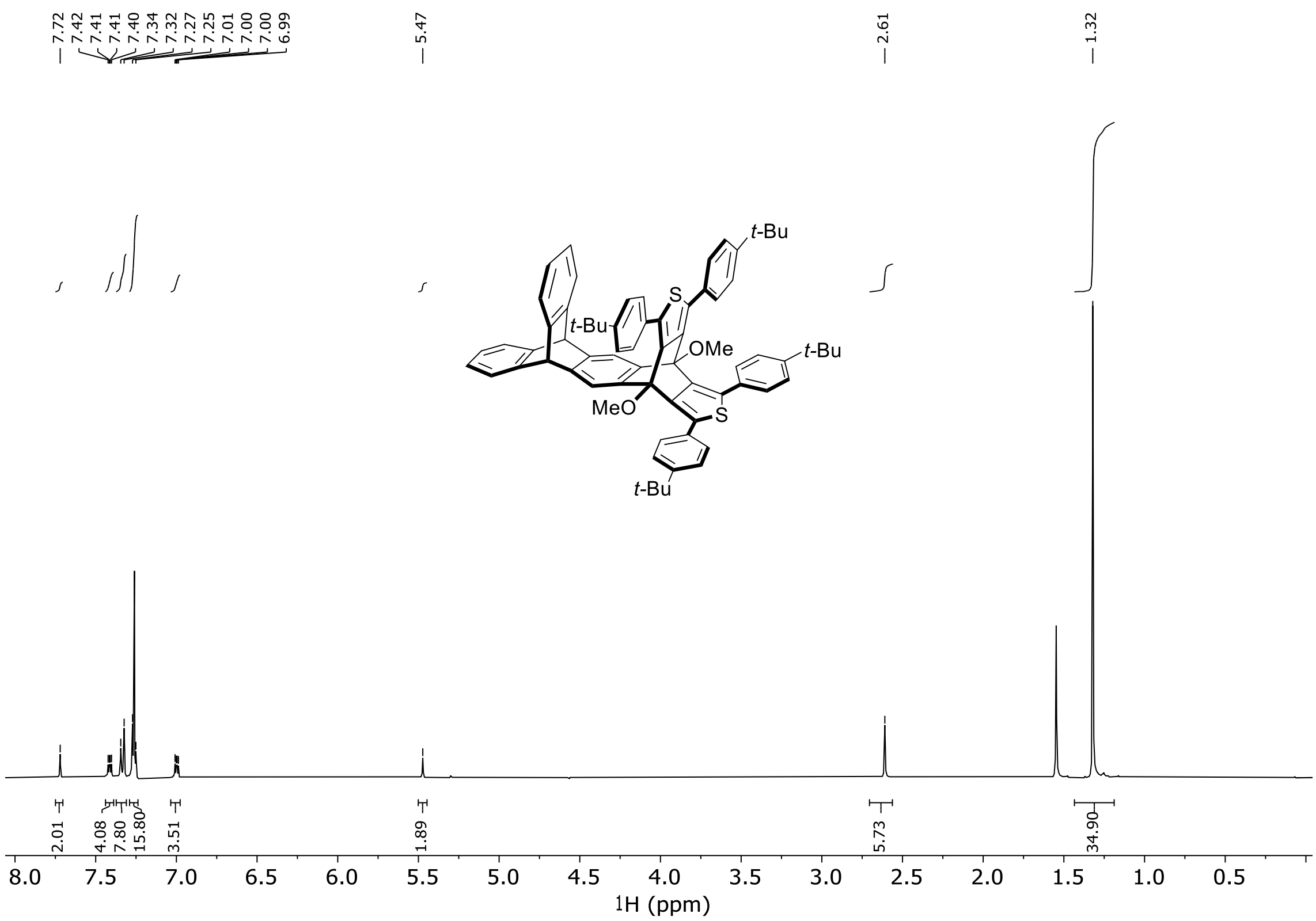

Spectrum S17: ^{1}H NMR spectrum for compound **2**.

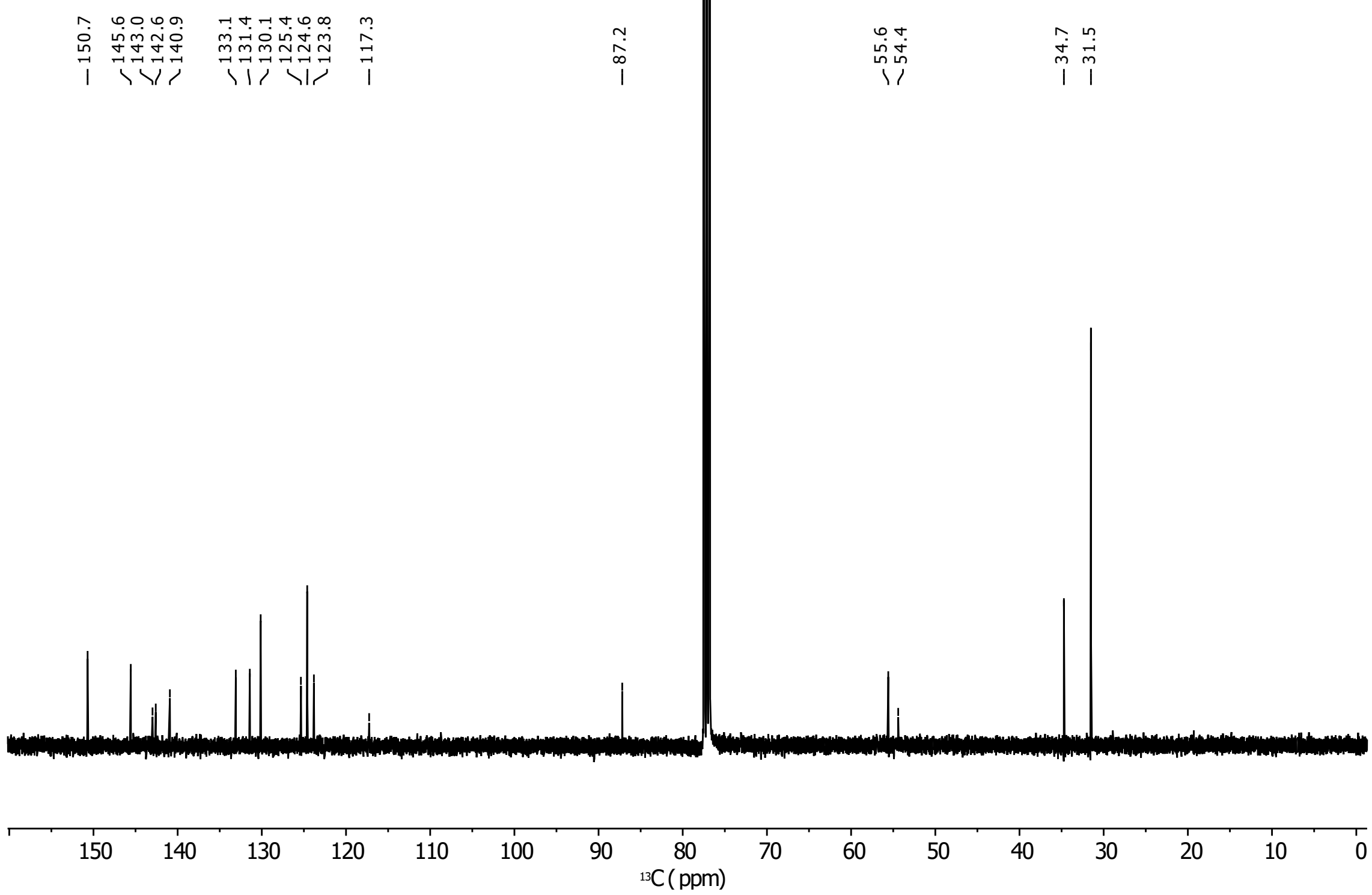

Spectrum S18: ^{13}C NMR spectrum for compound **2**.

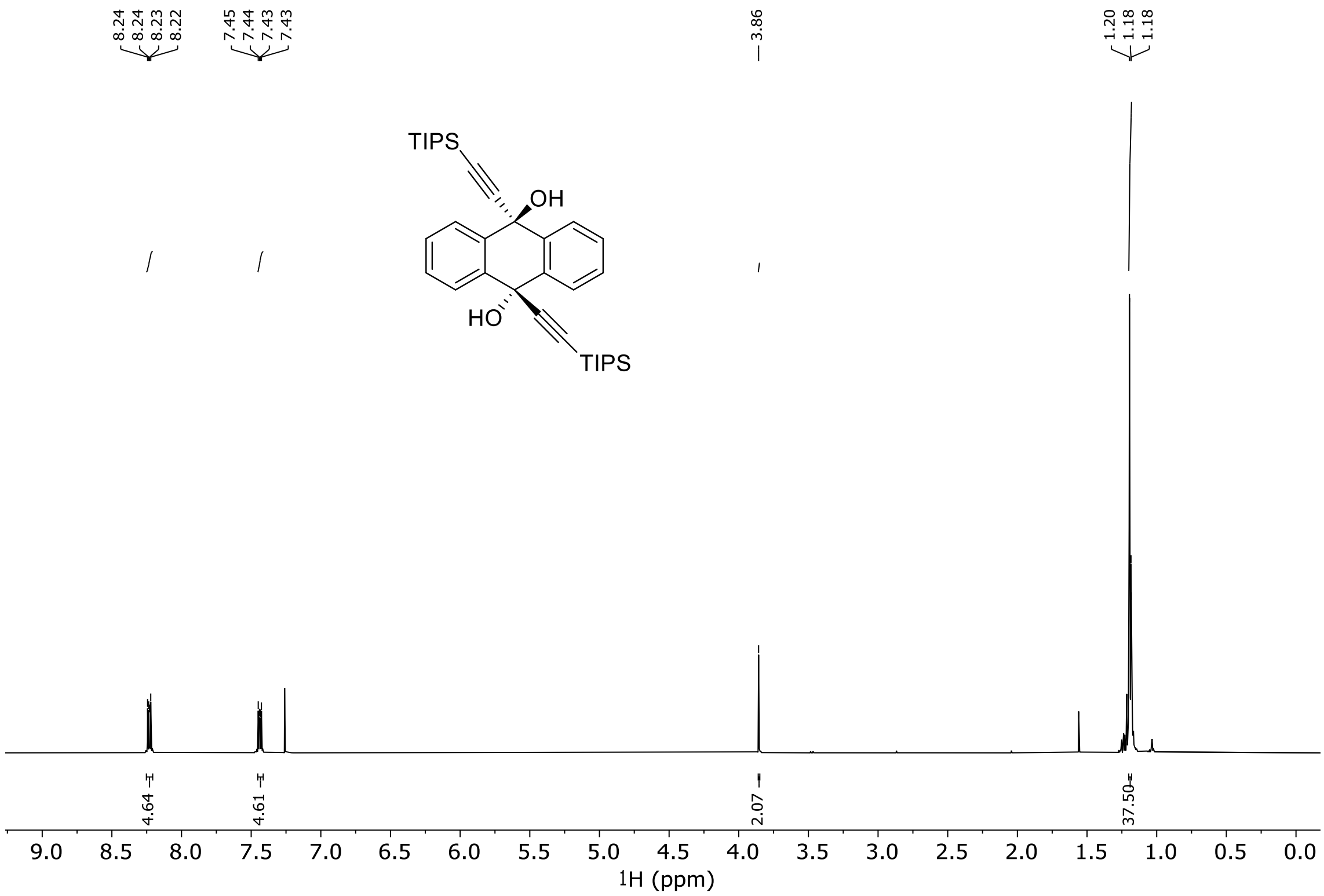

Spectrum S19: ^{1}H NMR spectrum of *trans*-**10**.

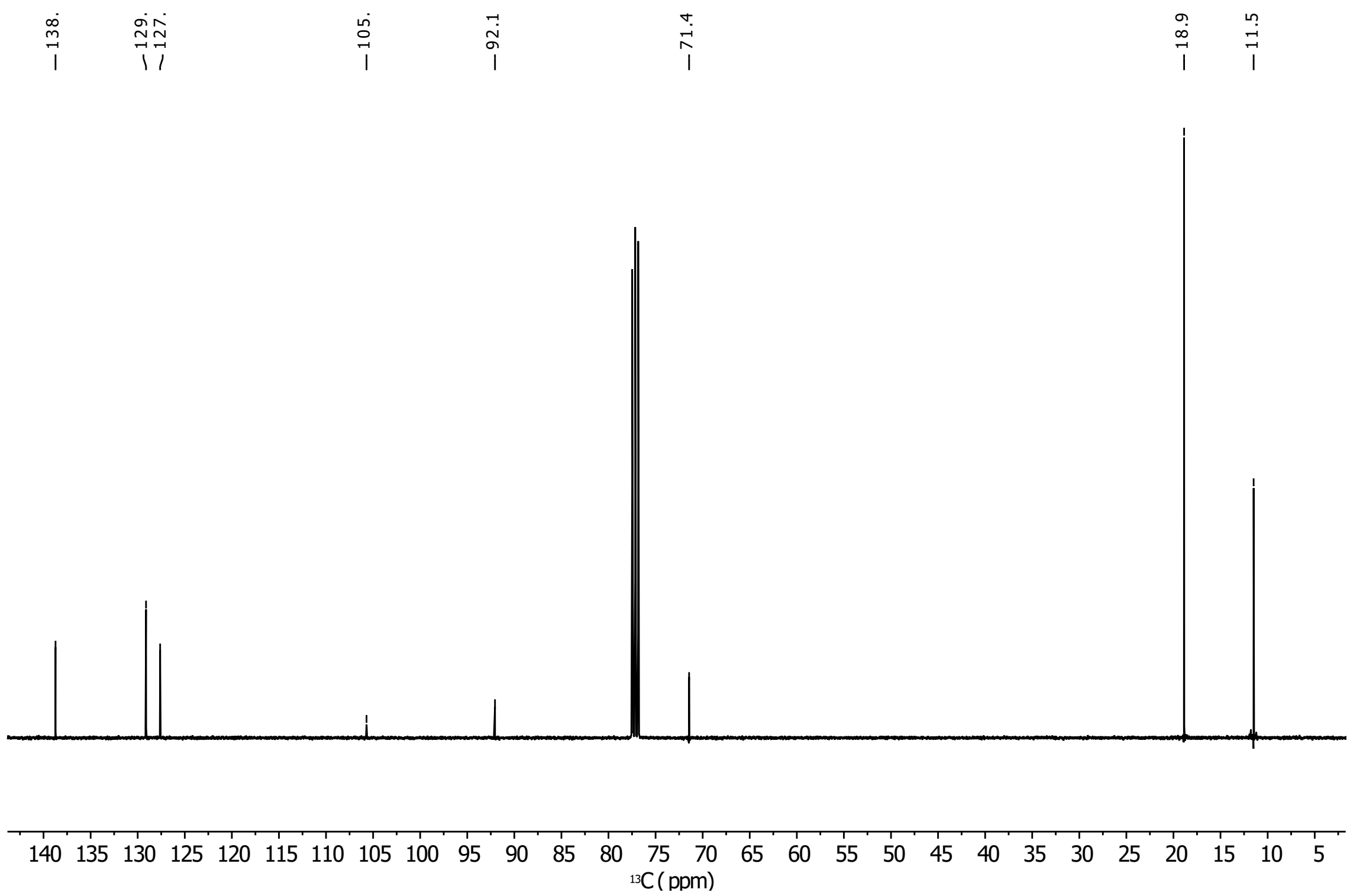

Spectrum S20: ^{13}C NMR spectrum of *trans*-**10**

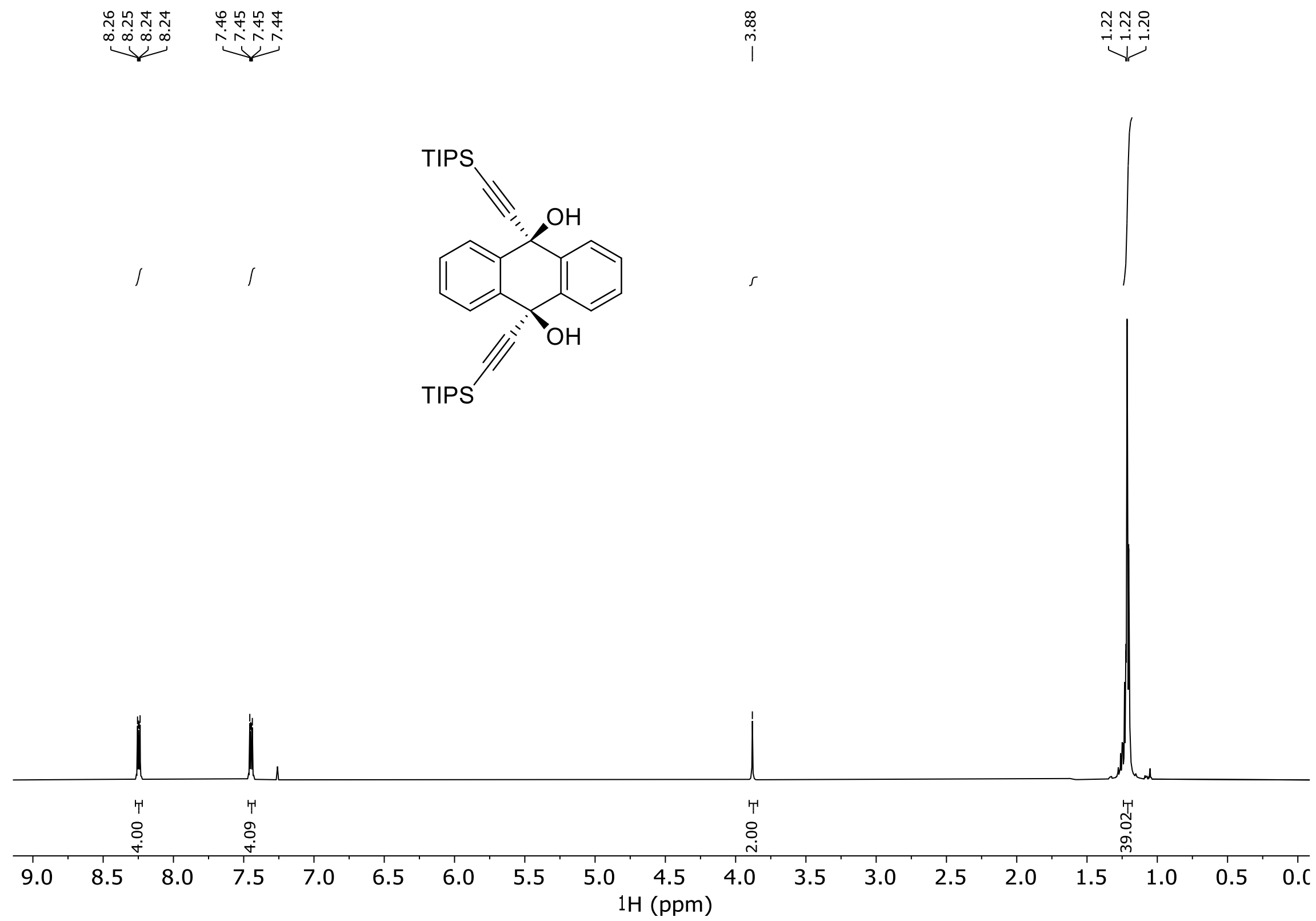

Spectrum S21: ^{1}H NMR spectrum of *cis*-**10**.

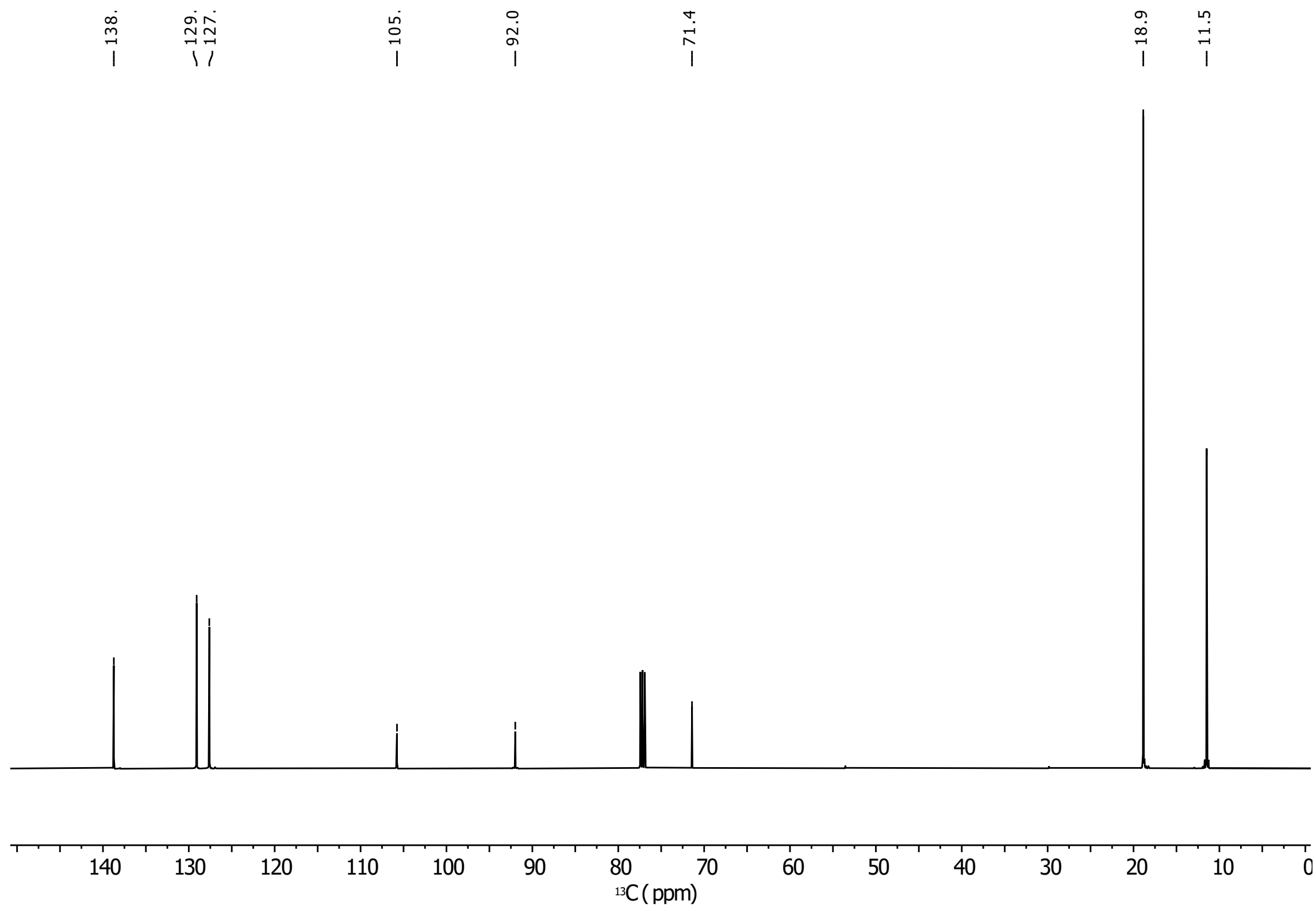

Spectrum S22: ^{13}C NMR spectrum of *cis*-**10**.

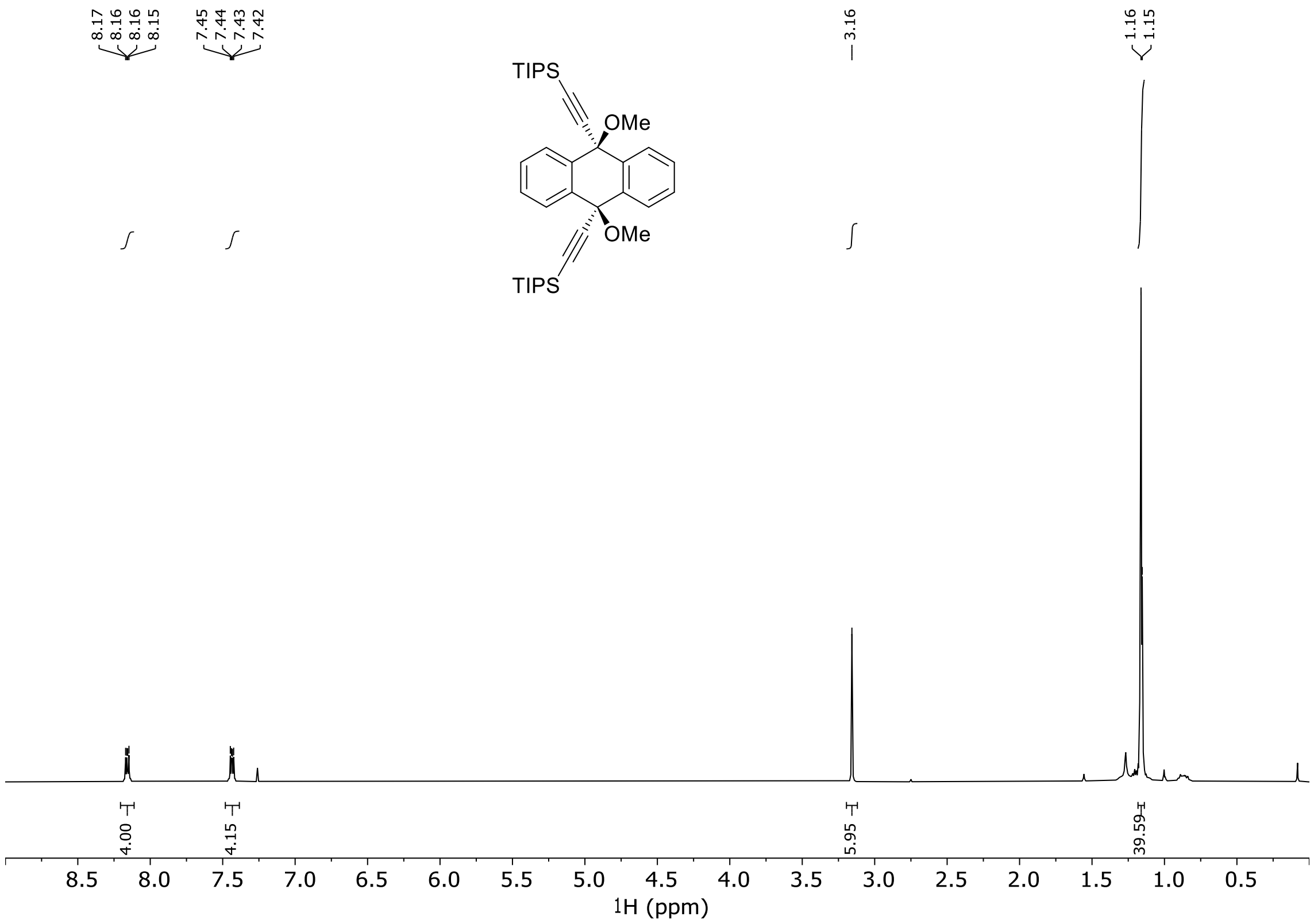

Spectrum S23: ^{1}H NMR spectrum of *cis*-**11**.

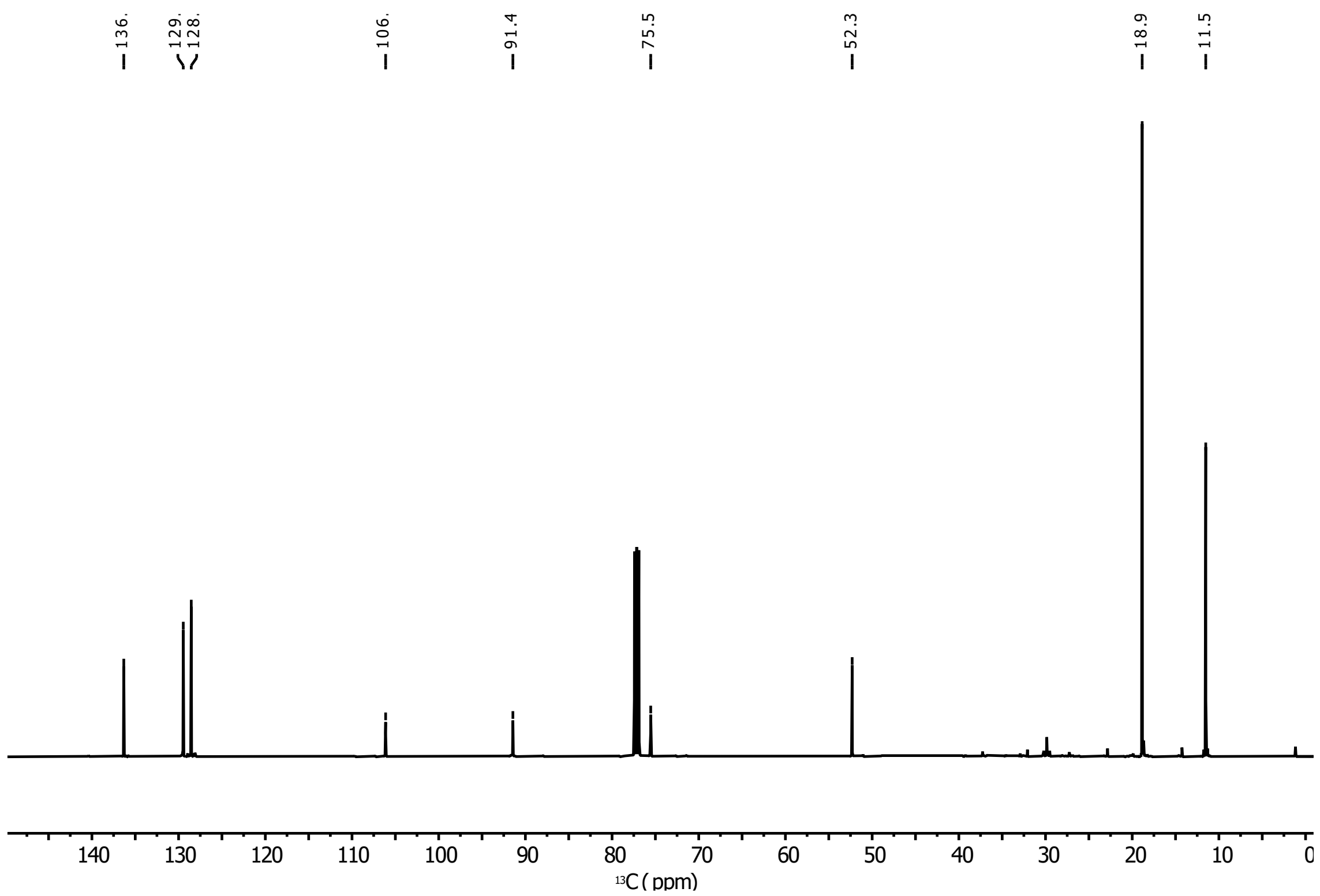

Spectrum S24: ^{13}C NMR spectrum of *cis*-**11**.

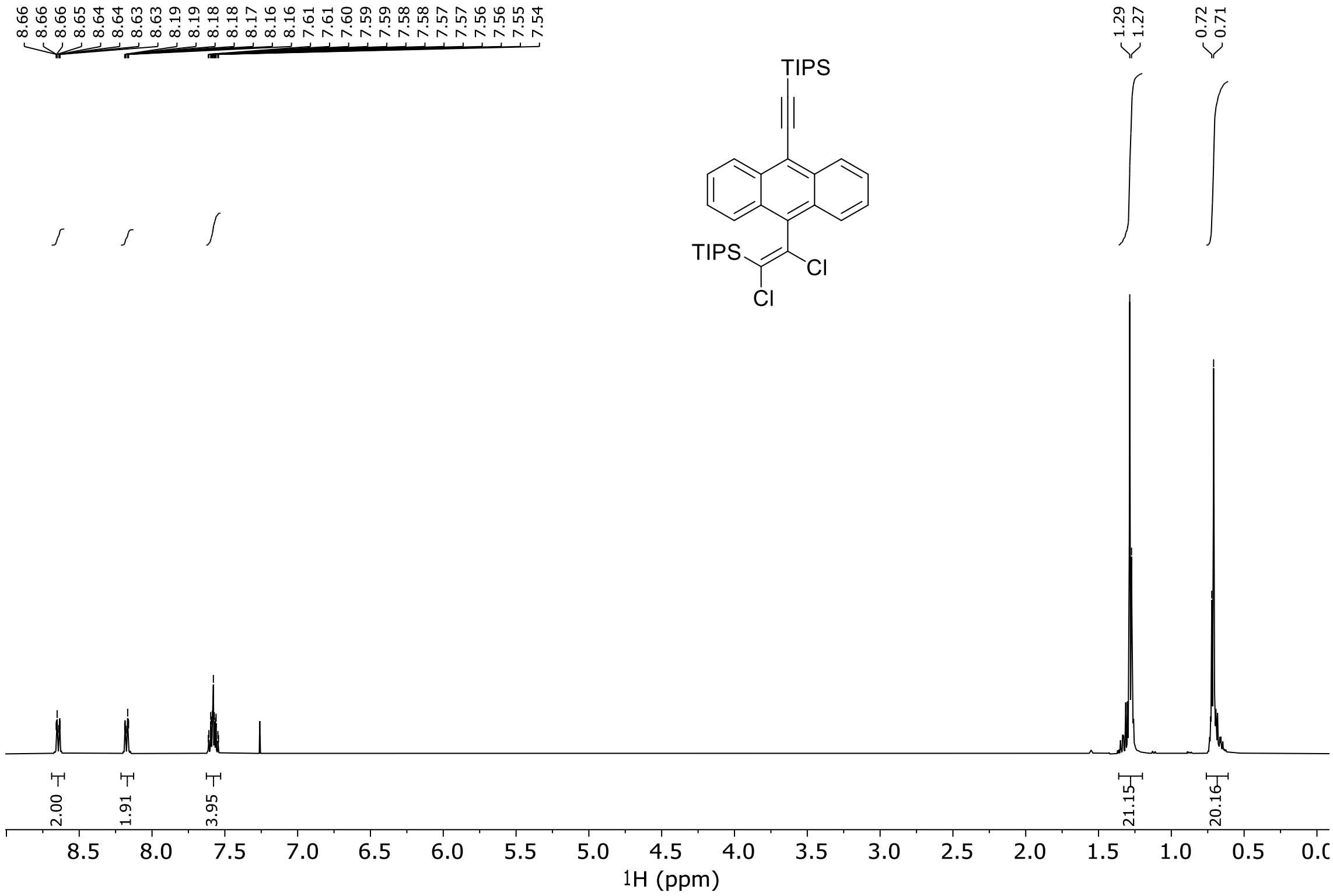

Spectrum S25: ^{1}H NMR spectrum of **12**.

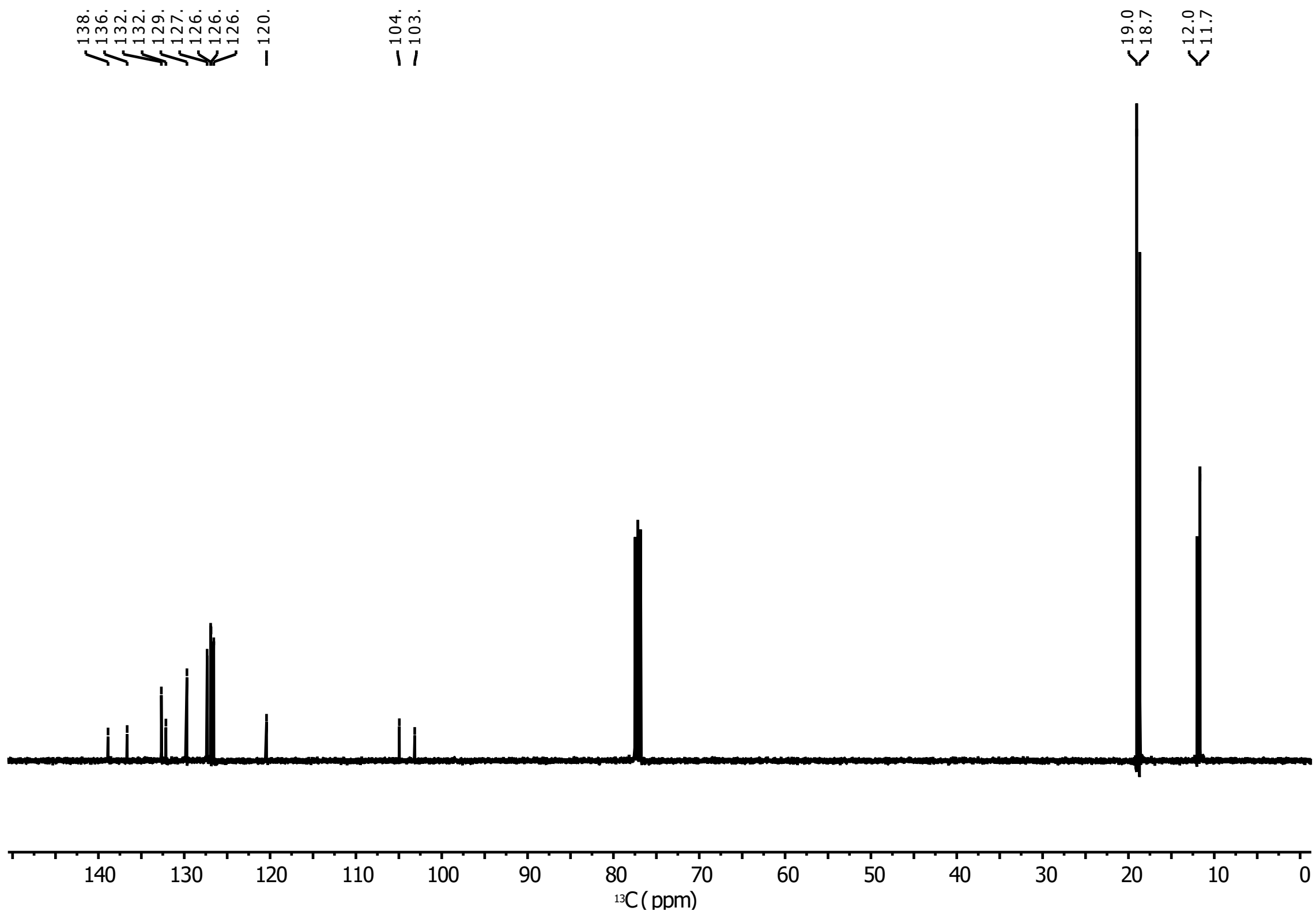

Spectrum S26: ^{13}C NMR spectrum of **12**.

HRMS Spectra

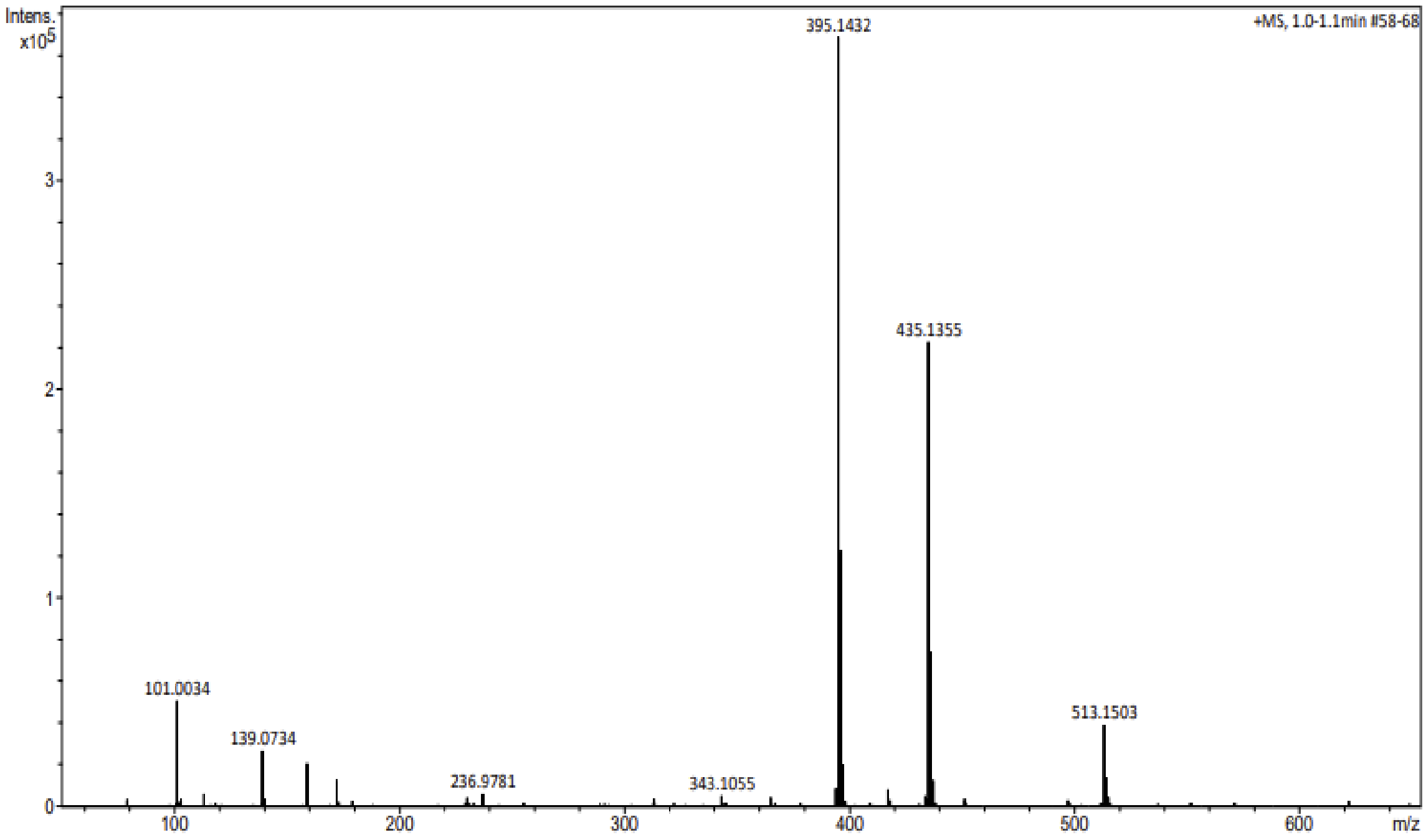

Spectrum S27: Compound *cis*-**3** HRMS spectrum.

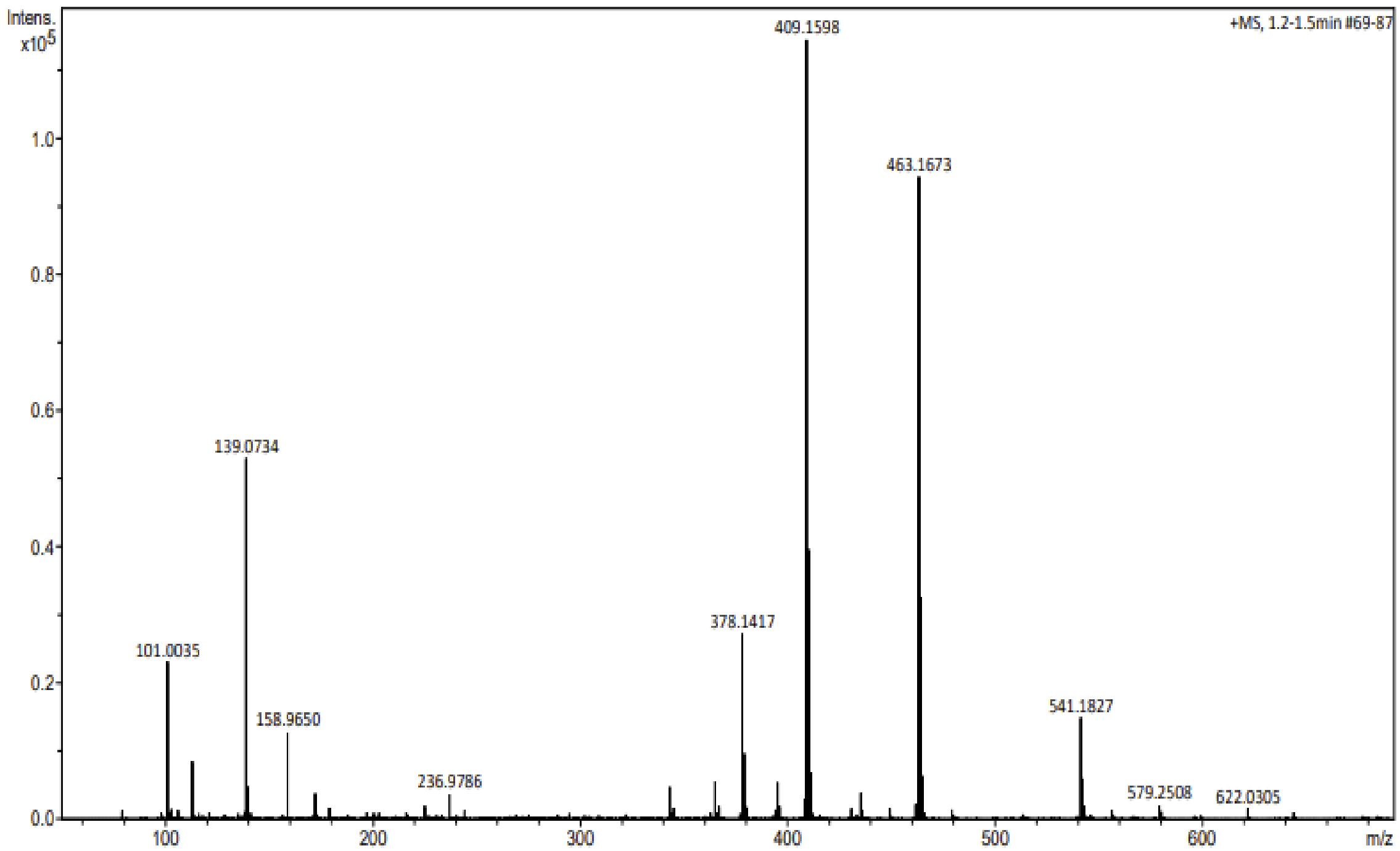

Spectrum S28: Compound *cis*-**4** HRMS spectrum.

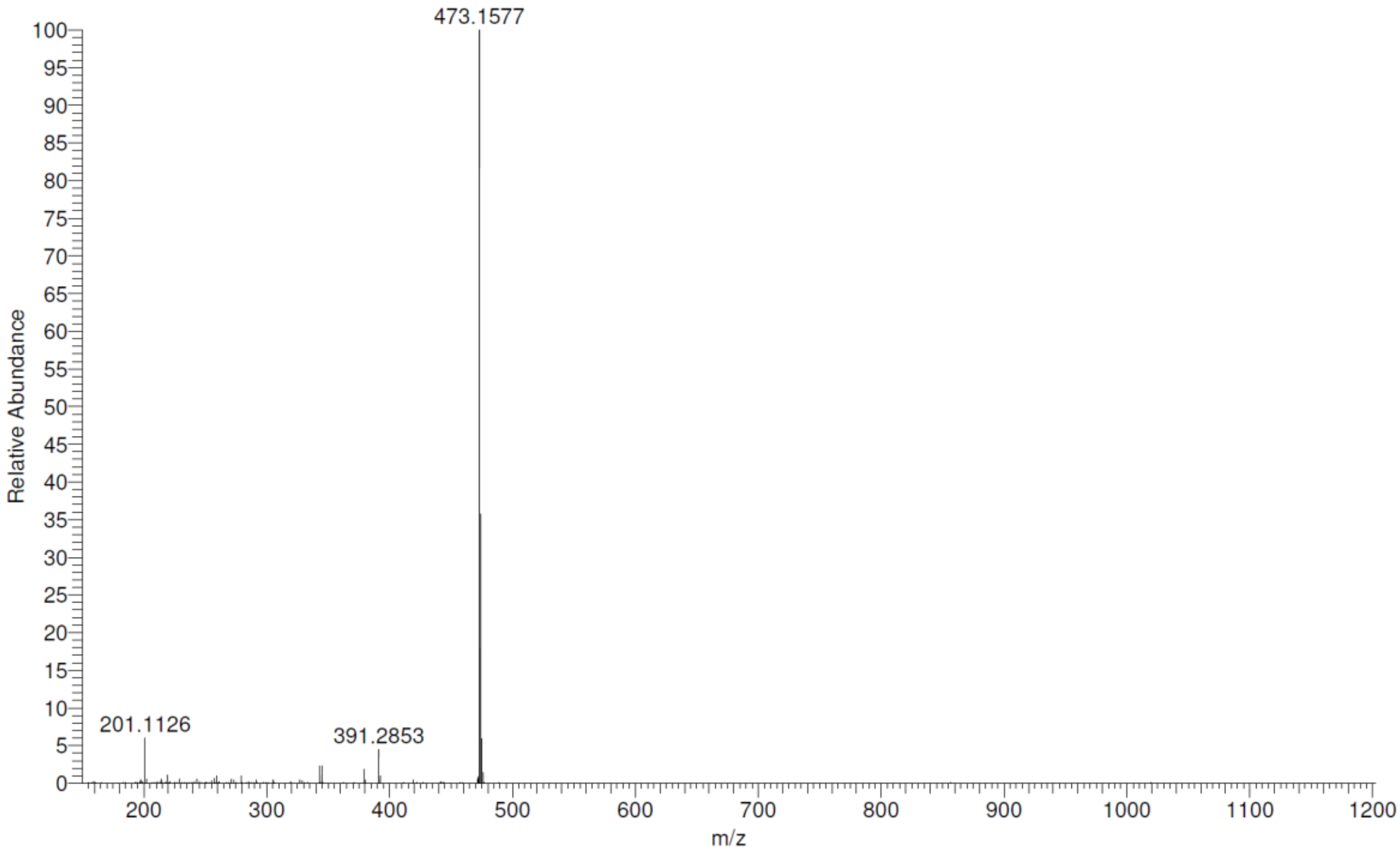

Spectrum S29: Compound **1** HRMS spectrum.

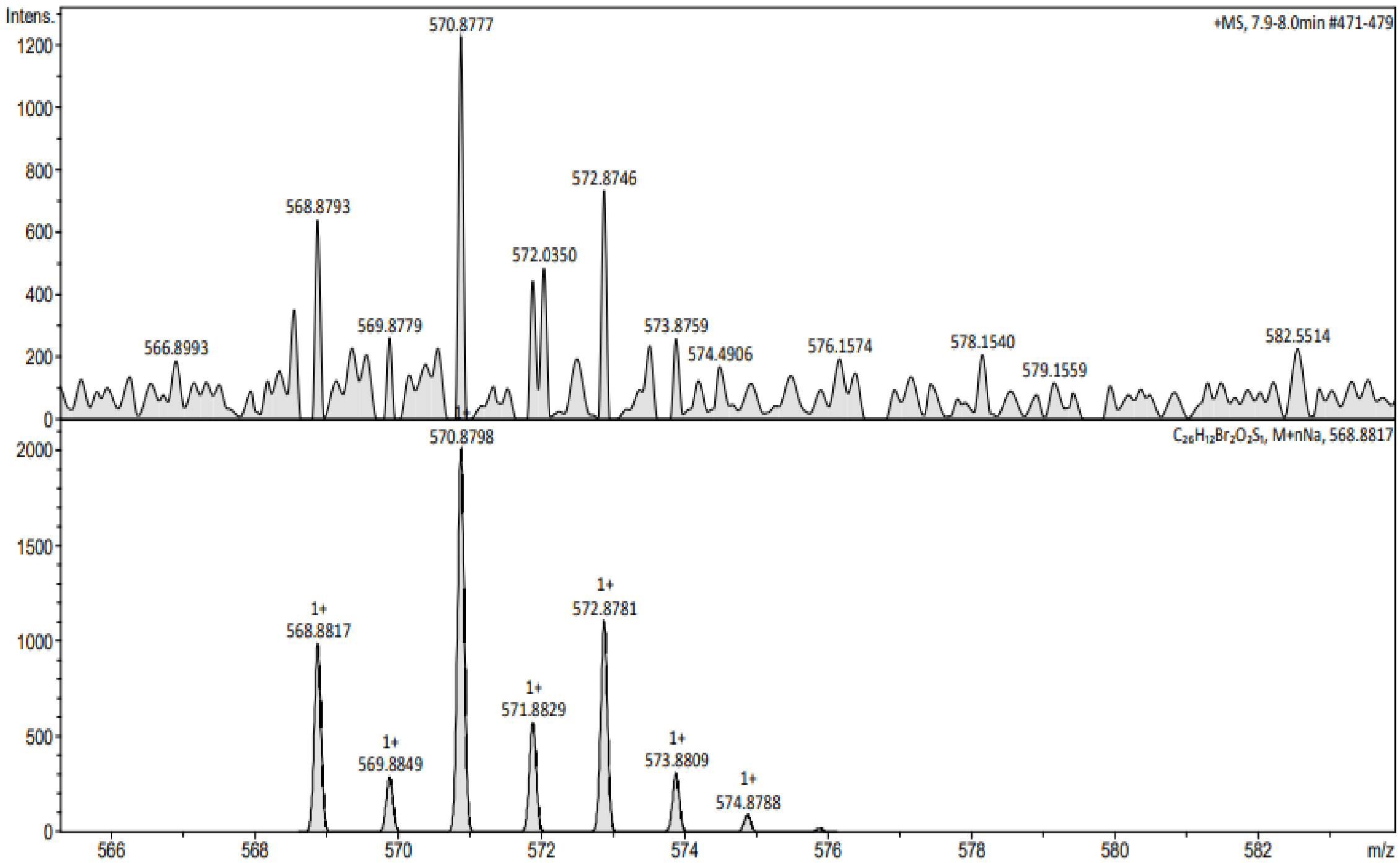

Spectrum S30: Compound β,β-**6** HRMS spectrum.

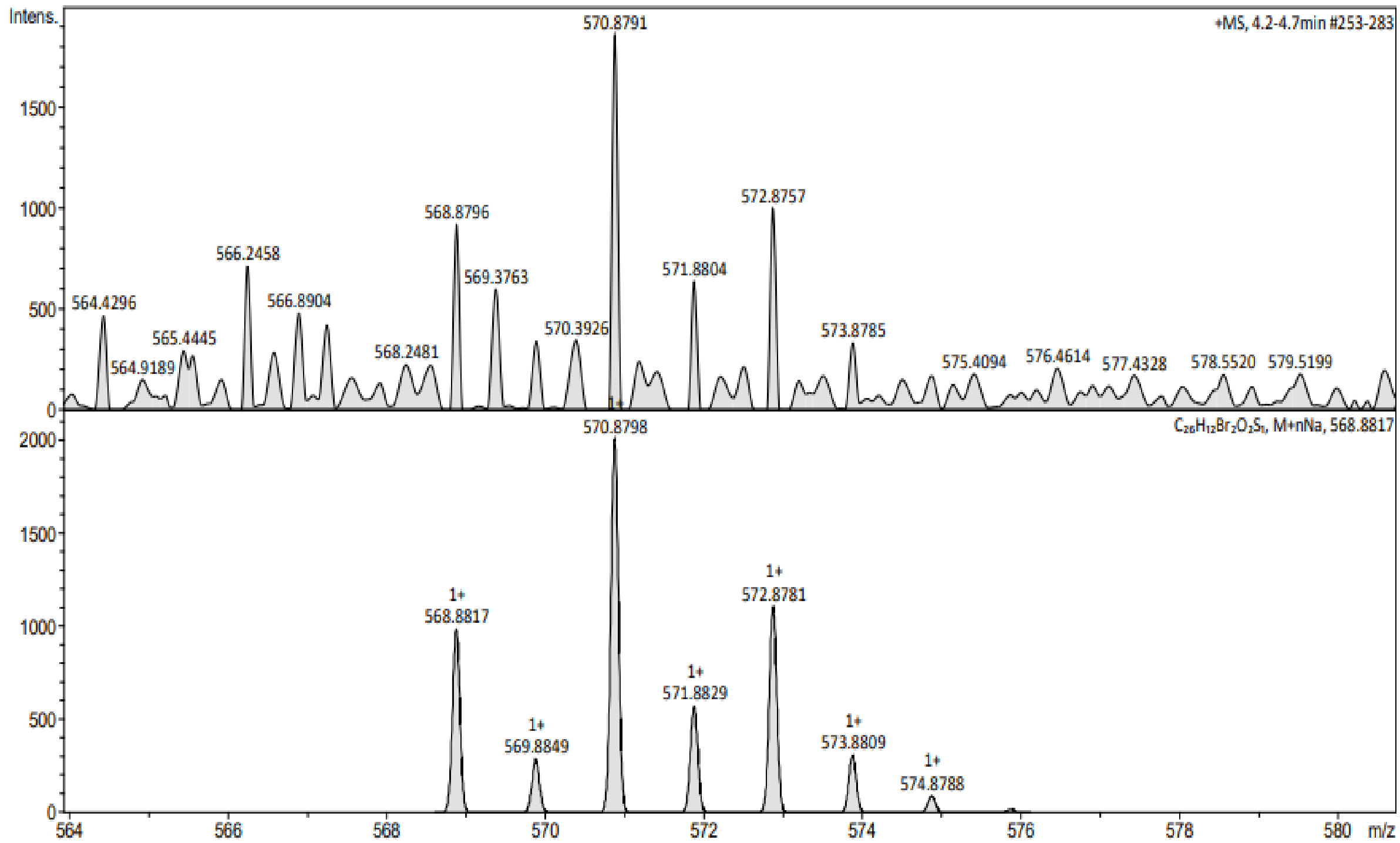

Spectrum S31: Compound α,β-**6** HRMS spectrum.

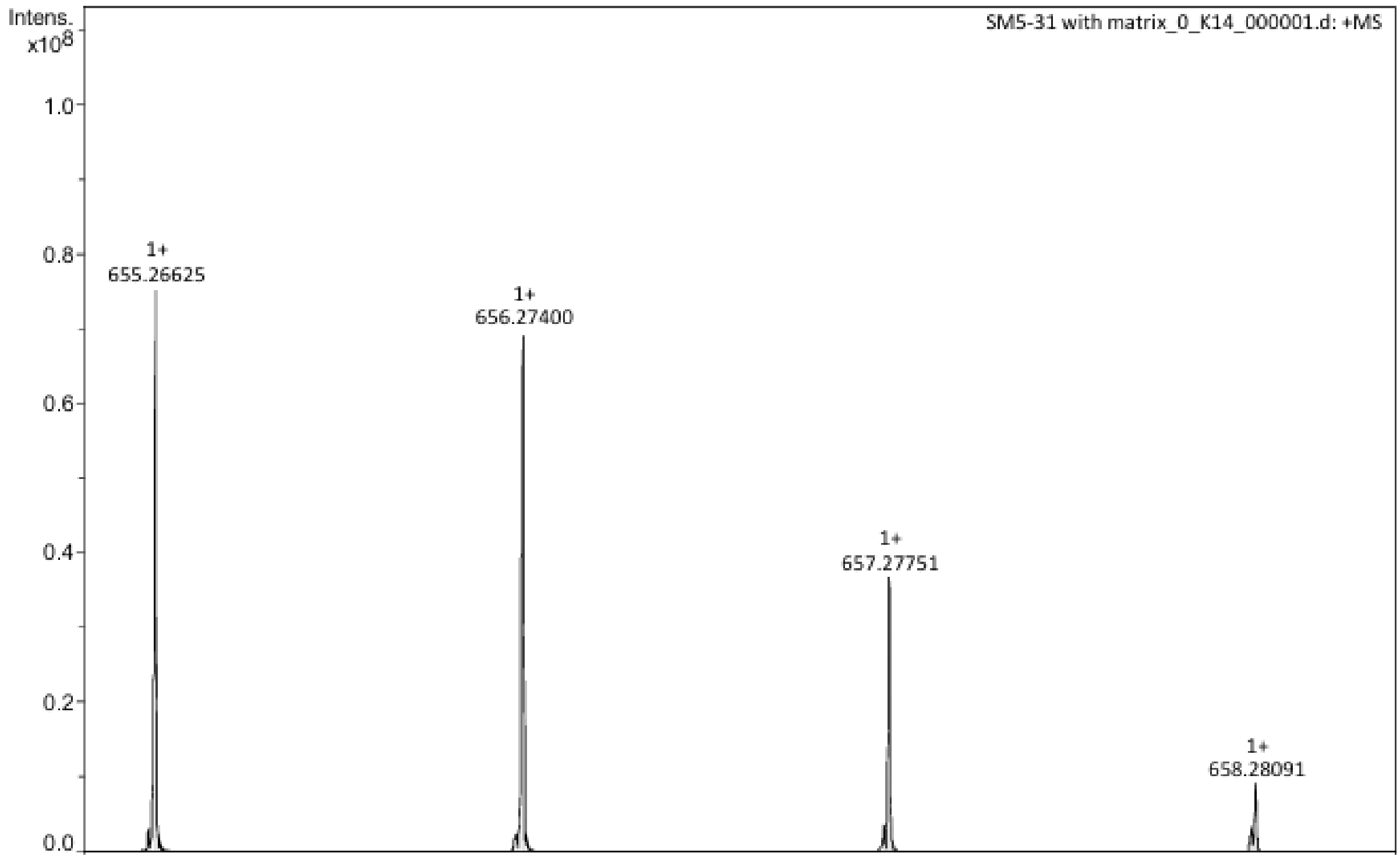

Spectrum S32: Compound **7** HRMS spectrum.

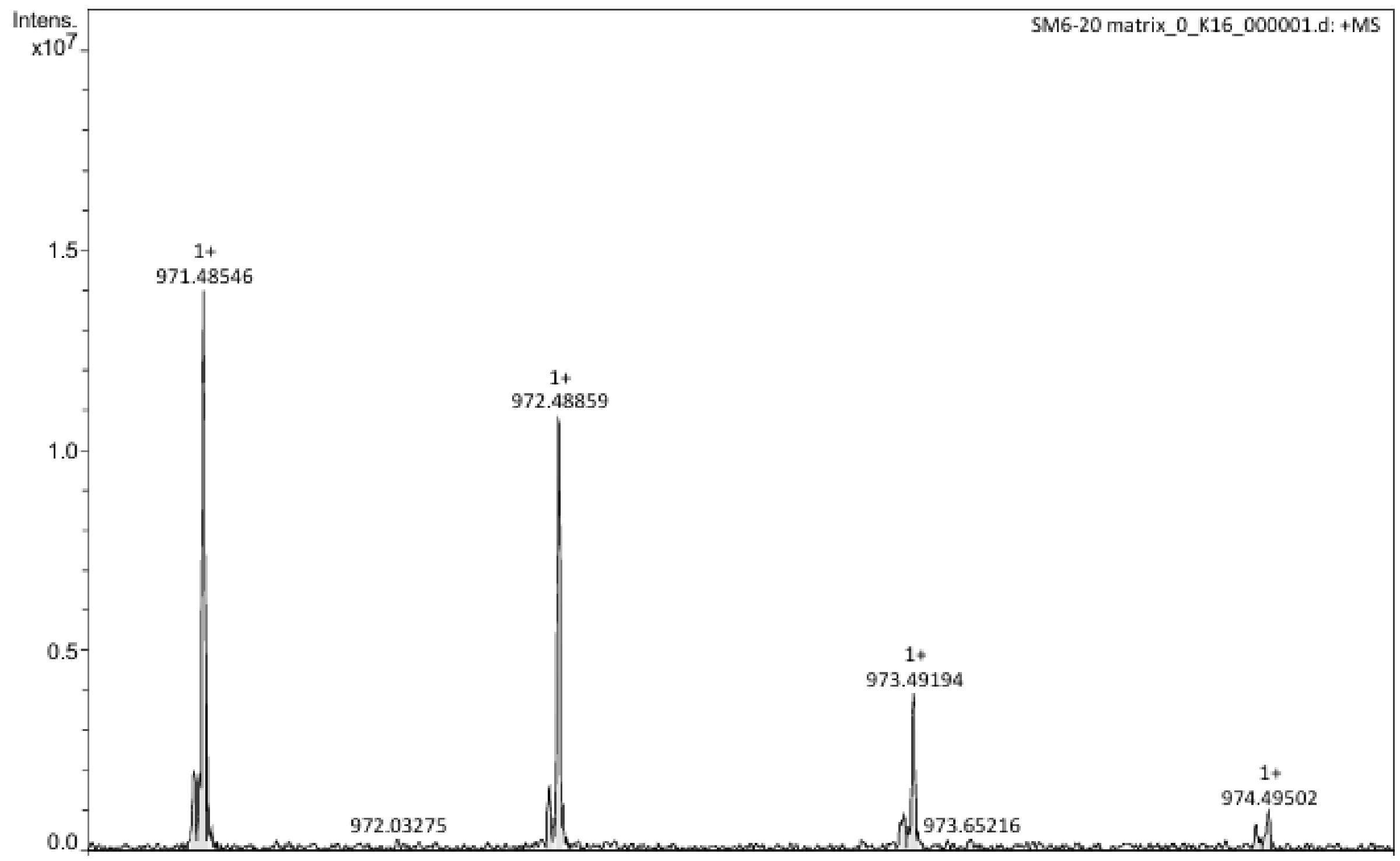

Spectrum S33: Compound **8** HRMS spectrum.

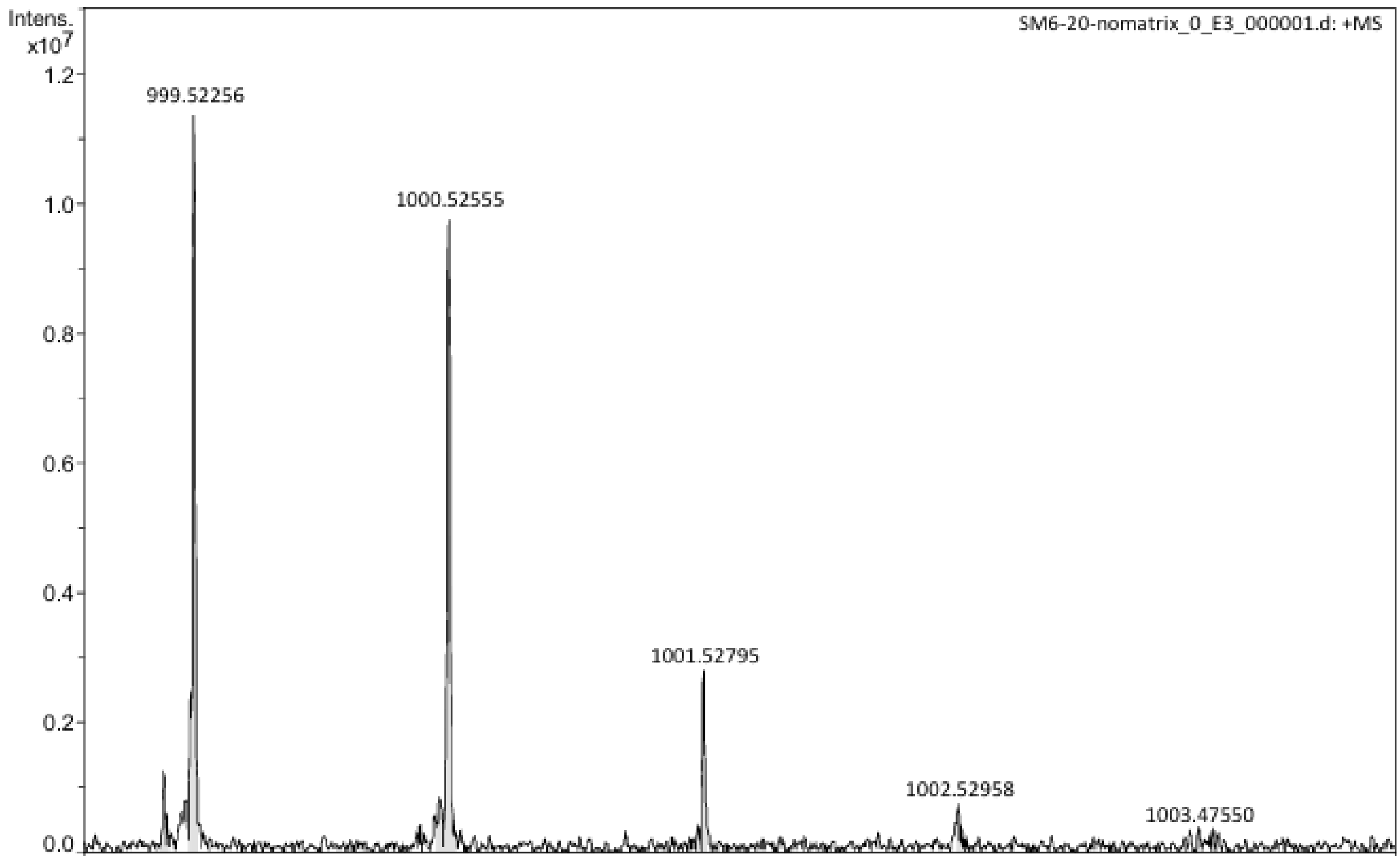

Spectrum S34: Compound **9** HRMS spectrum.

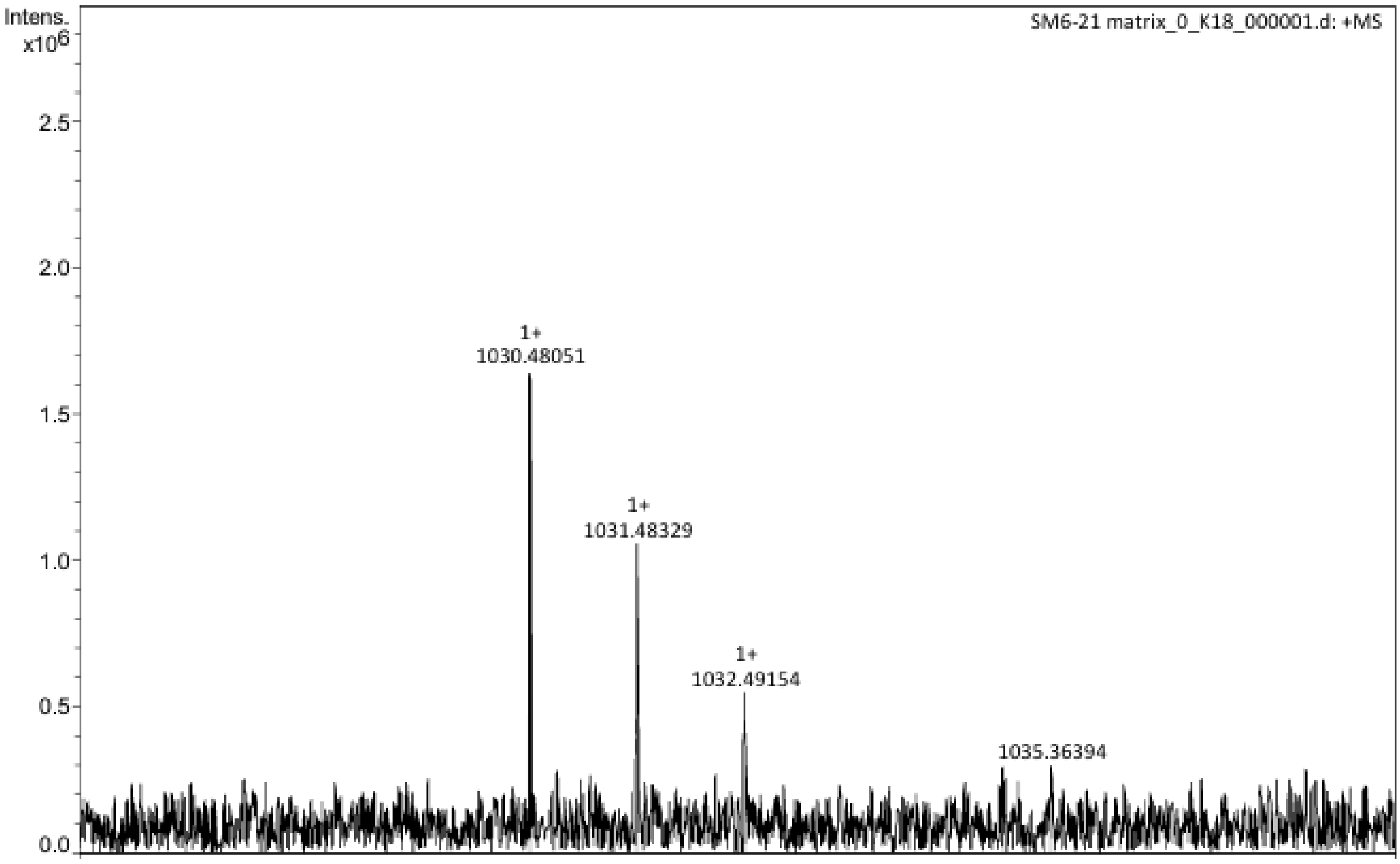

Spectrum S35: Compound **2** HRMS spectrum.

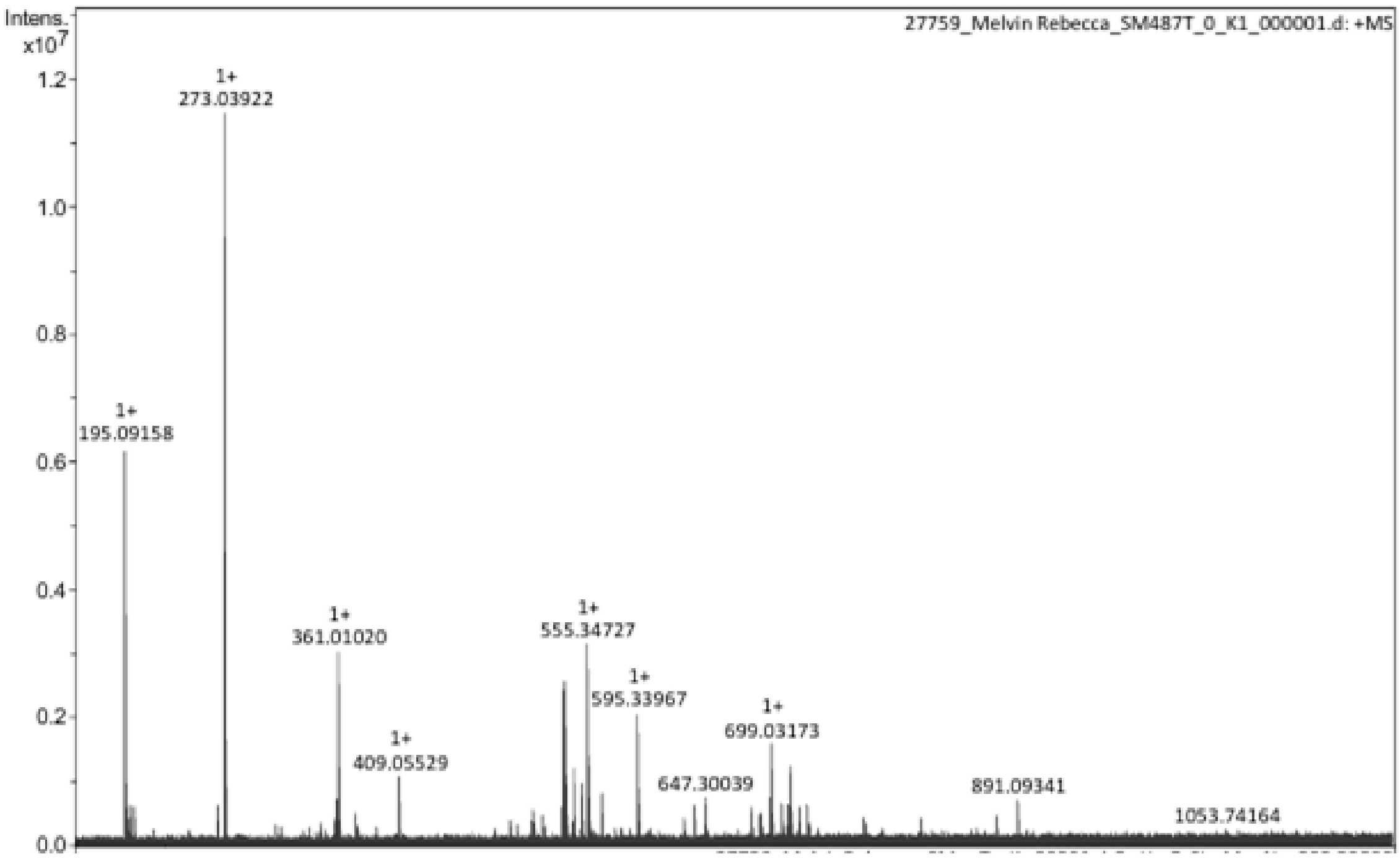

Spectrum S36: Compound *trans*-**10** HRMS spectrum.

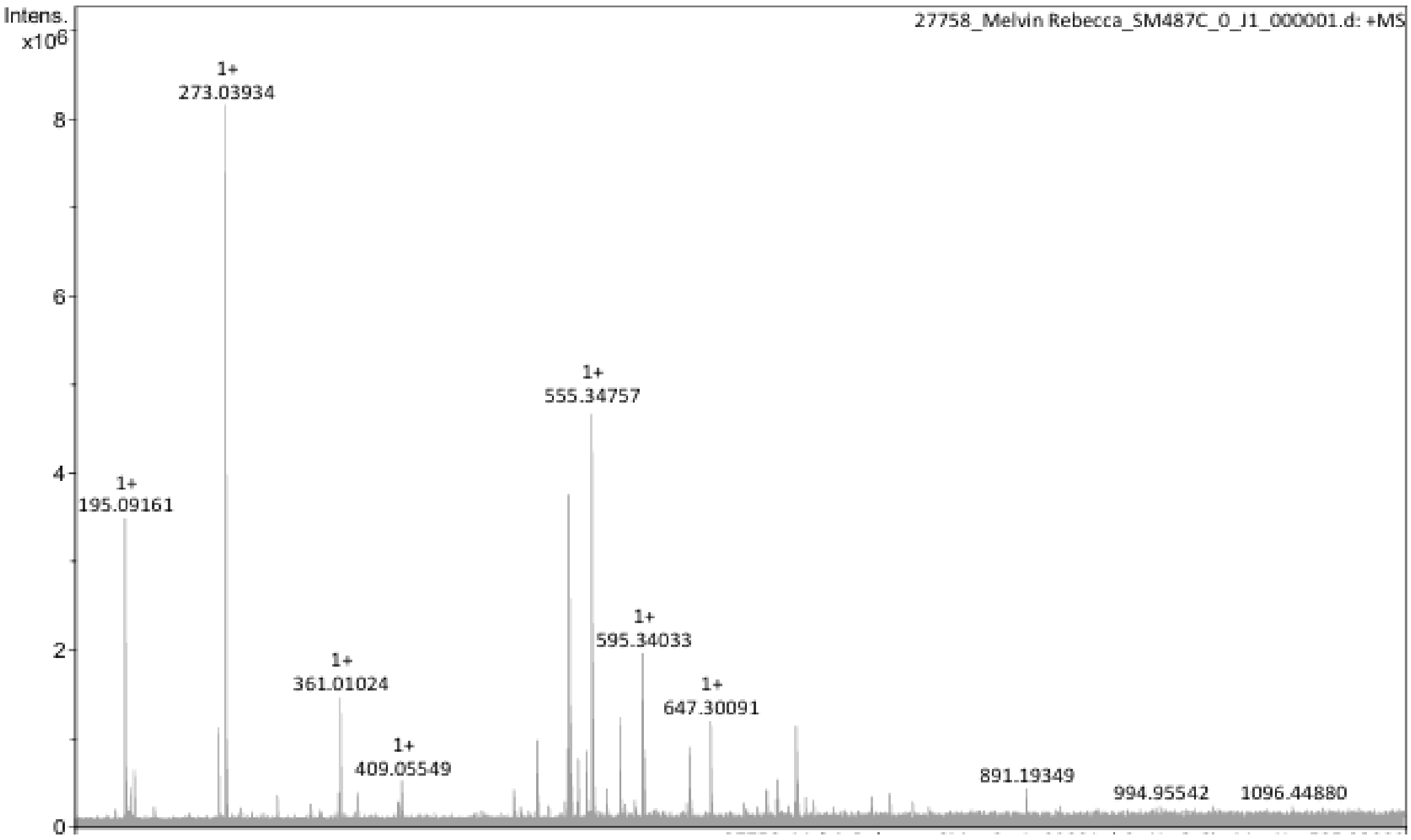

Spectrum S37: Compound *cis*-**10** HRMS spectrum.

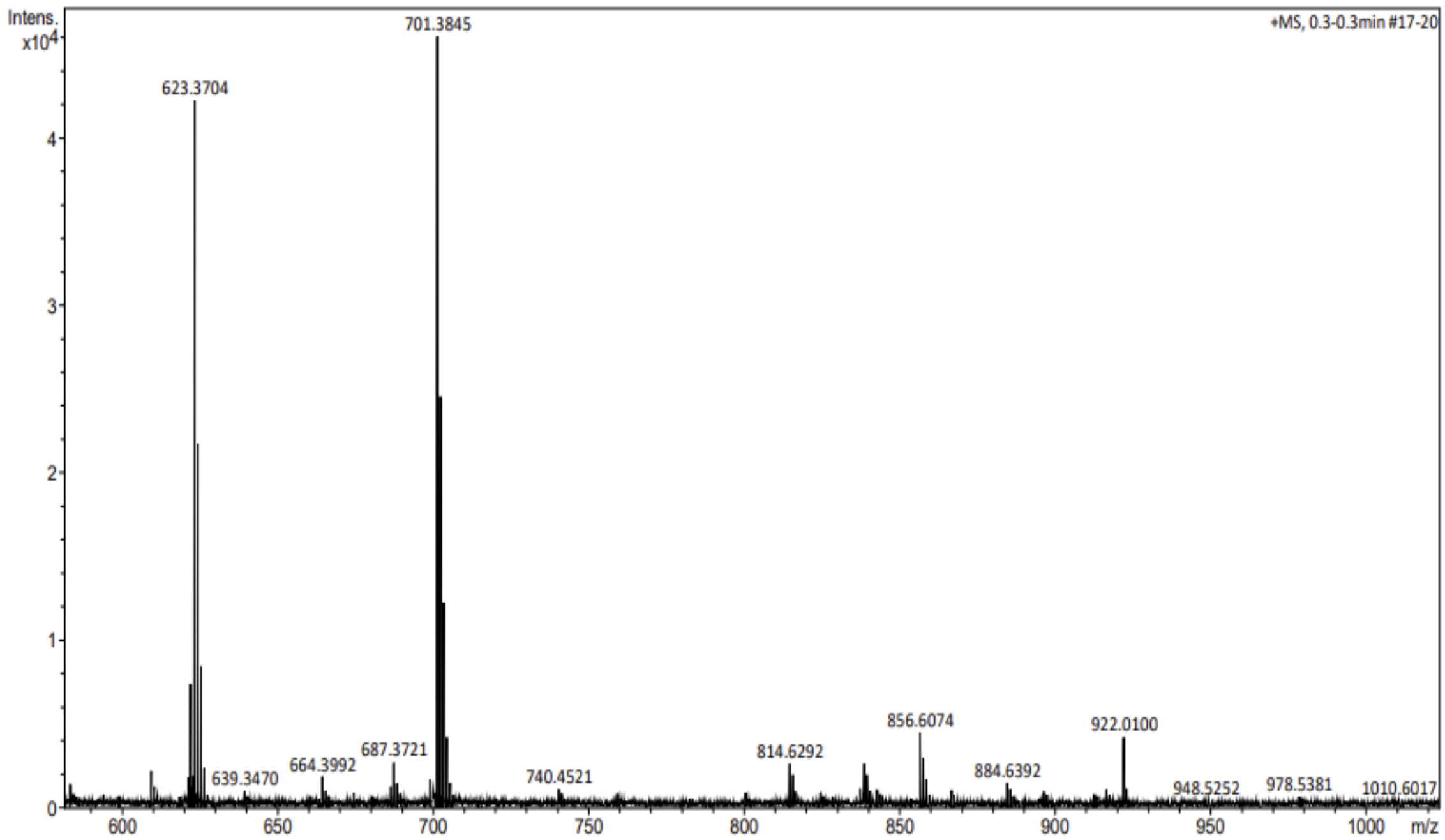

Spectrum S38: Compound *cis*-**11** HRMS spectrum.

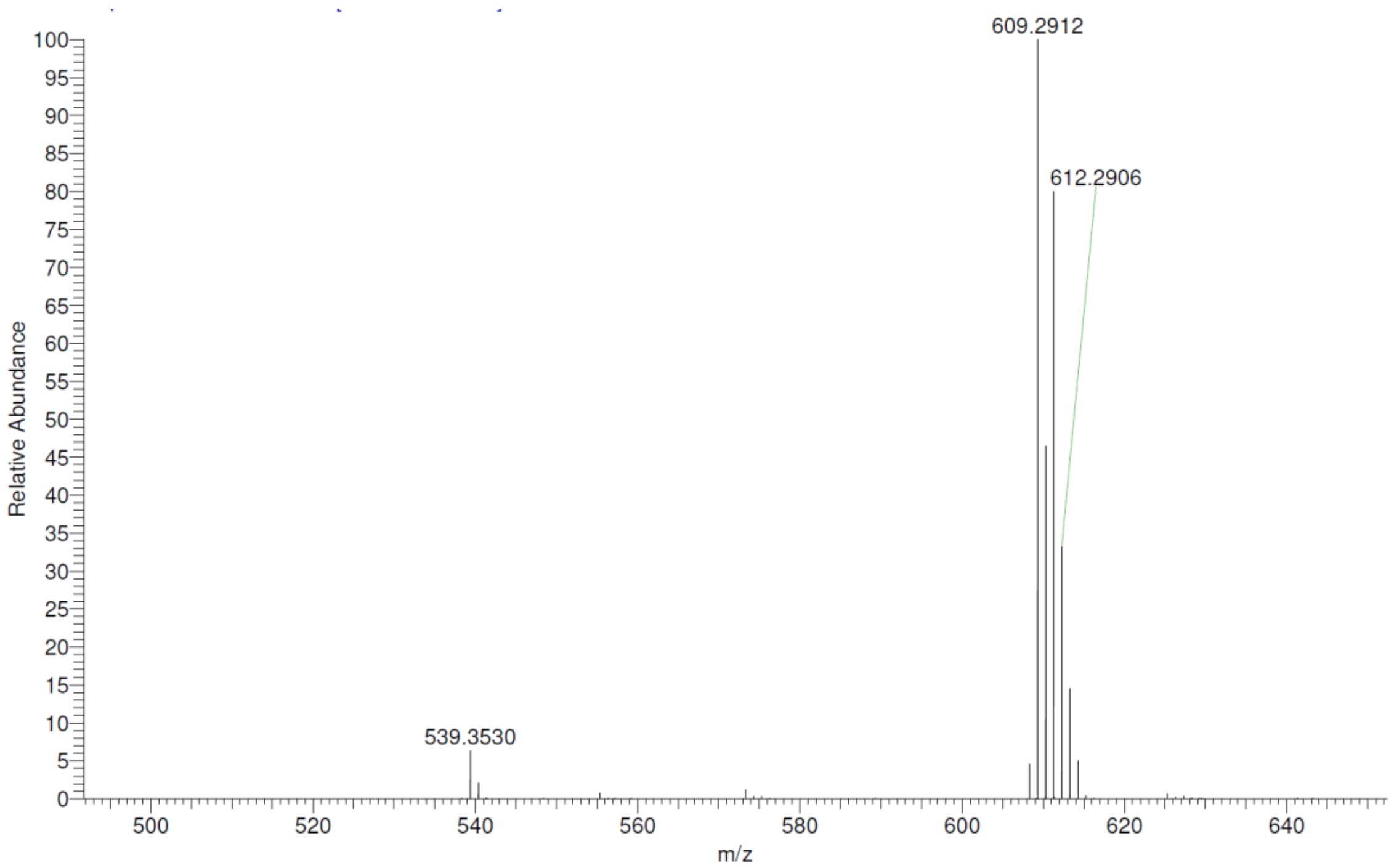

Spectrum S39: Compound **12** HRMS spectrum.

UV-vis and Emission Spectroscopy

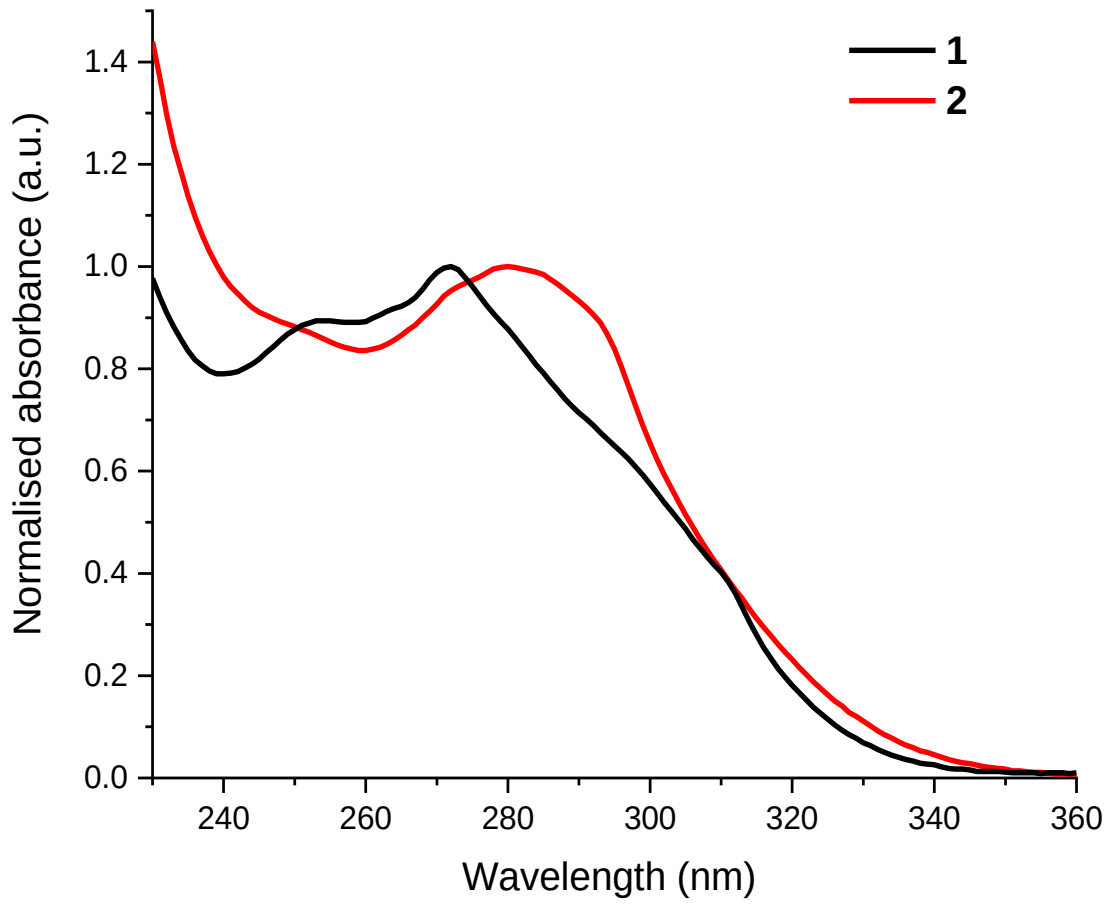

Figure S1: Steady-state UV-vis absorption spectra of **1** and **2** in toluene solution.

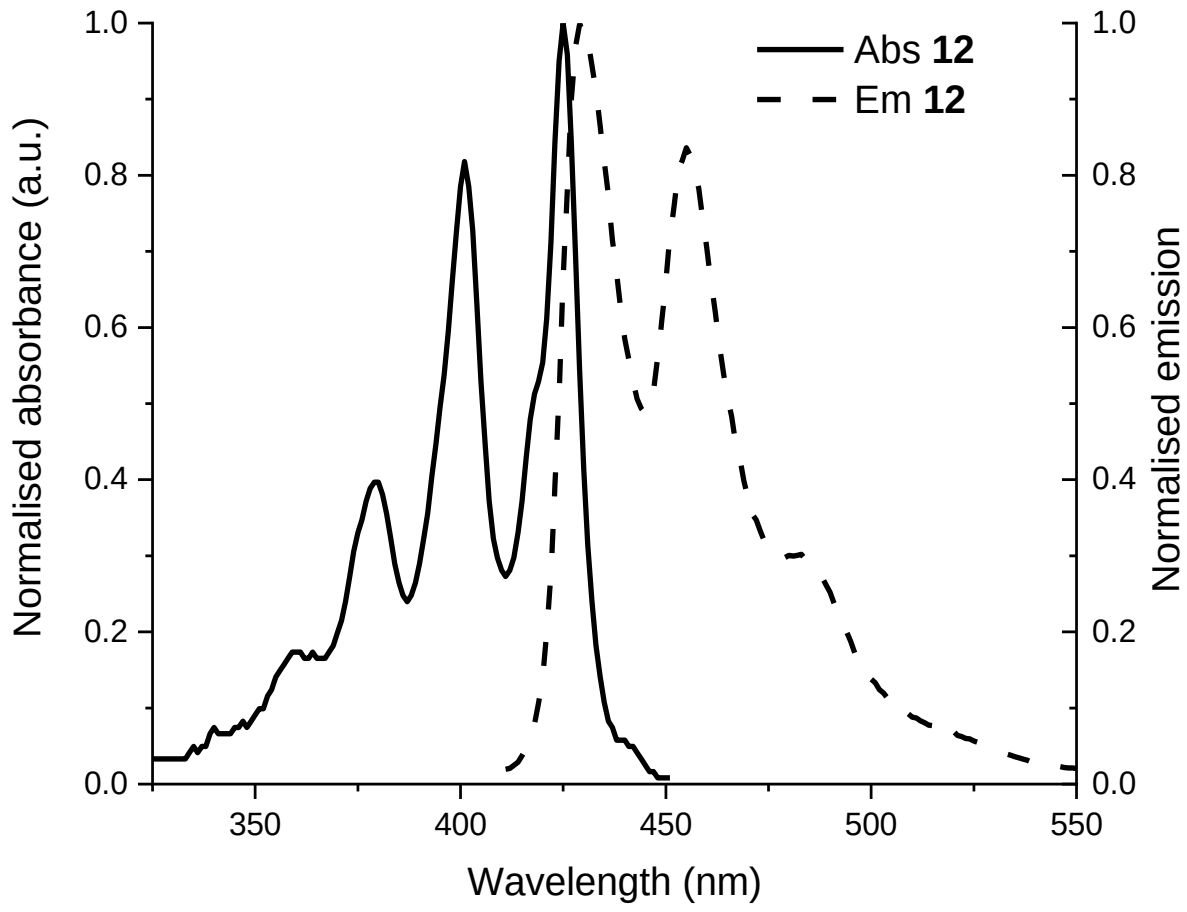

Figure S2: Steady-state UV-vis absorption and photoluminescence spectra of **12** in toluene (excitation wavelength λ_{exc} = 425 nm).

Table S1: UV-vis absorption (λ_{max}) and photoluminescence (λ_{em}) peaks for **1**, **2** and **12** in toluene solution.

	λ_{max} (nm)	λ_{em} (nm)	Stokes Shift (eV)
**1**	272, 294^{sh}, 310 ^{sh}		
**2**	280		
**12**	425, 401, 379, 359	430, 455, 482	0.034

^{sh} = shoulder

X-Ray Crystallography

Diffraction data were collected at low temperature on either a Bruker APEX II CCD diffractometer using graphite-monochromated Mo-Kα X-radiation for *trans*-**10**, *cis*-**10** and **12**, or on a Rigaku diffractometer equipped with a HyPix 6000 detector and a rotating anode Cu-Kα X-ray source for **2**·1.5($CHCl_3$)·3(CH_3OH). The data were corrected for absorption, polarisation, and Lp effects. All structures were solved via a charge-flipping dual-space algorithm.[5] Refinement was on F^2 values with all non-H atoms anisotropic.[6] Refinements were routine except as follows: For **2**·1.5($CHCl_3$)·3(CH_3OH) ^{t}Bu groups at C(33), C(57), and C(67) were modelled with methyl groups disordered over two sets of positions with major site occupancy of 79(2), 67(4), and 75.6(6)%, respectively. There are 1.5 $CHCl_3$ molecules co-crystallised. The chloroform at C(74) was modelled similarly to the ^{t}Bu groups with the three chlorines split over two sets of positions with major site occupancy of 63(2)%. That at C(73) was modelled at half weight as it was clearly not fully occupied. The point atom model suggested 2-3 MeOH per asymmetric unit, badly disordered. Platon Squeeze recovers 431 electrons per unit cell over 4 equal voids, i.e. 54 electrons per asymmetric unit. MeOH has 18 electrons, so actually 3 MeOH per asymmetric unit or 24 per unit cell.[7,8] In *trans*-**10** The central atom, C(70), in an ^{i}Pr group was modelled as split over two positions with major occupancy 52.7(9)%. OH hydrogens were freely refined, with a mild distance restraint in the case of H(2). For *cis*-**10** OH hydrogens had coordinates freely refined with distance restraints while U_{iso} values were tied to those of the carrier atom.

Tables of X-ray data {**S2 – S5**} and supplementary figures {**S3 – S6**} are presented below.

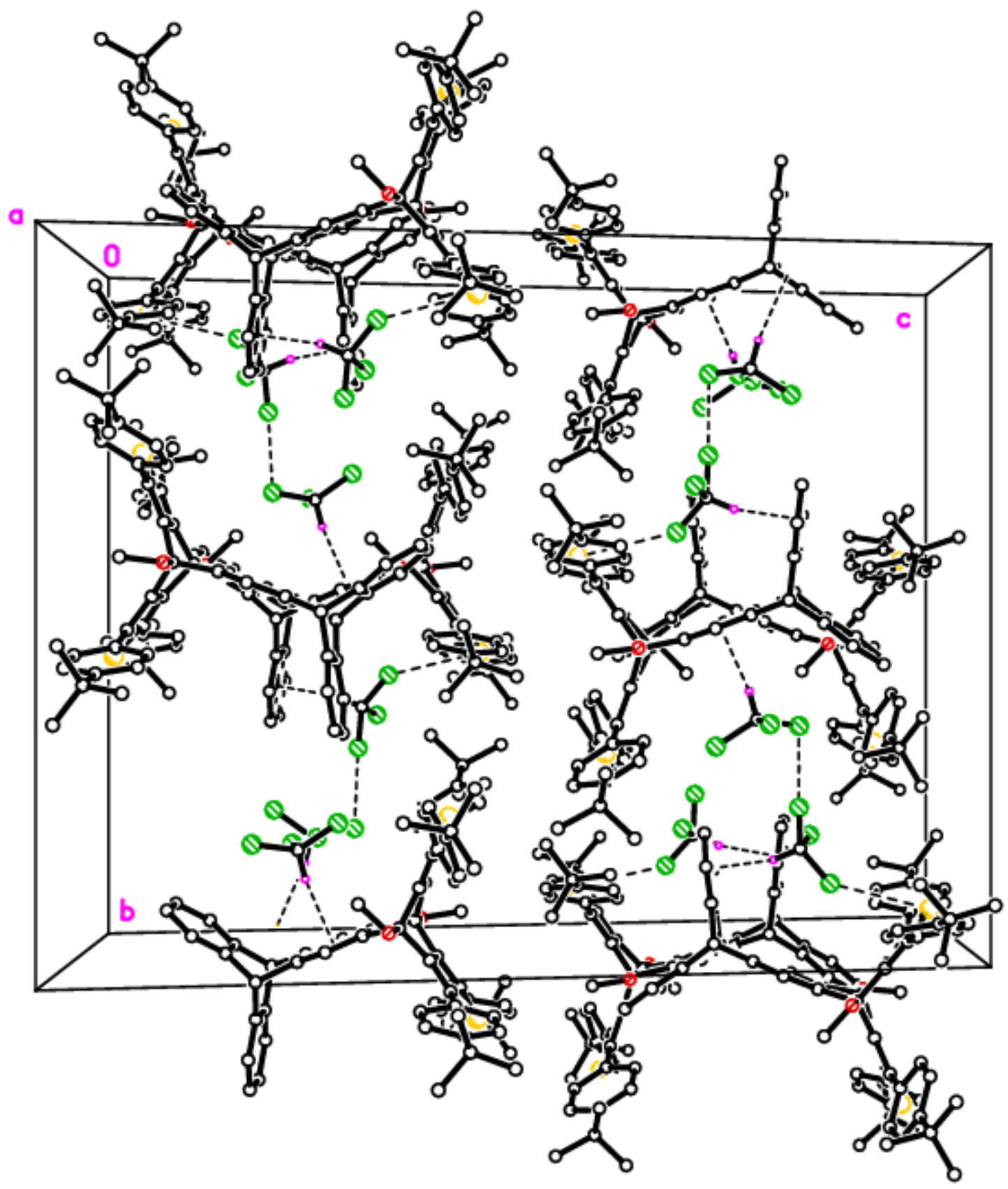

Fig S3. Packing plot for **2**·1.5($CHCl_3$)·3(CH_3OH) viewed parallel to *a*.

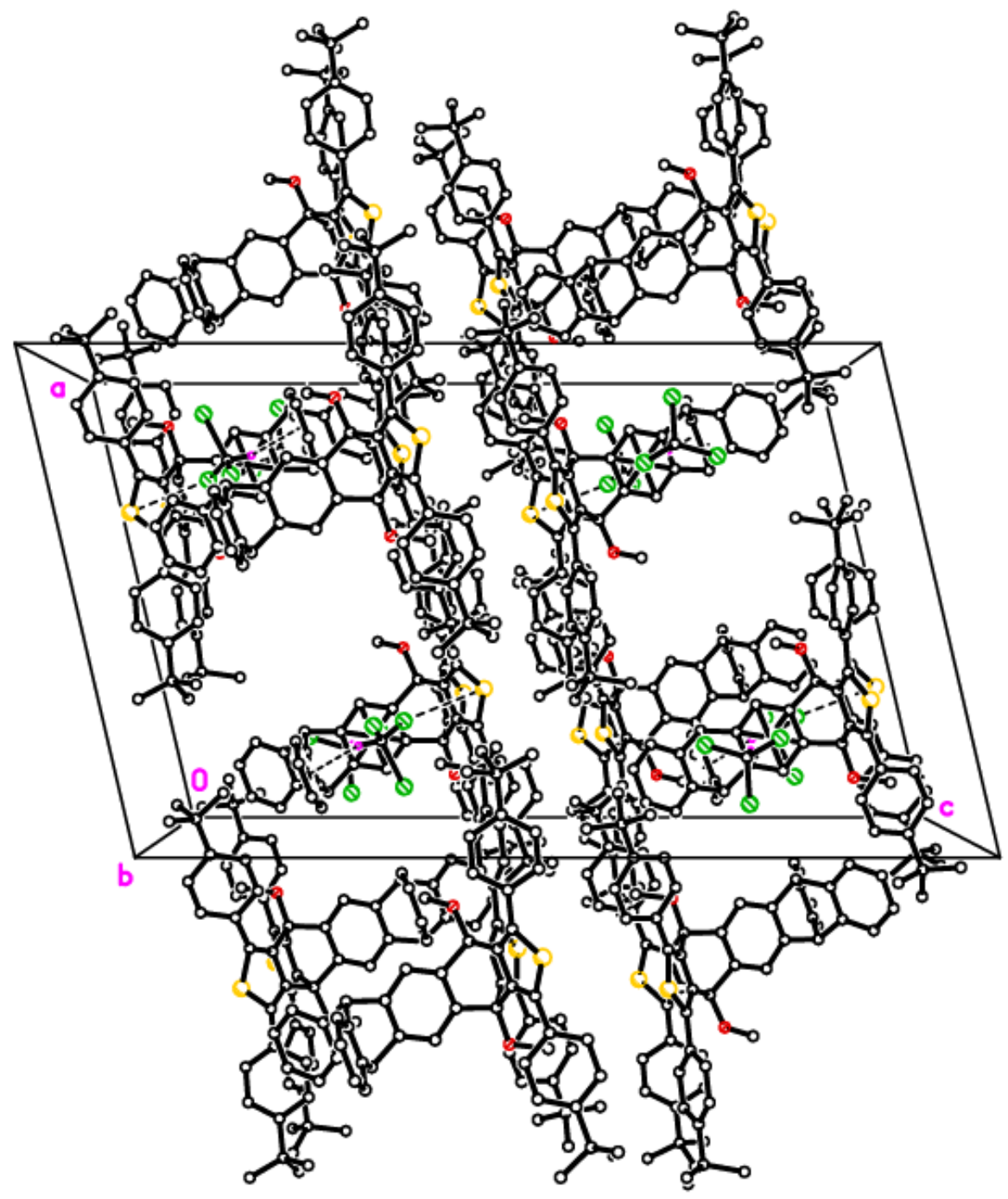

Fig S4. Packing plot for **2**·1.5($CHCl_3$)·3(CH_3OH) viewed parallel to *b*; disordered MeOH fills the 'void' spaces.

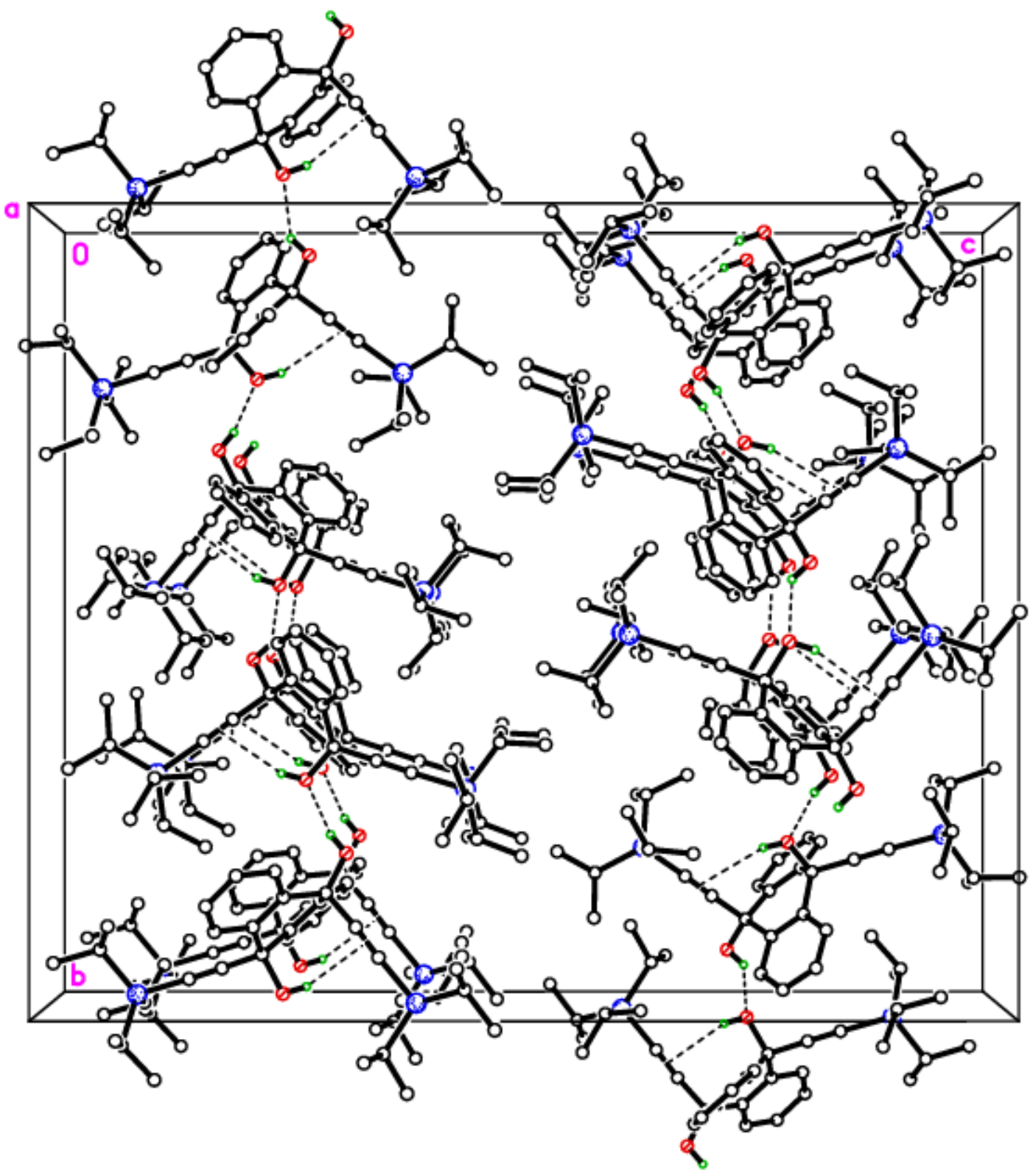

Fig S5. Packing plot for *trans*-**10** viewed parallel to *a* showing H-bonded 1D chains of molecules in the *b* direction.

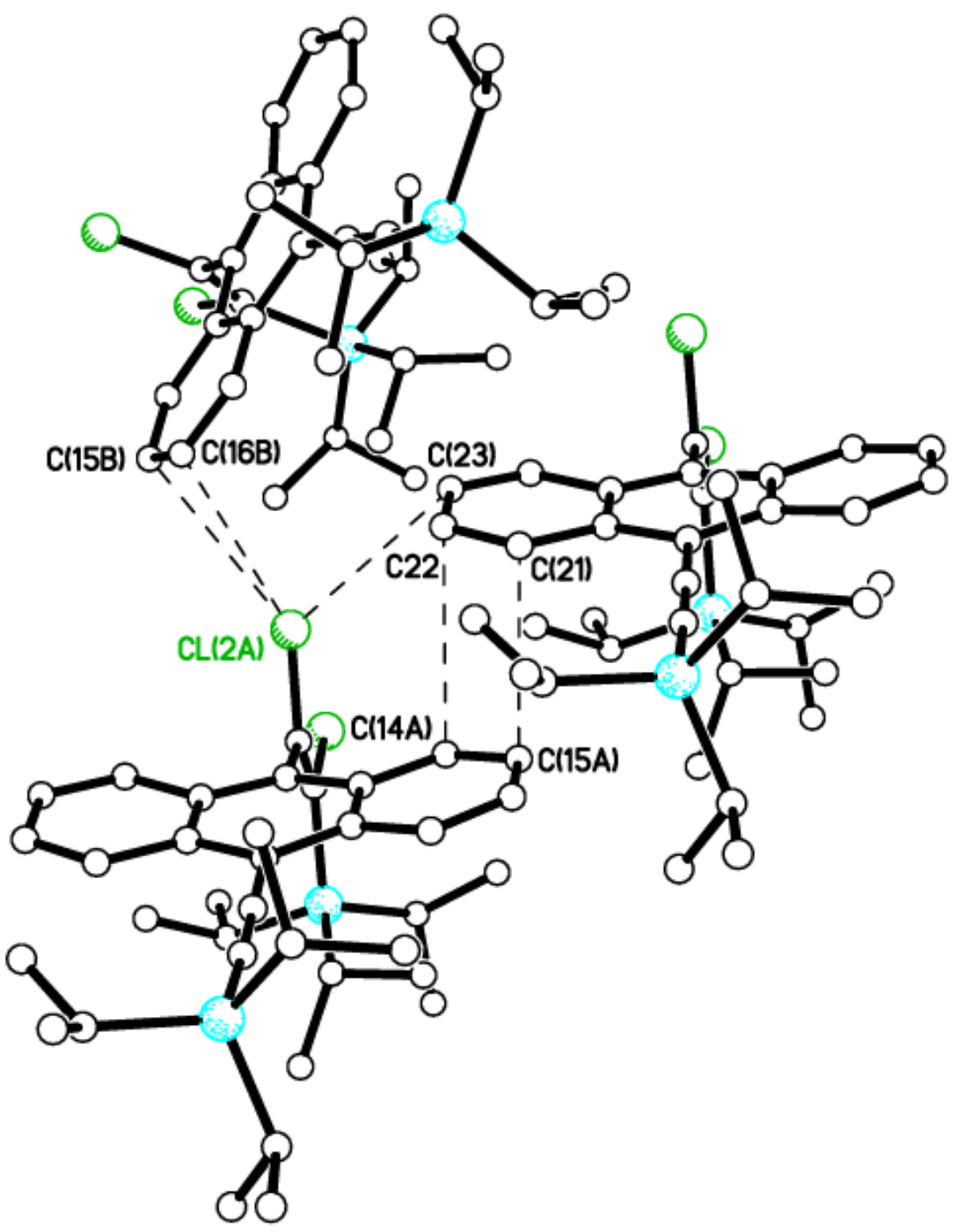

Fig S6. Plot of three molecules of **12** showing short-contacts between molecules.

Table S2: Crystal data and experimental details for **2**·1.5($CHCl_3$)·3(CH_3OH)

CCDC Deposition Number	2545297
Crystal data	
Chemical formula	$C_{72}H_{70}O_2S_2$·1.5($CDCl_3$)·3(CH_4O)
M_r	1308.08
Crystal system, space group	Monoclinic, *C*2/*c*
Temperature (K)	100
a, *b*, *c* (Å)	19.1538 (3), 23.8760 (4), 31.3928 (4)
β (°)	103.1390 (14)
V ($Å^3$)	13980.6 (4)
Z	8
Radiation type	Cu *K*α
μ (mm^{-1})	2.66
Crystal size (mm^3)	0.34 × 0.22 × 0.02
Data collection	
Diffractometer	Rigaku 007HF equipped with Varimax confocal mirrors and an AFC11 goniometer and HyPix 6000 detector
Absorption correction	Multi-scan. *CrysAlis PRO* 1.171.41.93a (Rigaku Oxford Diffraction, 2020). Empirical absorption correction using spherical harmonics, implemented in SCALE3 ABSPACK scaling algorithm.
T_{min}, T_{max}	0.794, 1.000
No. of measured, independent and observed [$I > 2\sigma(I)$] reflections	86693, 12740, 9390
R_{int}	0.106
$(\sin \theta/\lambda)_{max}$ ($Å^{-1}$)	0.602
Refinement	
$R[F^2 > 2\sigma(F^2)]$, $wR(F^2)$, S	0.088, 0.277, 1.05
No. of reflections	12740
No. of parameters	874
No. of restraints	516
H-atom treatment	H-atom parameters constrained
	$w = 1/[\sigma^2(F_o^2) + (0.1711P)^2 + 18.9083P]$ where $P = (F_o^2 + 2F_c^2)/3$
$\Delta\rangle_{max}$, $\Delta\rangle_{min}$ (e $Å^{-3}$)	1.16, -0.54

Table S3: Crystal data and experimental details for *trans*-**10**

CCDC Deposition Number	2545299
Crystal data	
Chemical formula	$C_{36}H_{52}O_2Si_2$
M_r	572.95
Crystal system, space group	Monoclinic, $P2_1/n$
Temperature (K)	150
a, *b*, *c* (Å)	8.6455 (10), 25.768 (3), 31.190 (3)
β (°)	90.107 (2)
V (Å^3)	6948.4 (13)
Z	8
Radiation type	Mo $K\alpha$
μ (mm^{-1})	0.13
Crystal size (mm^3)	0.76 × 0.35 × 0.05
Data collection	
Diffractometer	Bruker *APEX* 2 CCD area detector diffractometer
Absorption correction	Multi-scan *SADABS* v2016/2, Sheldrick, G.M., (2014)
T_{min}, T_{max}	0.907, 0.994
No. of measured, independent and observed [$I > 2\sigma(I)$] reflections	72633, 17421, 12580
R_{int}	0.048
$(\sin \theta/\lambda)_{max}$ (Å^{-1})	0.669
Refinement	
$R[F^2 > 2\sigma(F^2)]$, $wR(F^2)$, S	0.045, 0.119, 1.04
No. of reflections	17421
No. of parameters	778
No. of restraints	108
H-atom treatment	H atoms treated by a mixture of independent and constrained refinement
$\Delta\rangle_{max}$, $\Delta\rangle_{min}$ (e Å^{-3})	0.44, -0.43

Table S4: Crystal data and experimental details for *cis*-**10**

CCDC Deposition Number	2545298
Crystal data	
Chemical formula	$C_{36}H_{52}O_2Si_2$
M_r	572.95
Crystal system, space group	Monoclinic, $P2_1/n$
Temperature (K)	150
a, *b*, *c* (Å)	8.3241 (11), 31.043 (4), 13.6716 (18)
β (°)	95.487 (2)
V (Å^3)	3516.6 (8)
Z	4
Radiation type	Mo $K\alpha$
μ (mm^{-1})	0.13
Crystal size (mm^3)	0.47 × 0.41 × 0.21
Data collection	
Diffractometer	Bruker *APEX* 2 CCD area detector diffractometer
Absorption correction	Multi-scan. *SADABS* v2016/2, Sheldrick, G.M., (2016)
T_{min}, T_{max}	0.942, 0.973
No. of measured, independent and observed [$I > 2\sigma(I)$] reflections	40736, 10728, 7950
R_{int}	0.037
$(\sin \theta/\lambda)_{max}$ (Å^{-1})	0.716
Refinement	
$R[F^2 > 2\sigma(F^2)]$, $wR(F^2)$, S	0.044, 0.124, 1.08
No. of reflections	10728
No. of parameters	379
H-atom treatment	H atoms treated by a mixture of independent and constrained refinement
$\Delta\rangle_{max}$, $\Delta\rangle_{min}$ (e Å^{-3})	0.41, -0.26

Table S5: Crystal data and experimental details for **12**

CCDC Deposition Number	2545300
Crystal data	
Chemical formula	$C_{36}H_{50}Cl_2Si_2$
M_r	609.84
Crystal system, space group	Monoclinic, $P2_1/c$
Temperature (K)	150
a, b, c (Å)	29.321 (6), 7.7414 (15), 15.404 (3)
β (°)	99.976 (3)
V (Å^3)	3443.6 (12)
Z	4
Radiation type	Mo $K\alpha$
μ (mm^{-1})	0.28
Crystal size (mm^3)	0.85 × 0.59 × 0.09
Data collection	
Diffractometer	Bruker *APEX* 2 CCD area detector diffractometer
Absorption correction	Multi-scan *SADABS* v2016/2, Sheldrick, G.M., (2014)
T_{min}, T_{max}	0.817, 0.978
No. of measured, independent and observed [$I > 2\sigma(I)$] reflections	35320, 9624, 8102
R_{int}	0.046
$(\sin \theta/\lambda)_{max}$ (Å^{-1})	0.696
Refinement	
$R[F^2 > 2\sigma(F^2)]$, $wR(F^2)$, S	0.078, 0.181, 1.19
No. of reflections	9624
No. of parameters	373
H-atom treatment	H-atom parameters constrained
$\Delta\rangle_{max}$, $\Delta\rangle_{min}$ (e Å^{-3})	1.08, -0.65

References

1) M. S. Taylor and T. M. Swager, *Org. Lett.*, 2007, **9**, 3695–3697
2) M. Srinivasan, S. Sankararaman, H. Hopf and B. Varghese, *Eur. J. Org. Chem.*, 2003, **2003**, 660–665
3) G. Griffini, J. D. Douglas, C. Piliego, T. W. Holcombe, S. Turri, J. M. J. Fréchet and J. L. Mynar, *Adv. Mater.*, 2011, **23**, 1660–1664.
4) P. Chao, M. Guo, Y. Zhu, H. Chen, M. Pu, H. H. Huang, H. Meng, C. Yang and F. He, *Macromolecules*, 2020, **53**, 2893–2901.
5) G. M. Sheldrick, *Acta Cryst*. 2015, **A71**, 3-8.
6) G. M. Sheldrick, *Acta Cryst*. 2015, **C71,** 3-8.
7) A. L. Spek, Acta Cryst., 2015, **C71**, 9-18.
8) P. v. d. Sluis and A. L. Spek, *Acta Cryst*. 1990, **A46**, 194-201.
